# Novel serotonin transporter regulators: Natural aristolane- and nardosinane- types of sesquiterpenoids from *Nardostachys chinensis* Batal

**DOI:** 10.1038/s41598-017-15483-6

**Published:** 2017-11-08

**Authors:** Ying-Peng Chen, Shu-Song Ying, Hong-Hong Zheng, Yan-Ting Liu, Zhong-Ping Wang, Hu Zhang, Xu Deng, Yi-Jing Wu, Xiu-Mei Gao, Tian-Xiang Li, Yan Zhu, Yan-Tong Xu, Hong-Hua Wu

**Affiliations:** 1 Tianjin State Key Laboratory of Modern Chinese Medicine, Tianjin Key Laboratory of Chemistry and Analysis of Traditional Chinese Medicine, Institute of Traditional Chinese Medicine, Tianjin University of Traditional Chinese Medicine, Tianjin, 300193 China; 2 Chinese Medicine Research Center, Tianjin University of Traditional Chinese Medicine, Tianjin, 300193 China

## Abstract

Serotonin transporter (SERT) is a classic target of drug discovery for neuropsychiatric and digestive disorders, and against those disorders, plants of Nardostachys genus have been valued for centuries in the systems of Traditional Chinese Medicine, Ayurvedic and Unani. Herein, chemical investigation on the roots and rhizomes of *Nardostachys chinensis* Batal. led to the isolation of forty sesquiterpenoids including six new aristolane-type sesquiterpenoids and six new nardosinane-type sesquiterprenoids. Their structures were elucidated by extensive spectroscopic methods, combined with analyses of circular dichroism and single-crystal X-ray diffraction data. To explore natural product scaffolds with SERT regulating activity, a high-content assay for measurement of SERT function *in vitro* was conducted to evaluate the SERT regulating properties of these isolates. In conclusion, eleven compounds could be potential natural product scaffolds for developing drug candidates targeting SERT. Among which, kanshone C of aristolane-type sesquiterpenoid inhibited SERT most strongly, while desoxo-nachinol A of nardosinane-type sesquiterpenoid instead enhanced SERT potently.

## Introduction

Serotonin transporter (SERT) is a classic target of drug discovery for neuropsychiatric and digestive disorders. At serotonin synapses in the central nervous system, SERT is responsible for the reuptake of 5-hydroxytryptamine into presynaptic neurons, and it is implicated in the occurrence of mood disorders, for instance, depression, anxiety or obsessive-compulsive disorder^1^. At enterochromaffin cells in the digestive system, SERT inactivates the stimulant effects of 5-hydroxytryptamine on gastrointestinal tract mucosa, and it plays important roles in the pathophysiology of digestive disorders such as irritable bowel syndrome, slow transit constipation and functional abdominal bloating^2,3^. To screen SERT activity of the candidate compounds, the high-content assay for measurement of SERT function based on human embryonic kidney 293 cell line stably expressing human SERT (hSERT-HEK) and the fluorescent substrate 4-[4-(dimethylamino)phenyl]-1-methylpyridinium (APP^+^) has been established^4,5^, and this novel method is more feasible in practice than the traditional isotope labeling uptake assay.

**Figure 1:**
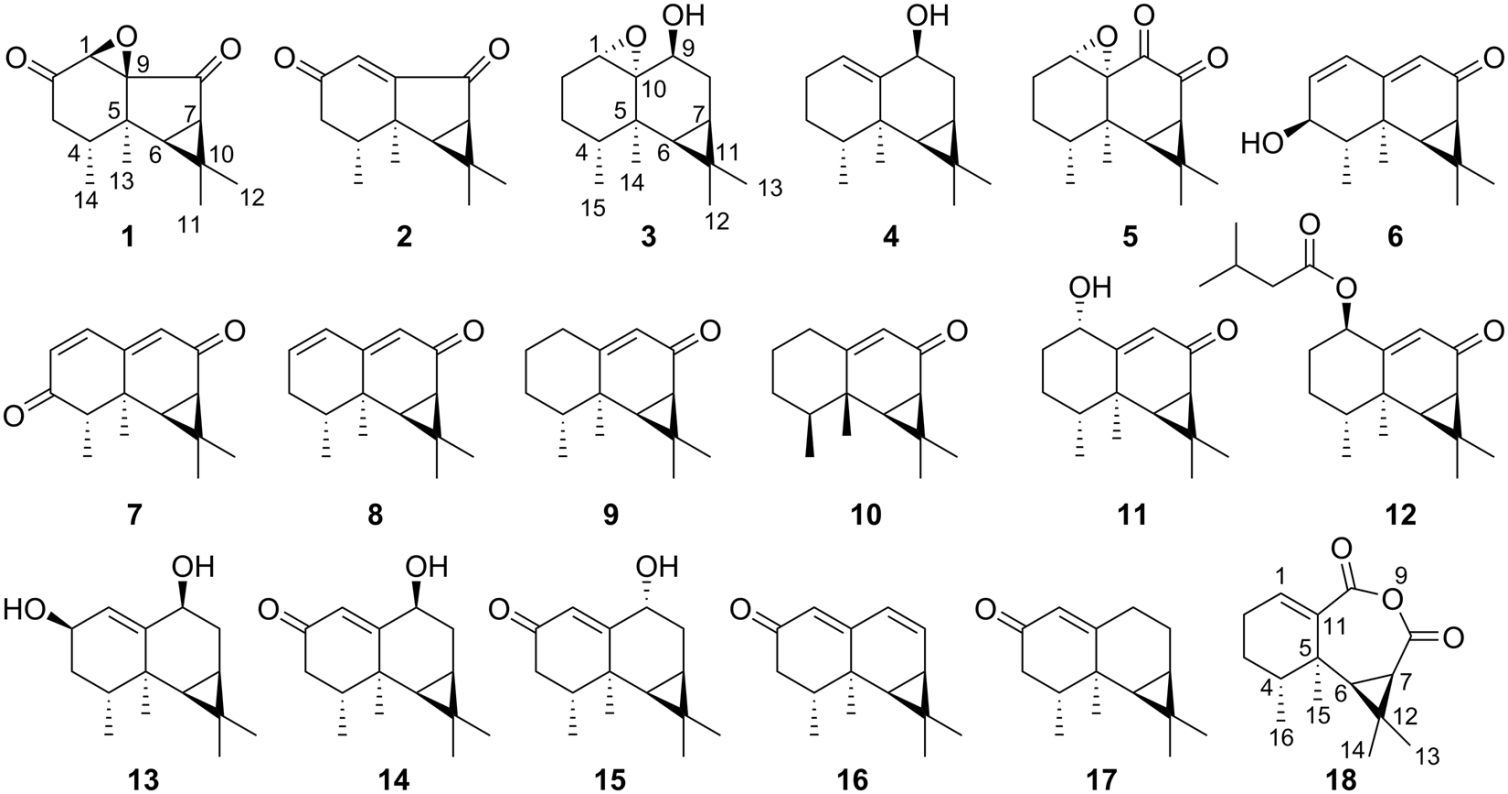
Aristolane-type sesquiterpenoids from *N. chinensis* Batal.

**Figure 2:**
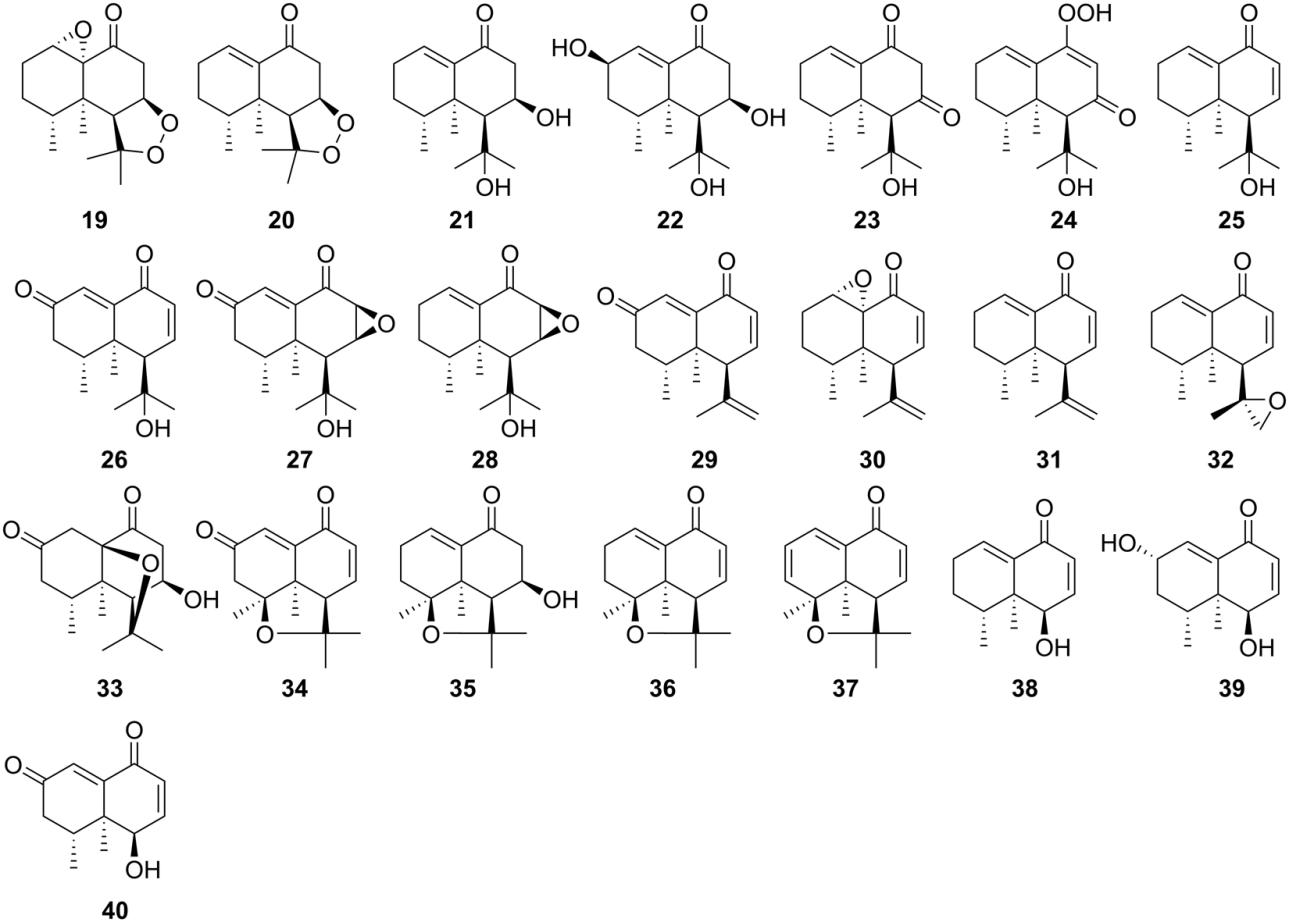
Nardosinone-type sesquiterpenoids from *N. chinensis* Batal.

To identify novel SERT regulators from natural products, *Nardostachys chinensis* Batal. (NCB) has been studied. NCB is mainly distributed in Sichuan, Gansu, Qinghai and Xizang areas in China. The root and rhizome of NCB have been used as both herbal drugs and functional foods for centuries to treat digestive disorders in traditional Chinese medicine^6^. Modern pharmacological studies demonstrated that NCB show bioactivities in against depression, arrhythmia, convulsion, myocardial ischemia and hypertension^7^. This plant was enriched with bioactive sesquiterpenoids, among which aristolane-, nardosinane-, and guaiane- types of sesquiterpenoids were the representative constituents^8,9^. Herein, we report the isolation, structure elucidation and effects on SERT function of six new and twelve known aristolane-type sesquiterpenoids (Fig. 1), together with six new and sixteen known nardosinane-type sesquiterpenoids (Fig. 2) from NCB.

## Results and Discussion

### Structure identification

The 70% aqueous ethanol extract of the air-dried roots and rhizomes of *Nardostachys chinensis* Batal. was subjected to various modern chromatographic isolation (including preparative thin layer chromatography, silica gel/Sephadex LH-20 column chromatography, and reversed-phase C_18_ preparative/semipreparative high performance liquid chromatography) to give six new (compounds **3**, **6**, **7**, **11**, **14** and **18**) and twelve known aristolane-type sesquiterpenoids (Fig. 1), together with six new (compounds **19**, **22**–**24**, **26**, and **30**) and sixteen known nardosinane-type sesquiterpenoids (Fig. 2). Based on the comparison of spectroscopic data with those previously reported, those known compounds were identified as nardoaristolone C (**1**)^10^, nardoaristolone B (**2**)^11^, 1(10)-aristolen-9*β*-ol (**4**)^12^, kanshone C (**5**)^13^, kanshone H (**8**)^14^, (−)-aristolone (**9**)^15^, (−)-(14*β*,15*β*)-aristolone (**10**)^16^, kanshone F (**12**)^8^, kanshone G (**13**)^8^, debilon (**15**)^17^, nardostachone (**16**)^18^, 1(10)-aristolen-2-one (**17**)^19^, nardosinone (**20**)^17^, nardosinonediol (**21**)^17^, kanshone A (**25**)^17^, kanshone E (**27**)^20^, isonardosinone (**28**)^20^, nardosinanone H (**29**)^9^, (4 *S*,4a*R*,5 *R*)-4a,5-dimethyl-4-(1-methylethenyl)-4a,5,6,7-tetrahydronaphthalen-1(4 *H*)-one (**31**)^21^, nardosinanone I (**32**)^9^, nardosinanone B (**33**)^22^, nardosinanone G (**34**)^9^, nardosinanone M (**35**)^10^, nardonoxide (**36**)^12^, nardosinanone F (**37**)^9^, desoxo-nachinol A (**38**)^17^, narchinol B (**39**)^23^ and narchinol A (**40**)^24^, respectively. The structures of the new compounds were deduced by analysis of extensive spectroscopic data [including HRESIMS, 1D/2D NMR, optical rotation and circular dichroism (CD) data].

Nardoaristolone C (**1**) was isolated as colorless needles. The ^1^H and ^13^C NMR data (Supplementary Table S3) revealed that the structure of **1** was similar to that of nardoaristolone B (**2**), except that the two olefinic carbons (C-1 and C-9) in **2** were replaced by the two oxygenated carbons in **1** [*δ*_C_ 60.1 (C-1) and *δ*_C_ 69.4 (C-9), *δ*_H_ 3.90 (1 H, s, H-1)], indicating the existence of an oxiran ring adjacent to carbonyl in **1**. Analysis of the HSQC and HMBC spectroscopic data supported the assignments of its 1D NMR data, which were identical with those data recently reported by other groups in this April.

The relative configuration of compound **1** was revealed by single-crystal X-ray diffraction (Mo K*α*) data, together with the NOESY correlations between *δ*_H_ 1.97 (H-6) and *δ*_H_ 1.04 (H_3_-13), and between *δ*_H_ 1.56 (H_3_-12) and *δ*_H_ 2.57 (H-4)/1.20 (H_3_-11). For X-ray crystallographic analysis of light atom structures (those containing only C, H, N or O), if a heavier element (such as chlorine, bromine or sulfur) is not contained in the crystals, the anomalous scattering with molybdenum (Mo) radiation is always too small to assign the absolute configuration^25^. Herein, the absolute configuration of **1** was then assigned by comparing its CD data with those of **2** (Supplementary Fig. S79), both sharing a positive Cotton effect at 215 ± 5 nm and a negative Cotton effect at 255 ± 2 nm, which were identical with the reported data^10^.

**Figure 3:**
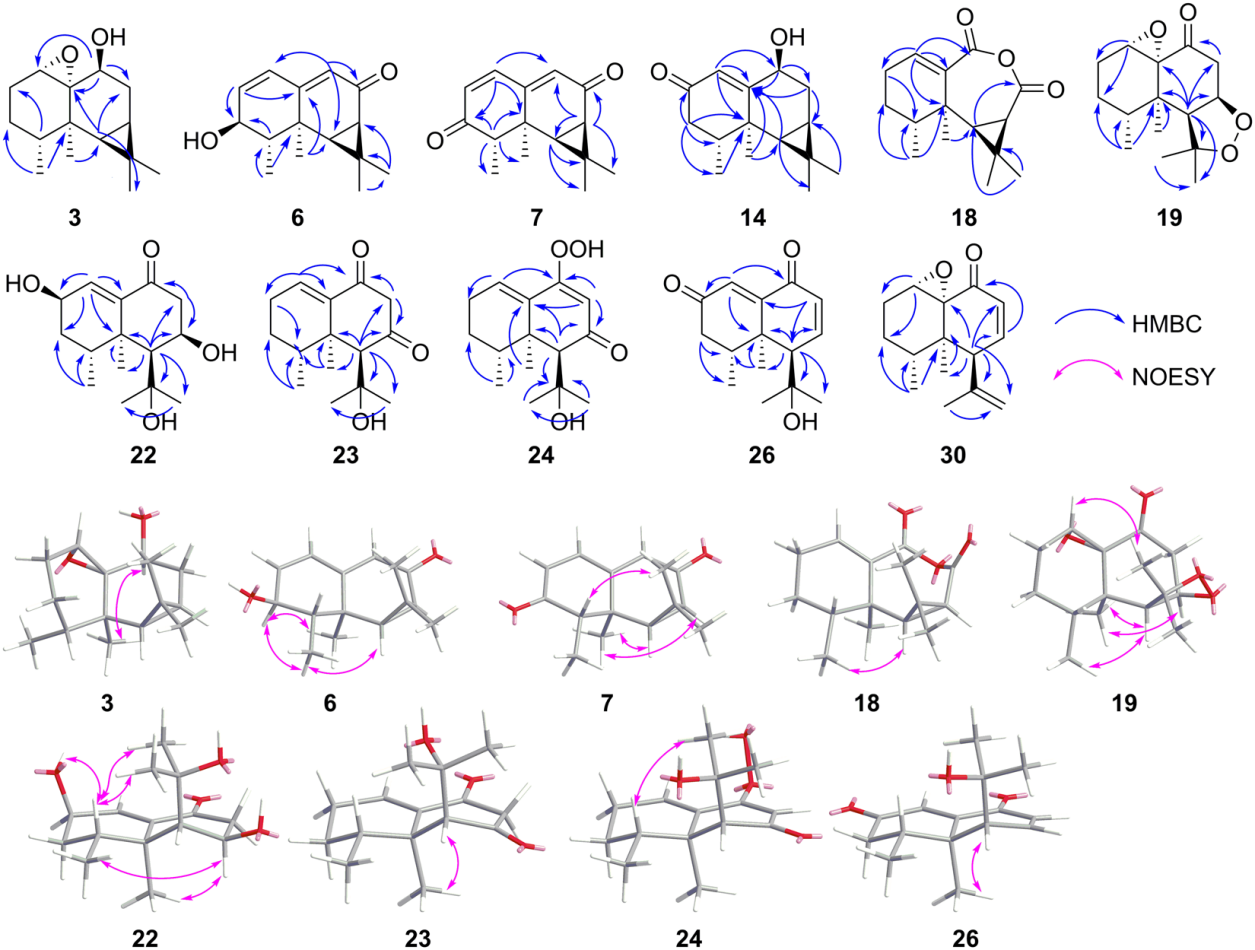
Key HMBC and NOESY correlations for the new sesquiterpenoids from *N. chinensis* Batal.

Nardoaristol (**3**) was isolated as a colorless oil, and epoxynardosinone (**19**) was isolated as colorless crystals. Their molecular formulas were confirmed respectively to be C_15_H_24_O_2_ and C_15_H_22_O_4_ by analysis of their NMR and ESIMS data. The ^1^H and ^13^C NMR data (Supplementary Table S3 and Table 1) demonstrated that the structures of **3** and **19** were similar to those of 1(10)-aristolen-9*β*-ol (**4**) and nardosinone (**20**), except that the olefinic carbons (C-1 and C-10) in **4** and **20** were replaced by the oxygenated carbons of two oxiran rings in **3** [*δ*_C_ 57.4 (C-1) and *δ*_C_ 66.7 (C-9), *δ*_H_ 3.47 (1 H, d, *J* = 2.4 Hz, H-1)] and **19** [*δ*_C_ 60.2 (C-1) and *δ*_C_ 61.9 (C-10), *δ*_H_ 3.92 (1 H, br s, H-1)], respectively. The deduction was further confirmed by analysis of the HSQC and HMBC spectroscopic data and the relative configurations of **3** and **19** were assigned by the 2D NOESY experiments (Fig. 3). Similarly, the structure of epoxynardosinanone H (**30**) was established by comparison of its 1D/2D NMR spectroscopic data with those of nardosinanone H (**29**).

**Table 1:** ^13^C NMR (100 MHz, CDCl_3_) data for sesquiterpenoids 3, 6, 7, 14, 18–19, 22–24, 26 and 30.

No	**3**	**6**	**7**	**14**	**18** ^*a*^	**19**	**22** ^*b*^	**23**	**24** ^*b*^	**26**	**30**
	*δ*_C_	*δ*_C_	*δ*_C_	*δ*_C_	*δ*_C_	*δ*_C_	*δ*_C_	*δ*_C_	*δ*_C_	*δ*_C_	*δ*_C_
1	57.4	128.4	142.4	120.9	137.4	60.2	134.0	138.4	130.5	128.5	62.5
2	25.8	139.2	131.6	199.3	24.1	25.3	61.5	26.0	25.5	199.8	26.1
3	24.3	71.4	199.6	42.1	26.5	23.4	35.5	26.6	25.7	42.5	24.1
4	37.5	44.1	48.7	36.9	37.4	32.9	27.4	33.0	32.1	34.5	34.0
5	36.3	38.5	40.5	40.6	41.4	37.1	40.7	39.5	40.4	43.7	38.6
6	34.7	38.1	38.2	32.7	46.4	61.4	53.1	67.3	65.6	54.1	56.0
7	18.4	36.2	36.2	18.4	31.1	77.7	66.9	206.3	198.8	152.2	150.0
8	25.8	196.6	195.8	30.1	176.1	42.0	46.2	52.4	101.8	129.3	129.6
9	65.3	124.5	129.1	67.4	—	204.9	200.8	195.5	166.5	186.6	194.9
10	66.7	158.6	156.3	174.7	171.8	61.9	143.5	140.7	136.4	157.5	62.6
11	18.0	25.8	26.4	19.4	141.2	84.3	75.9	72.0	71.4	76.3	142.2
12	29.4	29.5	29.5	17.5	30.3	28.8	30.9	31.5	32.9	16.9	118.1
13	18.8	15.8	16.1	29.2	16.6	22.3	36.0	31.1	25.2	33.1	18.3
14	19.8	23.2	25.3	22.8	32.6	19.8	23.3	24.5	22.8	21.4	18.3
15	16.2	11.2	7.9	15.4	20.3	15.7	16.4	16.2	16.7	24.8	15.4
16	—	—	—	—	16.6	—	—	—	—	—	—

^*a*^Measured in CD_3_OD, ^*b*^Measured in DMSO-*d*_6_.

3-Hydroxylkanshone H (**6**) was isolated as a colorless oil, and 3-oxokanshone H (**7**) was isolated as a white amorphous powder. Analysis of their ESIMS and NMR data established the molecular formulas to be C_15_H_20_O_2_ and C_15_H_18_O_2_. On the basis of the HSQC and HMBC spectra, the structures of **6** and **7** were elucidated as 3-hydroxylaristol-1,9-dien-8-one [*δ*_H_ 4.04 (1 H, d, *J* = 9.6 Hz)/*δ*_C_ 71.4, H-3/C-3 in **6**] and aristol-1,9-dien-3,8-dione (*δ*_C_ 199.6, C-3 in **7**), indicating that they were 3-hydroxyl and 3-oxo derivatives of kanshone H (**8**), respectively. The *β* configuration of the 3-hydroxyl group in **6** was deduced from the key NOESY correlations (Fig. 3) between H-3 and H_3_-14 (*δ*_H_ 1.14)/H_3_-15 (*δ*_H_ 1.27).

Based on the above deduced relative configurations, the absolute configurations of the new compounds **6**, **7**, **19** and **30** were proposed by comparing their experimental CD data with those of the known aristolane-type and nardosinane-type sesquiterpenoids isolated from NCB (Supplementary Fig. S79), which normally share the same intrinsic absolute configurations for stereogenic carbons C-4, 5, 6 and 7, owing to the relatively conservative biogenic pathway of sesquiterpenoids in NCB. The structures of compounds **6**–**8** possess similar chromophores^26^, and then they showed similar patterns of CD curves as shown in Supplementary Fig. S79. When compared with **8**, 3-hydroxyl substitution seemed to weaken the positive cotton effect around 245 nm in **6**, while 3-ketone substitution was prone to reverse the shoulder peak around 300 nm to be a “valley”-style curve in **7**. Assignments of the absolute configurations of **3** was fairly challenging owing to its structure without any cyclic ketone group. Considering that **3** was a 1(10)-epoxidation derivative of the known aristolane-type sesquiterpenoid (**4**) and its CD spectrum also showed negative Cotton effects (<215 nm), the absolute configuration of **3** was proposed as shown in Fig. 1.

Based on the comparisons of 1D/2D NMR data, 1-hydroxylaristolone (**11**) and 9*β*-debilon (**14**) were found out to be two new stereoisomers of the reported structures axinysone B^27^ and debilon (**15**), respectively. 1D NMR data of **11** was exactly the same as those reported for axinysone B, but the optical rotation and CD data of **11** was opposite to those of axinysone B, indicating that the two compounds are a pair of enantiomers. The experimental CD spectrum of **11** was further compared with the computational ECD spectra of the (1 *S*,4 *R*,5 *R*,6 *S*,7 *R*)-**11a** and (1 *R*,4 *S*,5 *S*,6 *R*,7 *S*)-**11b** obtained by time-dependent density functional theory (TDDFT) quantum mechanics [B3LYP/6–31 G(d)]^28^. The excellent agreement of the experimental and computational ECD spectra (see Supplementary Fig. S80) confirmed the assignment of the absolute configuration of **11** as (1 *S*,4 *R*,5 *R*,6 *S*,7 *R*). **14** was elucidated as a 9*β*-hydroxyl epimer of **15** (debilon) by analysis and comparison of the spectroscopic data (including 1D/2D NMR, ORD and CD data) for compounds **14** and **15**.

Aristolanhydride (**18**) was isolated as a white amorphous powder with a molecular formula of C_15_H_20_O_3_, deduced by analysis of the HRESIMS (*m/z* 249.1469 [M + H]^+^, calcd for C_15_H_21_O_3_^+^, 249.1491) and NMR data. The ^1^H NMR spectrum of **18** revealed the existences of four methines [*δ*_H_ 6.65 (1 H, t, *J* = 3.9 Hz), 2.10 (1 H, m), 1.37 (1 H, d, *J* = 9.9 Hz) and 1.42 (1 H, d, *J* = 9.9 Hz)], two methylenes [*δ*_H_ 2.15 (2 H, m), 1.82 (1 H, td, *J* = 6.7, 3.1 Hz) and 1.45 (1 H, m)] and four methyls [*δ*_H_ 1.18 (3 H, s), 1.33 (3 H, s), 1.44 (3 H, s) and 0.89 (3 H, d, *J* = 6.9 Hz)] in **18**. Furthermore, the ^13^C NMR spectrum of **18** suggested a double bond (*δ*_C_ 141.2 and 137.4) and two ester carbonyls (*δ*_C_ 176.1 and 171.8) were included in the structure of **18**. HMBC correlations from *δ*_H_ 6.65 (H-1) to *δ*_C_ 171.8 (C-10), 141.2 (C-11), 41.4 (C-5) and 24.1 (C-2), from *δ*_H_ 0.89 (H_3_-16) to *δ*_C_ 41.4, 37.4 (C-4) and 26.5 (C-3), from *δ*_H_ 1.44/1.18 (H_3_-13/14) to *δ*_C_ 46.4 (C-6) and 30.3 (C-12), from *δ*_H_ 1.37 (H-6) to *δ*_C_ 176.1 (C-8) and from *δ*_H_ 1.33 (H_3_-15) to *δ*_C_ 46.4 then established the planar structure of **18**, a rare 8,9-cleavage anhydride derivative of aristolane-type sesquiterpenoid. The stereochemistry problem of **18** was solved by NOESY experiment and comparison of its experimental ECD spectrum with the computational ones (see Supplementary Fig. S81).

The structures of other four new nardosinane-type sesquiterpenoids, nardosinonetriol (**22**), 7-oxonardosinone (**23**), 7-oxonardosinoperoxide (**24**) and 2-oxokanshone A (**26**), were established by comparing their NMR data (Supplementary Tables S3–S4 and Table 1) with those of known compounds **21** (nardosinonediol) and **25** (kanshone A). Compound **24** was deduced as a peroxide compound on the basis of its positive HRESIMS (*m/z* 267.1589 [M + H]^+^, cald for C_15_H_23_O_4_^+^, 267.1591) and NMR data. Furthermore, the absolute configurations of these compounds were all proposed as shown in Fig. 2 based on the consideration of conservative biogenic pathway for nardosinane-type sesquiterpenoids, assisted by 2D NOESY experiments as shown in Fig. 3. The plausible biosynthetic pathways for aristolane- and nardosinane- types of sesquiterpenoids were proposed as shown in Supplementary Figures S82–S83.

### SERT regulating activities

As shown in Table 2, compounds **2**, **4**, **6**–**8**, **11**, **16**, **19**, **23**–**24**, **27**–**29**, **32**–**33**, **36**, **38** and **40** enhanced SERT activity while compounds **5**, **12**–**13**, **17**, **20**–**21**, **30**, **35** and **37** inhibited SERT activity. Compounds **1**, **9**, **15**, **18**, **22**, **25**, **26**, **31** and **34** did not show any SERT activity meanwhile compounds **3**, **10**, **14** and **39** were not tested due to insufficient amount. For the SERT enhancers, nardoaristolone B (**2**), nardonoxide (**36**) and desoxo-nachinol A (**38**) showed potent effects, among which a 4,11-*O*-briged nardosinane-type sesquiterpenoid (nardonoxide, **36**) with a 5/6/6 tricyclic ring system showed the strongest effect; 1(10)-aristolen-9*β*-ol (**4**), kanshone H (**8**), nardostachone (**16**), 7-oxonardosinoperoxide (**24**), kanshone E (**27**) and nardosinanone H (**29**) were in the middle; and 3-hydroxylkanshone H (**6**), 3-oxokanshone H (**7**), 1-hydroxylaristolone (**11**), epoxynardosinone (**19**), 7-oxonardosinone (**23**), isonardosinone (**28**), nardosinanone I (**32**) and nardosinanone B (**33**) showed weak effects, however, **8** exhibited stronger effect than its 3-hydroxyl derivative (**6**) and 3-oxo derivative (**7**), **23** exhibited weaker effect than its 9-peroxide derivative (**24**), and **28** showed weaker effect than its 2-oxo derivative (**27**). For the SERT inhibitors, kanshone C (**5**), nardosinone (**20**) and nardosinonediol (**21**) significantly inhibited SERT activity. Further analysis suggested that 1(10) or 1(9)-epoxidation of the double bond (as shown in cases of **1**/**2**, **19**/**20** and **30**/**31**) seems to inactivate or reverse the aristolane-type and nardosinane-type sesquiterpenoids’ regulation effects on SERT activity.

**Table 2:** Effects of the compounds identified from NCB on SERT activity.

					
Type	Compound	Concentration (*μ*M)^*a*^			SERT activity
		0.1	1.0	10.0	
**I**	**2**: 1(9)-en-2,8-dione	1.13 ± 0.05**	1.26 ± 0.05***	1.41 ± 0.03***	↑↑↑^b^
	**1**: 1*β*,9*β*-epoxy-2,8-dione	1.04 ± 0.02	1.06 ± 0.03	1.03 ± 0.02	N.A.
**II**	**5**: 1*α*,10*α*-epoxy-8,9-dione	0.93 ± 0.04*	0.87 ± 0.03***	0.36 ± 0.02***	↓↓↓^c^
	**13**: 2*β*,9*β*-dihydroxy-1(10)-en	0.94 ± 0.02**	0.96 ± 0.01*	0.93 ± 0.02***	↓↓
	**17**: 1(10)-en-2-one	0.96 ± 0.01**	0.96 ± 0.01*	0.93 ± 0.01***	↓↓
	**12**: 1*β*-hydroxy-9(10)-en-8-one	0.97 ± 0.01	1.03 ± 0.01	0.95 ± 0.01**	↓
	**4**: 9*β*-hydroxy-1(10)-en	1.23 ± 0.03***	1.26 ± 0.02***	1.21 ± 0.03***	↑↑↑
	**8**: 1(2),9(10)-dien-8-one	1.06 ± 0.02	1.09 ± 0.02**	1.13 ± 0.03***	↑↑
	**16**: 1(10),8(9)-dien-2-one	1.01 ± 0.03	0.99 ± 0.02	1.14 ± 0.01***	↑↑
	**7**: 1(2),9(10)-dien-3,8-dione	0.97 ± 0.01	1.06 ± 0.02**	1.09 ± 0.02***	↑
	**6**: 3*β*-hydroxy-1(2),9(10)-dien-8-one	1.02 ± 0.01	1.06 ± 0.02*	1.08 ± 0.03**	↑
	**11**: 1*α*-hydroxy-9(10)-en-8-one	1.06 ± 0.01*	1.03 ± 0.02	1.04 ± 0.02	↑
	**15**: 9*α*-hydroxy-1(10)-en-2-one	1.01 ± 0.02	1.04 ± 0.02	1.04 ± 0.01	N.A.^d^
	**9**: 9(10)-en-8-one	1.01 ± 0.02	1.04 ± 0.01	1.07 ± 0.02	N.A.
**III**	**18**: 1(11)-en	0.99 ± 0.02	0.98 ± 0.01	0.97 ± 0.02	N.A.
	**21**: 7*β*,11*β*-dihydroxy-1(10)-en-9-one	0.56 ± 0.05***	0.65 ± 0.03***	0.78 ± 0.03***	↓↓↓
	**20**: 7*β*,11-peroxy-1(10)-en-9-one	0.61 ± 0.04***	0.70 ± 0.03***	0.89 ± 0.02***	↓↓↓
	**30**: 1*α*,10*α*-epoxy-7(8),11(12)-dien-9-one	0.97 ± 0.01	0.92 ± 0.01***	0.93 ± 0.02***	↓↓
	**37**: 4*β*,11*β*-epoxy-1(10),2(3),7(8)-trien-9-one	0.97 ± 0.01*	0.96 ± 0.01**	0.97 ± 0.01*	↓↓
	**35**: 7*β*-hydroxy-4*β*,11*β*-epoxy-1(10)-en-9-one	0.97 ± 0.01	0.96 ± 0.02*	1.01 ± 0.01	↓↓
	**23**: 11-hydroxy-1(10)-en-7,9-dione	0.96 ± 0.01*	1.05 ± 0.01**	1.08 ± 0.02***	↓↑
	**36**: 4*β*,11*β*-epoxy-1(10),7(8)-dien-9-one	1.26 ± 0.02***	1.31 ± 0.02***	1.53 ± 0.05***	↑↑↑
	**27**: 11-hydroxy-7*β*,8*β*-epoxy-1(10)-en-2,9-dione	1.09 ± 0.02*	1.19 ± 0.02***	1.22 ± 0.04***	↑↑↑
	**29**: 1(10),7(8),11(12)-trien-2,9-dione	1.06 ± 0.04	1.11 ± 0.06**	1.16 ± 0.04***	↑↑
	**24**:				
**IV**	11-hydroxy-9-hydroxyperoxy-1(10),8(9)-dien-7-one	1.06 ± 0.02	1.11 ± 0.02***	1.12 ± 0.02***	↑↑
	**28**: 11-hydroxy-7*β*,8*β*-epoxy-1(10)-en-9-one	1.08 ± 0.02**	1.10 ± 0.01***	1.09 ± 0.02***	↑
	**19**: 1*α*,10*α*-epoxy-7*β*,11-peroxy-9-one	1.01 ± 0.01	0.99 ± 0.02	1.09 ± 0.02***	↑
	**32**: 11*α*,12*α*-epoxy-1(10),7(8)-dien-9-one	1.05 ± 0.01*	1.08 ± 0.02***	1.07 ± 0.02***	↑
	**33**: 7*β*-hydroxy-10*β*,11*β*-epoxy-2,9-dione	1.07 ± 0.01**	1.05 ± 0.01	1.08 ± 0.02**	↑
	**25**: 11-hydroxy-1(10),7(8)-dien-9-one	0.97 ± 0.01	0.97 ± 0.02	0.98 ± 0.01	N.A.
	**26**: 11-hydroxy-1(10),7(8)-dien-2,9-dione	0.97 ± 0.01	1.00 ± 0.02	1.01 ± 0.01	N.A.
	**34**: 4*β*,11*β*-epoxy-1(10),7(8)-dien-2,9-dione	1.01 ± 0.02	1.02 ± 0.01	1.00 ± 0.03	N.A.
	**31**: 1(10),7(8),11(12)-trien-9-one	1.00 ± 0.02	1.02 ± 0.01	1.03 ± 0.02	N.A.
	**22**: 2*β*,7*β*,11-trihydroxy-1(10)-en-9-one	1.06 ± 0.02	1.03 ± 0.02	1.04 ± 0.02	N.A.
**V**	**38**: 1(10),7(8)-dien-9-one	1.25 ± 0.03***	1.27 ± 0.05***	1.35 ± 0.02***	↑↑↑
	**40**: 1(10),7(8)-dien-2,9-dione	1.01 ± 0.01	1.05 ± 0.01**	1.00 ± 0.01	↑

^a^The values represent the mean ± S.E.M. of relative fluorescent intensity (RFI) from triplicate assays (n ≥ 9). RFI = (Intracellular APP^+^ fluorescent intensity_treatment_/Intracellular APP^+^ fluorescent intensity_control_), *p < 0.05; **p < 0.01; ***p < 0.001. ^b^Enhancement activity, ↑↑↑, RFI > 1.20, ↑↑, RFI > 1.10, ↑, RFI > 1.05. ^c^Inhibition activity, ↓↓↓, RFI < 0.90, ↓↓, RFI < 0.96, ↓, RFI < 0.98. ^d^N. A., no activity.

Both enhancer and inhibitor of SERT, interestingly, were identified in the two main types of sesquiterpenoids (subdivided into five subtypes I, II, III, IV and V) from NCB, and the SERT enhancers are richer in quantity than the SERT inhibitors. According to the proposed biosynthetic pathways (see Supplementary Figures S82–S83), it suggests that there exist conversions between SERT enhancers and SERT inhibitors in NCB. For instance, the SERT inhibitor kanshone C (**5**), and the SERT enhancers 1(10)-aristolen-9*β*-ol (**4**) and nardostachone (**16**), are derived from compound **4**.

**Figure 4:**
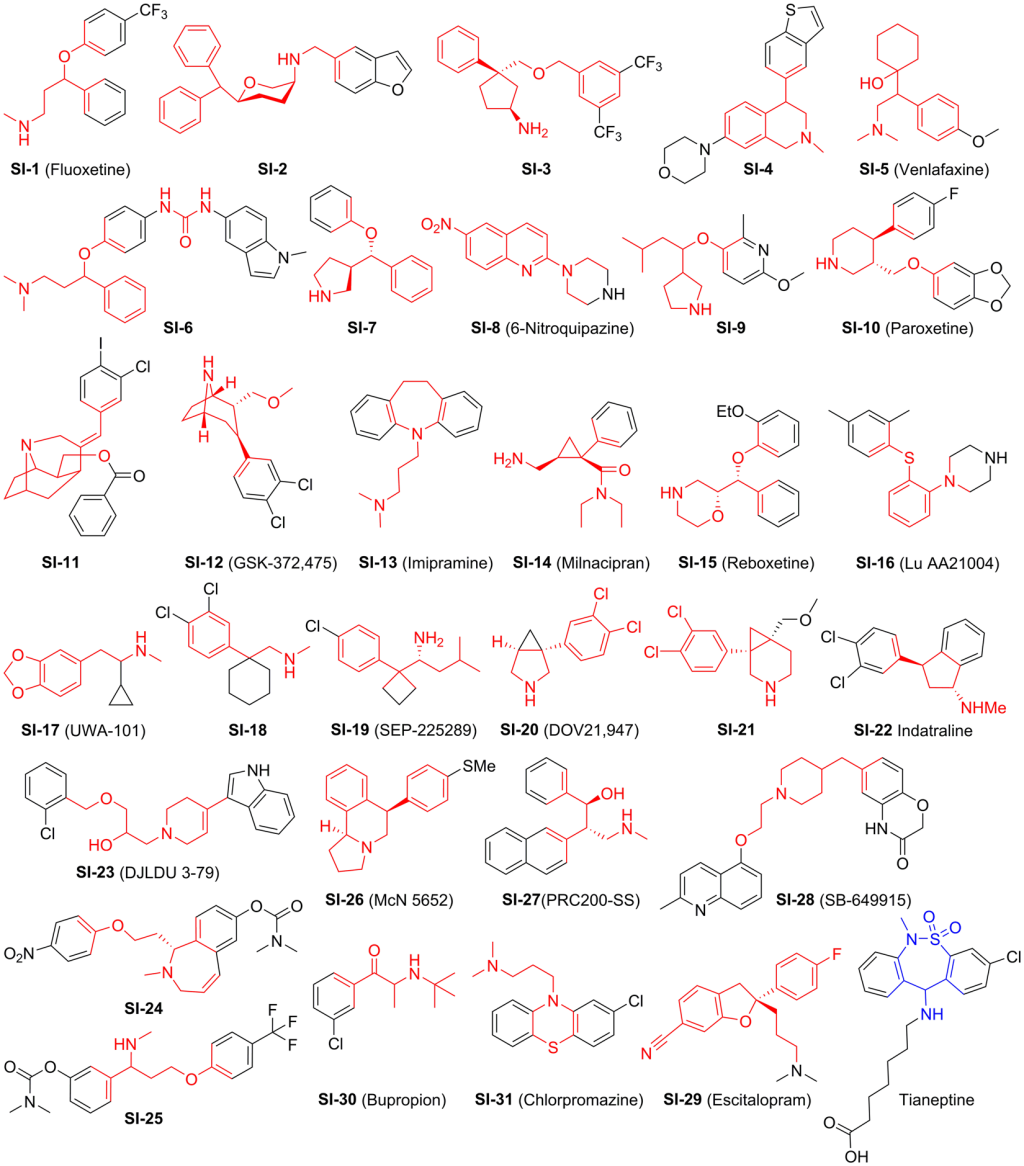
Molecular scaffolds of reported or marketed antidepressants targeting SERT.

**Figure 5:**
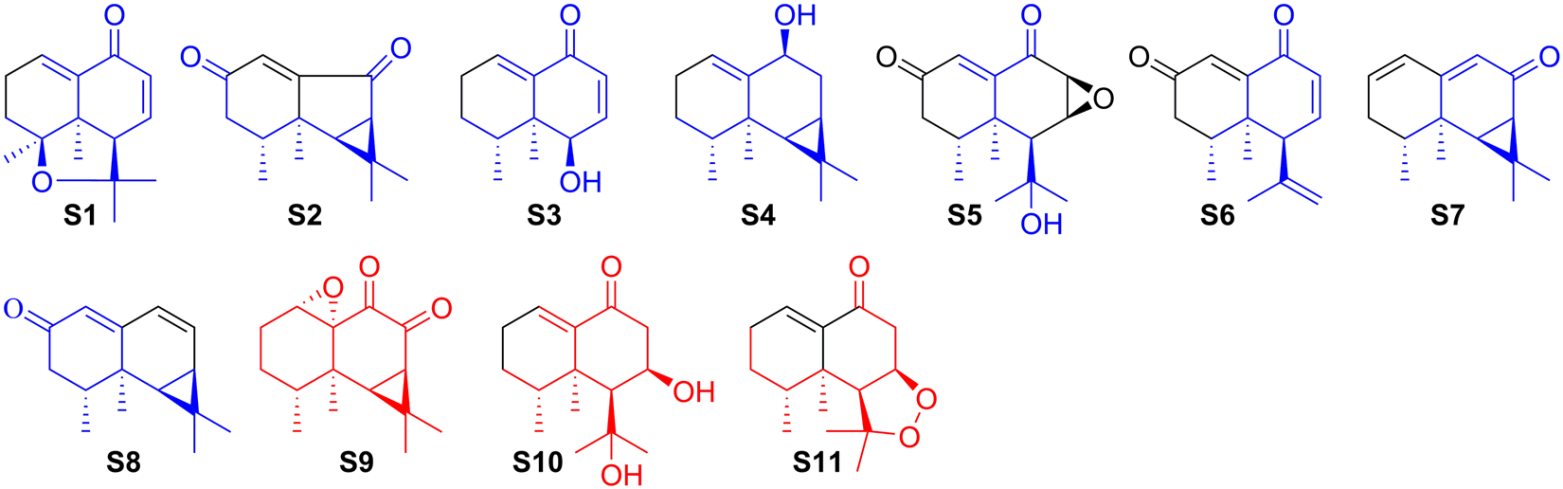
Potential natural sesquiterpenoid scaffolds targeting SERT from *N. chinensis* Batal.

Reported or marketed clinical antidepressants of the SERT mechanism, as known, include SERT inhibitors as well as SERT enhancers. SERT inhibitors (SIs) are dominant containing tricyclic antidepressants, selective serotonin reuptake inhibitors and selective noradrenaline reuptake inhibitors^29^, while the selective enhancer of SERT is rare and the only one in clinical use is tianeptine^30^. So far, hundreds of known SIs^31–52^, including 3-(aminoalkyl)-5-fluoroindole^53^, have been developed by chemical modification or hybridization of the known clinical antidepressants, based on the molecular templates shown in Fig. 4. Although the molecular structures of known SIs are diverse, they usually contain heterochains and/or heterocycles of N, O, and/or S, with halogen-substituted phenyls (F or Cl) as the common substituents. In spite of this, few SIs of natural products have been discovered, the only example, cyclo(L-Phe-L-Phe) found in chicken essence, is a dual inhibitor of SERT and acetylcholinesterase^54^. As far as we know, this is the first report on SERT activities of the natural aristolane-type and nardosinane-type sesquiterpenoids. Further, according to the above structure-activity discussion of known SIs, it suggests that eleven compounds (Fig. 5) including eight SERT enhancers (nardonoxide, nardoaristolone B, desoxo-nachinol A, 1(10)-aristolen-9*β*-ol, kanshone E, nardosinanone H, kanshone H, nardostachone, **S1**–**S8**) and three SERT inhibitors (kanshone C, nardosinonediol, nardosinone, **S9**–**S11**) may provide novel potential scaffolds for synthesis of SERT regulators.

In conclusion, forty sesquiterpenoids were isolated from roots and rhizomes of *Nardostachys chinensis* Batal., and their structures were identified by combined modern spectroscopic methods. Among these compounds, eleven natural scaffolds bidirectionally regulate SERT activity. They are potential lead compounds for regulation of SERT activity in drug discovery and provide novel molecular templates for synthesis of SERT enhancers and inhibitors, especially enhancer of SERT, which is rare so far in drug discovery. Presently, we are exploiting more SERT regulation lead structures from NCB and investigating antidepressant effect of SERT regulators *in vivo* assays to be reported in due time.

## Methods

### General experimental procedures

Optical rotations were measured using a Rudolph AUTOPOL V polarimeter (Rudolph Research Analytical, Hackettstown, USA). UV and electronic circular dichroism spectra were obtained on a Jasco J-815 circular dichroism spectropolarimeter (JASCO Corporation, Tokyo, Japan). ESIMS was performed on a Waters Synapt G2 mass spectrometer (Waters, Milford, MA, USA), and HRESIMS spectra was obtained on a quadrupole time-of-flight mass spectrometer QSTAR^TM^ Elite system (Applied Biosystems/MDS Sciex, Foster, CA, USA; Concord, ON, Canada). 1D and 2D NMR spectra were recorded on a Bruker AV-III spectrometer (^1^H/^13^C, 400 MHz/100 MHz, 600 MHz/150 MHz, Bruker, Zurich, Switzerland) using TMS as an internal standard. Chemical shifts (*δ*) were expressed in ppm. X-ray crystal data were analyzed on Rigaku MicroMax-007HF (Rigaku Corporation, Japan), preformed with Mo K*α*. Preparative HPLC was performed on Waters 2489 HPLC system (Waters, Milford, MA) using an Agilent Zorbax SB-C18 ODS column (21.2 mm × 250 mm, 7 *μ*m). Column chromatography (CC) was carried out with silica gel (200–300/400–500 mesh, Qingdao Marine Chemical, Inc., Qingdao, China), sephadex LH-20 (GE Healthcare UK Ltd, Buckinghamshire, England) and D101 macroporous resin (Tianjin Chemical Co., Ltd., Tianjin, China). Column fractions were monitored by TLC (silica gel 60 GF254, 15 *µ*m, Merck, Darmstadt, Germany), and the spots were visualized by heating the plates after spraying with 10% H_2_SO_4_ in ethanol. All reagents of HPLC or analytical grade were purchased from Tianjin Damao Reagent Co., Ltd., Tianjin, China.

### Plant material

The air-dried plant materials were purchased from Anhui Jiren Pharmacy Co. Ltd. (Bozhou, China) in July, 2011, and were identified as roots and rhizomes of *Nardostachys Chinensis* Batal. by Prof. Tian-xiang Li, Tianjin University of Traditional Chinese Medicine. A voucher specimen (No. B20604126) was deposited in the Tianjin Key Laboratory of Modern Chinese Medicine, Institute of Traditional Chinese Medicine, Tianjin University of Traditional Chinese Medicine, Tianjin, China.

### Extraction and isolation

The air-dried roots and rhizomes (20.0 kg) of NCB were first percolated 3 times by 8 times amount of 70% ethanol (*v*/*v*) at a speed of 10 ml/min under room temperature, and the residues were then reflux extracted 3 times (2 hours each time) by 8 times amount of 70% ethanol at 80–90 °C. The combined 70% ethanol solution was concentrated to dryness *in vacuo* (3.4 kg) and resuspended in water before being partitioned successively with petroleum ether, ethyl acetate, and *n*-butanol to give petroleum ether (PE), ethyl acetate (EA), and n-butanol (BU) extracts. The BU extract, combined with the extract afforded by alcohol precipitation of the rest water solution, was chromatographed on D101 macroporous resin column, gradiently eluted with EtOH/H_2_O (0:100−95:5) to obtain 5 fractions (H_2_O, 1000 g; 30%, 190 g; 50%, 175 g; 70%, 140 g; 95%, 70 g). Among them, the 95% EtOH fraction was concentrated to dryness *in vacuo* before combined with the EA extract. The PE extract (320 g) was fractionated by silica gel CC, gradiently eluted with PE/EA (100:0–0:100) to obtain 22 primary fractions Fr.1–Fr.22. Fr.5 (120.0 g) was successively subjected to a sephadex LH-20 CC (CH_2_Cl_2_/MeOH, 1:1) and a silica gel CC (PE/EA, 90:10) to afford 6 subfractions Fr.5–1–5–6, among which, Fr.5–1 gave compound **30** (4 mg, *Rf* = 0.3, CH_2_Cl_2_:MeOH = 1:1) and Fr.5–5 gave compound **29** (8 mg, *Rf* = 0.3, PE:EA:MeOH = 7:2:1) after purifications by preparative thin layer chromatography (pTLC) over silica gel. Fr. 6 (57.0 g) and Fr. 9 (40 g) were then subjected to repeated silica gel CC (PE/EA, 100:0 to 70:30) to afford compounds **2** (13 mg), **4** (280 mg), **5** (10 mg), **16** (250 mg), **20** (240 mg), **26** (3 mg) and **36** (4 mg). One subfraction Fr.6–4–5 from Fr.6 was further subjected to semi-preparative HPLC (MeOH:H_2_O = 80:20) to give compound **13** (*t*_R_ = 7.0 min, 4.7 mg). Fr.7 (70 g) was separated by reverse phase ODS CC (MeOH/H_2_O, 65:35 to 100:0) to give 6 subfractions Fr.7–1–7–6. Fr.7–1 was then subjected to repeated silica gel CC (PE/EA, 100:0 to 70:30) to give compounds **1** (8 mg), **19** (6 mg) and **32** (8 mg). Similarly, Fr.7–2–7–4 were subjected to repeated silica gel CC (PE/EA, 100:0 to 70:30) to give compounds **23** (5 mg), **24** (6 mg) and **27** (3 mg). The remain subfractions from Fr.7–2–7–4 were further purified by silica gel pTLC eluted with PE/EA/methanol (9:0.5:0.5 for **3**, **7**, and **15**, 7:2:1 for **6**, 8:1.5:0.5 for **17**, 5:3:2 for **38**, and 2:1:2 for **35** and **40**) to afford compounds **3** (2 mg, *Rf* = 0.2), **6** (5 mg, *Rf* = 0.3), **7** (6.5 mg, *Rf* = 0.5), **15** (9 mg, *Rf* = 0.5), **17** (5 mg, *Rf* = 0.6), **38** (8 mg, *Rf* = 0.5), **35** (4 mg, *Rf* = 0.2) and **40** (3 mg, *Rf* = 0.3). Compounds **8** (15 mg) and **9** (12 mg) were obtained by recrystallization of the subfraction Fr.7–2–2–1, and compound **10** (3 mg) was given by recrystallization of the subfraction Fr.7–2–2–9. Fr.10 (35 g) was successively subjected to a sephadex LH-20 CC (CH_2_Cl_2_/MeOH, 1:1) and a silica gel CC (PE/EA, 70:30) to afford **28** (7 mg). Fr.13 (54 g) was fractionated by silica gel CC (CH_2_Cl_2_/MeOH, 90:10) to subfrations Fr.13–1–Fr.13–6. Fr.13–2 was then subjected to silica gel pTLC to give compound **25** (4 mg, *Rf* = 0.3, PE:EA:MeOH = 7:2:1). Fr. 15 (7.0 g) was subjected to silica gel CC (PE/EA, 100:0 to 0:100) to obtain 18 primary fractions Fr.15–1–Fr.15–18. Subfraction Fr.15–1 was then subjected to semi-preparative HPLC (MeOH:H_2_O = 60:40) to afford compound **37** (*t*_R_ = 10.0 min, 2.5 mg). Fr. 17 (3.0 g) was then subjected to silica gel CC (PE/EA, 100:0 to 0:100) to obtain 16 sufractions Fr.17–1–Fr.17–16. Subfraction Fr.17–9 was further isolated by semi-preparative HPLC (MeOH:H_2_O = 65:35) to yield compound **34** (*t*_R_ = 5.0 min, 4.2 mg).

The EA extract (1200 g) was fractionated by silica gel CC, gradiently eluted with CH_2_Cl_2_/MeOH (100:0–0:100) to obtain 15 primary fractions Fr.1–Fr.15. Fr.10 (40 g) was subjected to silica gel CC eluted with 100% EA to get 8 subfratcions Fr.10–1–Fr.10–8. And then, subfraction Fr.10–3 was successively subjected to a sephadex LH-20 CC (100% MeOH) and a silica gel CC (CH_2_Cl_2_/MeOH, 100:0 to 0:100) to give compound **21** (17.5 mg). Fr.1 (782 g) was separated into 12 subfractions Fr.1–1–Fr.1–12 by normal-phase silica gel CC (PE/EA, 100:0 to 0:100). Subfraction Fr.1–1 (25 g) was then subjected to repeated silica gel CC to give 15 fractions Fr.1–1–1–Fr.1–1–15. Fraction Fr.1–1–5 was fractionated to 6 subfractions Fr.1–1–5–1–Fr.1–1–5–5 by preparative HPLC (MeOH/H_2_O, 70:30 to 95:5, 70.0 min), and then subfraction Fr.1–1–5–1 was further purified by preparative HPLC (MeCN:H_2_O = 35:65) to yield compound **31** (*t*_R_ = 23.0 min, 11.0 mg). Similarly, subfraction Fr.4–2–3–2 afforded by repeated silica gel CC of Fr.4, was subjected to semi-preparative HPLC (MeOH:H_2_O = 65:35) to yield compound **12** (*t*_R_ = 56.3 min, 10 mg). Fr.1–5 (16 g) was isolated by preparative HPLC (MeOH/H_2_O, 30:70 to 95:5, 80.0 min) to afford 6 fractions Fr.1–5–1–Fr.1–5–6, among which fracion Fr.1–5–5 was purified to yield compound **18** (*t*_R = _37.5 min, 15.6 mg). Fr.1–9 (18 g) was separated into 10 fractions Fr.1–9–1–Fr.1–9–10 by preparative HPLC (MeOH/H_2_O, 30:70 to 95:5, 80.0 min), and fraction Fr.1–9–6 was further isolated by preparative HPLC (MeCN:H_2_O = 45:55) to yield compound **11** (*t*_R_ = 24.5 min, 11.3 mg). Subfraction Fr.1–2 (80 g) was chromatographed on silica gel CC eluted with PE/EA (from 50:1 to 0:100) to obtain 11 fractions Fr.1–2–1–Fr.1–2–11. Fraction Fr.1–2–11 was then purified to yield compound **14** (*t*_R_ = 51.0 min, 10.6 mg) by preparative HPLC (MeOH/H_2_O, 30:70 to 95:5, 65 min).

The 30% aqueous ethanol fraction (190 g) from the BU extract (1800 g) was then subjected to D101 macroporous resin column, gradiently eluted with MeOH/H_2_O (10:90–100:0) to obtain 5 subfractions (H_2_O, 39 g; 10%, 20 g; 20%, 17 g; 30%, 21 g; 100%, 70 g). The 10% MeOH subfraction was subjected to repeated sephadex LH-20 CC (50% aqueous MeOH or 100% MeOH) to afford compounds **22** (11.8 mg) and **33** (11.0 mg), and the rest samples were further isolated by preparative HPLC (MeOH:H_2_O = 28:72) to afford compound **39** (*t*_R_ = 16.1 min, 43.1 mg).

### Chemical structure data

The NMR spectra of the new compounds are provided in the Supplementary Information.

#### Nardoaristol (**3**)

Colorless oil (EtOAc); [*α*]20 D =  +18.00 (*c* 0.1, MeOH); UV (MeOH) *λ*_max_ 197.5; CD (*c* 0.05, MeOH) *λ*(Δ*ε*) 209.5 (−0.03), 215.5 (+0.012), 219 (−0.005), 221.5 (+0.02); ^1^H NMR (CDCl_3_, 400.13 MHz) and ^13^C NMR data (CDCl_3_, 100.61 MHz), see Supplementary Table S1 and Table 1; (−)-ESIMS *m/z* 235.19 [M−H]^−^, ( + )-ESIMS *m/z* 237.27 [M + H]^+^.

#### 3-Hydroxylkanshone H (**6**)

Colorless oil (EtOAc); [*α*]20 D = −50.13 (*c* 0.5, MeOH); UV (MeOH) *λ*_max_ 283.0; CD (*c* 0.05, MeOH) *λ*(Δ*ε*) 213.5 (+1.32), 275.5 (+0.08), 296.0 (+0.66), 336.5 (−3.55); ^1^H NMR (CDCl_3_, 400.13 MHz) and ^13^C NMR data (CDCl_3_, 100.61 MHz), see Supplementary Table S1 and Table 1; (−)-HRESIMS *m*/*z* 231.1385 [M−H]^−^ (calcd for C_15_H_19_O_2_^−^, 231.1391); (−)-ESIMS *m/z* 231.30 [M−H]^−^, (+)-ESIMS *m/z* 255.28 [M + Na]^+^.

#### 3-Oxokanshone H (**7**)

White amorphous powder (CH_2_Cl_2_); [*α*]20 D = −391.88 (*c* 0.32, MeOH); UV (MeOH) *λ*_max_ 198, 290; CD (*c* 0.05, MeOH) *λ*(Δ*ε*) 197 (−1.11), 221 (9.40), 307 (−3.12), 348 (−2.24); ^1^H NMR (CDCl_3_, 400.13 MHz) and ^13^C NMR data (CDCl_3_, 100.61 MHz), see Supplementary Table S2 and Table 1; (+)-HRESIMS *m*/*z* 231.1378 [M + H]^+^ (calcd for C_15_H_19_O_2_^+^, 231.1380); (−)-ESIMS *m/z* 229.97 [M−H]^−^, (+)-ESIMS *m/z* 231.33 [M + H]^+^.

#### 1-Hydroxylaristolone (**11**)

Colorless needles (MeOH); [*α*]20 D = −186.31 (*c* 0.52, CHCl_3_); UV (MeOH) *λ*_max_ 195, 230; CD (*c* 0.06, MeOH) *λ*(Δ*ε*) 201 (+3.20), 257 (−4.74), 294 (−0.71), 328 (−2.23); (−)-HRESIMS *m/z* 233.1544 [M−H]^−^ (calcd for C_15_H_21_O_2_^−^, 233.1542)., (+)-HRESIMS *m*/*z* 235.1670 [M + H]^+^ (calcd for C_15_H_23_O_2_^+^, 235.1693).

#### 9β-Debilon (**14**)

Colorless crystals (CH_2_Cl_2_); [*α*]20 D = −33.31 (*c* 0.73, MeOH); UV (MeOH) *λ*_max_ 198, 290; CD (*c* 0.1, MeOH) *λ*(Δ*ε*) 200 (−1.85), 218.9 (−0.09), 243.2 (−0.83), 260.8 (−0.45), 318.5 (−0.51), 379.4 (+0.07); ^1^H NMR (CDCl_3_, 400.13 MHz) and ^13^C NMR data (CDCl_3_, 100.62 MHz), Supplementary Table S2 and Table 1; (−)-HRESIMS *m/z* 251.1646 [M + OH]^−^ (calcd for C_15_H_23_O_3_^−^, 251.1647). (+)-HRESIMS *m*/*z* 257.1498 [M + Na]^+^ (calcd for C_15_H_22_O_2_Na^+^, 257.1512).

#### Aristolanhydride (**18**)

White amorphous powder (CH_2_Cl_2_); [*α*]20 D = −67.00 (*c* 0.32, MeOH); UV (MeOH) *λ*_max_ 210, 285; CD (*c* 0.07, MeOH) *λ*(Δ*ε*) 200 (−1.44), 209 (−0.62), 224 (−2.15); ^1^H NMR (CD_3_OD, 400.13 MHz) and ^13^C NMR data (CD_3_OD, 100.62 MHz), see Supplementary Table S2 and Table 1; (+)-HRESIMS *m*/*z* 249.1469 [M + H]^+^ (calcd for C_15_H_21_O_3_^+^, 249.1491); (−)-HRESIMS *m/z* 265.1441 [M + OH]^−^ (calcd for C_15_H_21_O_4_^−^, 265.1449).

#### Epoxynardosinone (**19**)

Colorless crystals (EtOAc); [*α*]20 D = +1.27 (*c* 1.0, MeOH); UV (MeOH) *λ*_max_ 197.0; ^1^H NMR (CDCl_3_, 400.13 MHz) and ^13^C NMR data (CDCl_3_, 100.61 MHz), see Supplementary Table S3 and Table 1; (−)-HRESIMS *m*/*z* 265.1453 [M - H]^−^ (calcd for C_15_H_21_O_4_^−^, 265.1445); (−)-ESIMS *m/z* 265.25 [M−H]^−^, ( + )-ESIMS *m/z* 267.33 [M + H]^+^.

#### Nardosinonetriol (**22**)

Colorless needles (MeOH); [*α*]20 D =  +58.89 (*c* 0.3, MeOH); UV (MeOH) *λ*_max_ 193, 243; CD (*c* 0.05, MeOH) *λ*(*Δ*ε): 208 (−1.57), 241 (+1.76), 284 (−2.29), 303 (−2.18), 335 (−2.27); ^1^H NMR (DMSO-*d*_6_, 600.23 MHz) and ^13^C NMR data (DMSO-*d*_6_, 150.94 MHz), see Supplementary Table S3 and Table 1; (−)-HRESIMS *m*/*z* 313.1649 [M + COOH]^−^ (calcd for C_16_H_25_O_6_^−^, 313.1657), (+)-HRESIMS *m*/*z* 291.1569 [M + Na]^+^ (cald for C_15_H_24_O_4_Na^+^, 291.1567).

#### 7-Oxonardosinone (**23**)

Colorless oil (EtOAc); [*α*]20 D = +4.67 (*c* 0.1, MeOH); UV (MeOH) *λ*_max_ 198.0, 295.5; CD (*c* 0.05, MeOH) *λ*(*Δ*ε): 203.0 (−0.67), 209 (−0.78), 225.0 (−0.14), 245.0 (−0.39), 293.5 (+1.57), 329.0 (−1.51); ^1^H NMR (CDCl_3_, 600.25 MHz) and ^13^C NMR data (CDCl_3_, 150.95 MHz), see Supplementary Table S3 and Table 1; (+)-HRESIMS *m*/*z* 251.1637 [M + H]^+^ (calcd for C_15_H_23_O_3_^+^, 251.1642); (−)-ESI-MS *m/z* 249.25 [M−H]^−^, (+)-ESI-MS: *m/z* 251.37 [M + H]^+^.

#### 7-Oxonardosinoperoxide (**24**)

Colorless oil (EtOAc); [*α*]20 D = +55.33 (*c* 0.3, MeOH); UV (MeOH) *λ*_max_ 197.5, 269.5; CD (*c* 0.05, MeOH) *λ*(*Δ*ε): 279.5 (+1.13), 326.5 (−0.91); ^1^H NMR (DMSO-*d*_6_, 400.13 MHz) and ^13^C NMR data (DMSO-*d*_6_, 100.61 MHz), see Supplementary Table S3 and Table 1; ( + )-HRESIMS *m*/*z* 267.1589 [M + H]^+^ (calcd for C_15_H_23_O_4_^+^, 267.1591); (−)-ESI-MS *m/z* 265.19 [M−H]^−^, (+)-ESI-MS *m/z* 267.36 [M + H]^+^.

#### 2-Oxokanshone A (**26**)

Colorless oil (EtOAc); [*α*]20 D = −153.85 (*c* 0.13, MeOH); UV (MeOH) *λ*_max_ 205, 266; CD (*c* 0.05, MeOH) *λ*(*Δ*ε): 205 (+2.16), 221 (+4.40), 295 (−3.05), 334 (−0.25); ^1^H NMR (CDCl_3_, 400.13 MHz) and ^13^C NMR data (CDCl_3_, 100.61 MHz), see Supplementary Table S4 and Table 1; (+)-HRESIMS *m*/*z* 249.1485 [M + H]^+^ (calcd for C_15_H_21_O_3_^+^, 249.1485).

#### Epoxynardosinanone H (**30**)

Colorless oil (EtOAc); [*α*]20 D = −160.56 (*c* 0.24, MeOH); UV (MeOH) *λ*_max_ 198, 231; CD (*c* 0.05, MeOH) *λ*(*Δ*ε): 216 (+7.24), 252 (−8.05), 312 (+3.92); ^1^H NMR (CDCl_3_, 400.13 MHz) and ^13^C NMR data (CDCl_3_, 100.61 MHz), see Supplementary Table S4 and Table 1; (+)-HRESIMS *m*/*z* 233.1535 [M + H]^+^ (calcd for C_15_H_21_O_2_^+^, 233.1536); (+)-ESI-MS: *m/z* 233.47 [M + H]^+^.

### X-ray crystallographic analysis of 1

The structure were solved by SHELXS-97 and refined by full-matrix least-squares techniques using the SHELXL-97 program. Crystallographic data for the structure of **1** reported in this study has been deposited with the Cambridge Crystallographic Data Centre under the reference number CCDC 1058758. Copies of the data can be obtained, free of charge, on application to the Director, CCDC, 12 Union Road, Cambridge CB2 1EZ, UK (fax: +44-(0)1223–336033 or e-mail: deposit@ccdc.cam.ac.uk).

#### Crystal data for **1**

Colorless prism, C_14_H_18_O_3_, *M = *234.28, 0.20 × 0.18 × 0.12 mm^3^, monoclinic, space group *P*2_1_; *a = *9.6605(19) Å, *b = *6.9639(14) Å, *c = *9.7024(19) Å, *α* = *γ* = 90°, *β* = 108.44(3)°, *V = *619.2(2) Å^3^, *Z = *2, *D*_calcd_ = 1.257 g/cm^3^, *F*_000_ = 252. Mo K*α* radiation, *λ* = 0.71073 Å, T = 113(2) K, *μ* (Mo K*α*) = 0.087 mm^−1^. The final *R*_1_ was 0.0443 and *wR*_2_ was 0.0852.

### Computational methods

Relative configurations of **11** and **18** were deduced by analysis of their 1D/2D NMR data. Stochastic conformational searches were firstly conducted under MMFF94 force field to give one and three possible conformers for **11** and **18**, respectively. Conformers were then optimized and the frequencies were calculated by further time-dependent density functional theory (TDDFT) method to verify their stability. Using the conformers at the B3LYP/6–31 G(d) basis set level in methanol, sixty excitation states at the B3LYP/6–31 G(d) basis set level were calculated, and finally the calculation results were Boltzmann averaged to yield the depicted electronic circular dichroism (ECD) spectra of **11** and **18** with half bandwidths of 0.45 eV and 0.40 eV, respectively. All calculations were performed by Gaussian 09 program package (Version C.01).

### SERT activity assay

The SERT activity assay was based on the reported method^4,5^ with modifications. To evaluate SERT activity of these compounds, a validated stably transfected hSERT-HEK293 cell line were used in the high content assay. The fluorescent substrate 4-[4-(Dimethylamino)phenyl]-1-methyl pyridinium (APP^+^) was used to examine SERT activity and the fluorescent dye Hoechst 33342 stain cellular nuclei. The effects of test compounds on SERT function were calculated by the following equation: Relative fluorescent intensity (RFI) = (Intracellular APP^+^ fluorescent intensity _treatment_/Intracellular APP^+^ fluorescent intensity _control_). The positive control drugs in testing SERT function include SSRI fluoxetine 2.0 *μ*M and SSRE tianeptine 1.0 *μ*M. All test compounds were run in triplicates with three repeat times. The data from the SERT function assays were analyzed by using SPSS software (Version 11.5, IBM Company). The RFI values under different treatments were evaluated by one-way analysis of variance (ANOVA), followed by post hoc testing using Dunnett’s multiple comparisons tests. The results were expressed in Mean ± S.E.M.^55^. Some available compounds were re-purified to make sure their purities ≥95% (HPLC, wavelengths = 230, 254, 280, 360 nm) before bioactivity evaluation.

## Electronic supplementary material

Supplementary Information
