# Supplementary material for: Novel serotonin transporter regulators: Natural aristolane- and nardosinane- types of sesquiterpenoids from Nardostachys chinensis Batal
Source: Sci Rep. 2017 Nov 8;7:15114. doi: 10.1038/s41598-017-15483-6 (PMC5678126; doi:10.1038/s41598-017-15483-6)
Supplement: Supplementary file 1 — Supplementary Information [file 41598_2017_15483_MOESM1_ESM.pdf]

Supplementary Information

Novel serotonin transporter regulators: Natural aristolane- and nardosinane- types of sesquiterpenoids from *Nardostachys chinensis* Batal.

Ying-Peng Chen[1,†], Shu-Song Ying[1,†], Hong-Hong Zheng[1], Yan-Ting Liu[1], Zhong-Ping Wang[1], Hu Zhang[1], Xu Deng[1], Yi-Jing Wu[1], Xiu-Mei Gao[1], Tian-Xiang Li[2], Yan Zhu[1], Yan-Tong Xu[1,*], Hong-Hua Wu[1,*]

[1] Tianjin State Key Laboratory of Modern Chinese Medicine, Tianjin Key Laboratory of Chemistry and Analysis of Traditional Chinese Medicine, Institute of Traditional Chinese Medicine, Tianjin University of Traditional Chinese Medicine, Tianjin 300193, China

[2] Chinese Medicine Research Center, Tianjin University of Traditional Chinese Medicine, Tianjin 300193, China

* Correspondence and requests for materials should be addressed to Y.-T.X. (email: tonyxu2015@sina.com) or H.-H.W. (emails: wuhonghua2003@163.com, wuhonghua2011@tjutcm.edu.cn)

[†] these authors contributed equally to this work and should be considered co-first authors

Table of Contents **Page**

Figure S1. (-)-HRESIMS of nardoaristolone C **(1)** 5
Figure S2. ^{1}H NMR (600.23 MHz, $CDCl_3$) spectrum of nardoaristolone C (**1**) 5
Figure S3. ^{13}C NMR (150.93 MHz, $CDCl_3$) spectrum of nardoaristolone C (**1**) 6
Figure S4. HSQC spectrum of nardoaristolone C (**1**) 6
Figure S5. HMBC spectrum of nardoaristolone C (**1**) 7
Figure S6. NOESY spectrum of nardoaristolone C (**1**) 7
Figure S7. ^{1}H NMR (400.13 MHz, $CDCl_3$) spectrum of nardoaristol (**3**) 8
Figure S8. ^{13}C NMR (100.61 MHz, $CDCl_3$) spectrum of nardoaristol (**3**) 8
Figure S9. HSQC spectrum of nardoaristol (**3**) 9
Figure S10. HMBC spectrum of nardoaristol (**3**) 9
Figure S11. NOESY spectrum of nardoaristol (**3**) 10
Figure S12. (-)-HRESIMS of 3-hydroxylkanshone H (**6**) 10
Figure S13. ^{1}H NMR (400.13 MHz, $CDCl_3$) spectrum of 3-hydroxylkanshone H (**6**) 11
Figure S14. ^{13}C NMR (100.61 MHz, $CDCl_3$) spectrum of 3-hydroxylkanshone H (**6**) 11
Figure S15. HSQC spectrum of 3-hydroxylkanshone H (**6**) 12
Figure S16. HMBC spectrum of 3-hydroxylkanshone H (**6**) 12
Figure S17. NOESY spectrum of 3-hydroxylkanshone H (**6**) 13
Figure S18. (+)-HRESIMS of 3-oxokanshone H (**7**) 13
Figure S19. ^{1}H NMR (400.13 MHz, $CDCl_3$) spectrum of 3-oxokanshone H (**7**) 14
Figure S20. ^{13}C NMR (100.61 MHz, $CDCl_3$) spectrum of 3-oxokanshone H (**7**) 14
Figure S21. HSQC spectrum of 3-oxokanshone H (**7**) 15
Figure S22. HMBC spectrum of 3-oxokanshone H (**7**) 15
Figure S23. NOESY spectrum of 3-oxokanshone H (**7**) 16
Figure S24. HRESIMS of 9*β*-debilon (**14**) 16
Figure S25. ^{1}H NMR (400.13 MHz, $CDCl_3$) spectrum of 9*β*-debilon (**14**) 17
Figure S26. ^{13}C NMR (100.62 MHz, $CDCl_3$) spectrum of 9*β*-debilon (**14**) 17
Figure S27. HSQC spectrum of 9*β*-debilon (**14**) 18
Figure S28. HMBC spectrum of 9*β*-debilon (**14**) 18
Figure S29. HRESIMS of aristolanhydride (**18**) 19
Figure S30. ^{1}H NMR (400.13 MHz, CD_3OD) spectrum of aristolanhydride (**18**) 19
Figure S31. ^{13}C NMR (100.62 MHz, CD_3OD) spectrum of aristolanhydride (**18**) 20
Figure S32. HSQC spectrum of aristolanhydride (**18**) 20
Figure S33. HMBC spectrum of aristolanhydride (**18**) 21
Figure S34. NOESY spectrum of aristolanhydride (**18**) 21
Figure S35. (-)-HRESIMS of epoxynardosinone (**19**) 22
Figure S36. ^{1}H NMR (400.13 MHz, $CDCl_3$) spectrum of epoxynardosinone (**19**) 22
Figure S37. ^{13}C NMR (100.61 MHz, $CDCl_3$) spectrum of epoxynardosinone (**19**) 23
Figure S38. HSQC spectrum of epoxynardosinone (**19**) 23
Figure S39. HMBC spectrum of epoxynardosinone (**19**) 24
Figure S40. NOESY spectrum of epoxynardosinone (**19**) 24
Figure S41. HRESIMS of nardosinonetriol (**22**) 25
Figure S42. ^{1}H NMR (600.23 MHz, DMSO-d_6) spectrum of nardosinonetriol (**22**) 26
Figure S43. ^{13}C NMR (150.94 MHz, DMSO-d_6) spectrum of nardosinonetriol (**22**) 26

Figure S44. HSQC spectrum of nardosinonetriol (**22**) 27
Figure S45. HMBC spectrum of nardosinonetriol (**22**) 27
Figure S46. NOESY spectrum of nardosinonetriol (**22**) 28
Figure S47. (+)-HRESIMS of 7-oxonardosinone (**23**) 28
Figure S48. ^{1}H NMR (600.25 MHz, $CDCl_3$) spectrum of 7-oxonardosinone (**23**) 29
Figure S49. ^{13}C NMR (150.95 MHz, $CDCl_3$) spectrum of 7-oxonardosinone (**23**) 29
Figure S50. HSQC spectrum of 7-oxonardosinone (**23**) 30
Figure S51. HMBC spectrum of 7-oxonardosinone (**23**) 30
Figure S52. NOESY spectrum of 7-oxonardosinone (**23**) 31
Figure S53. (+)-HRESIMS of 7-oxonardosinoperoxide (**24**) 31
Figure S54. ^{1}H NMR (400.13 MHz, DMSO-d_6) spectrum of 7-oxonardosinoperoxide (**24**) 32
Figure S55. ^{13}C NMR (100.61 MHz, DMSO-d_6) spectrum of 7-oxonardosinoperoxide (**24**) 32
Figure S56. HSQC spectrum of 7-oxonardosinoperoxide (**24**) 33
Figure S57. HMBC spectrum of 7-oxonardosinoperoxide (**24**) 33
Figure S58. NOESY spectrum of 7-oxonardosinoperoxide (**24**) 34
Figure S59. (+)-HRESIMS of 2-oxokanshone A (**26**) 34
Figure S60. ^{1}H NMR (400.13 MHz, $CDCl_3$) spectrum of 2-oxokanshone A (**26**) 35
Figure S61. ^{13}C NMR (100.61 MHz, $CDCl_3$) spectrum of 2-oxokanshone A (**26**) 35
Figure S62. HSQC spectrum of 2-oxokanshone A (**26**) 36
Figure S63. HMBC spectrum of 2-oxokanshone A (**26**) 36
Figure S64. NOESY spectrum of 2-oxokanshone A (**26**) 37
Figure S65. (+)-HRESIMS of epoxynardosinanone H (**30**) 37
Figure S66. ^{1}H NMR (400.13 MHz, $CDCl_3$) spectrum of epoxynardosinanone H (**30**) 38
Figure S67. ^{13}C NMR (100.61 MHz, $CDCl_3$) spectrum of epoxynardosinanone H (**30**) 38
Figure S68. DEPT(135°) spectrum of epoxynardosinanone H (**30**) 39
Figure S69. HSQC spectrum of epoxynardosinanone H (**30**) 39
Figure S70. HMBC spectrum of epoxynardosinanone H (**30**) 40
Figure S71. (+)-HRESIMS of nardosinanone M (**35**) 40
Figure S72. ^{1}H NMR (400.13 MHz, $CDCl_3$) spectrum of nardosinanone M (**35**) 41
Figure S73. ^{13}C NMR (100.61 MHz, $CDCl_3$) spectrum of nardosinanone M (**35**) 41
Figure S74. HSQC spectrum of nardosinanone M (**35**) 42
Figure S75. HMBC spectrum of nardosinanone M (**35**) 42
Figure S76. NOESY spectrum of nardosinanone M (**35**) 43
Figure S77. UV and CD spectra of compounds **1**, **3**, **6**, **7**, **11**, **14**, **18**, **19**, **22**, **23**, **24**, **26**, **30** and **35** 44
Figure S78. Comparative UV spectra for compounds **1**– **40** 47
Figure S79. Comparative CD spectra for some aristolane- and nardosinane- types of sesquiterpenoids from NCB 50
Figure S80. Calculated and experimental UV and ECD spectra of compound **11** 54
Figure S81. Calculated and experimental UV and ECD spectra of compound **18** 55
Figure S82. Proposed biosynthetic pathway for aristolane-type sesquiterpenoids from NCB. 56
Figure S83. Proposed biosynthetic pathway for nardosinane-type sesquiterpenoids from NCB 57
Table S1. The optimized conformer of compound **11** 58
Table S2. The optimized conformers of compound **18** 58

Table S3. ^{1}H NMR (400.13 MHz, $CDCl_3$) data for aristolane-type sesquiterpenoids (1) 59
Table S4. ^{1}H NMR (400.13 MHz, $CDCl_3$) data for aristolane-type sesquiterpenoids (2) 59
Table S5. ^{1}H NMR (400.13 MHz, $CDCl_3$) data for nardosinone-type sesquiterpenoids (1) 60
Table S6. ^{1}H NMR (400.13 MHz, $CDCl_3$) data for nardosinone-type sesquiterpenoids (2) 60
Table S7. Effects of the compounds identified from NCB on SERT activity 61

Figure S1. (-)-HRESIMS of nardoaristolone C (**1**)

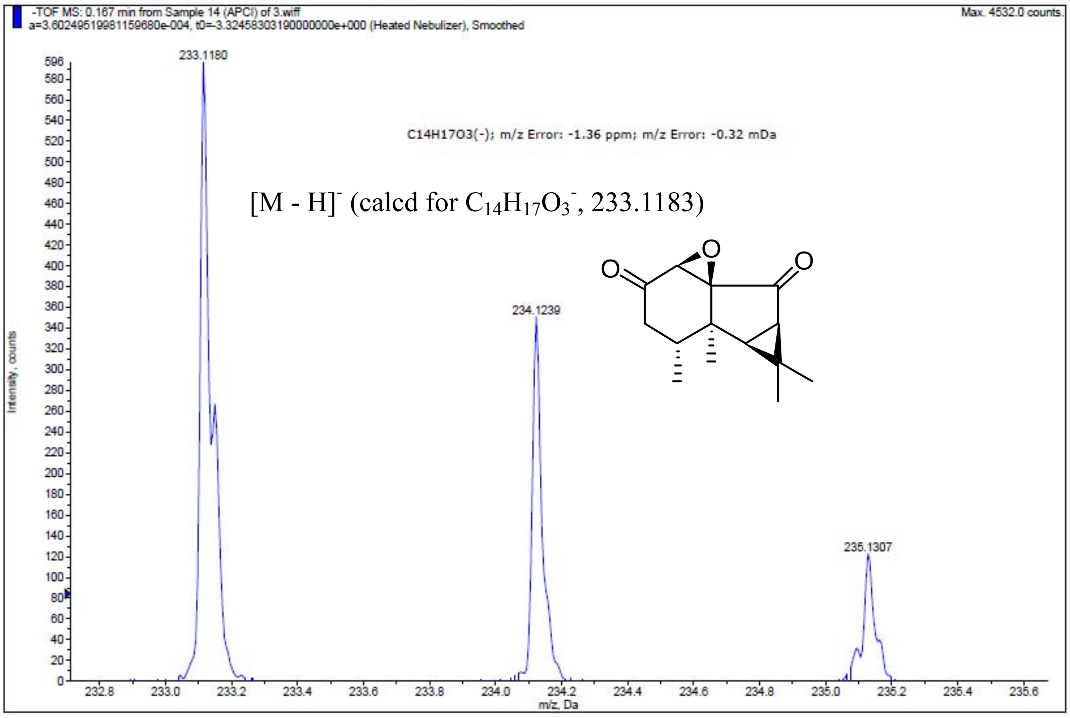

Figure S2. ^{1}H NMR (600.23 MHz, $CDCl_3$) spectrum of nardoaristolone C (**1**)

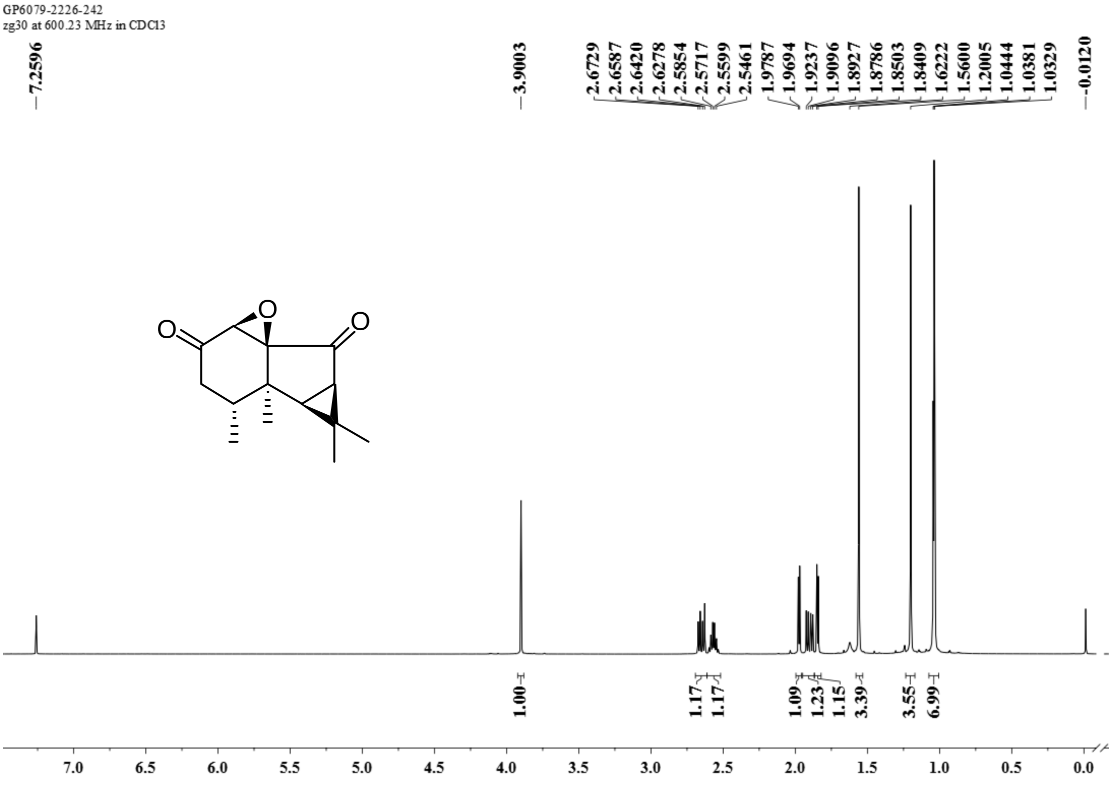

Figure S3. ^{13}C NMR (150.93 MHz, $CDCl_3$) spectrum of nardoaristolone C (**1**)

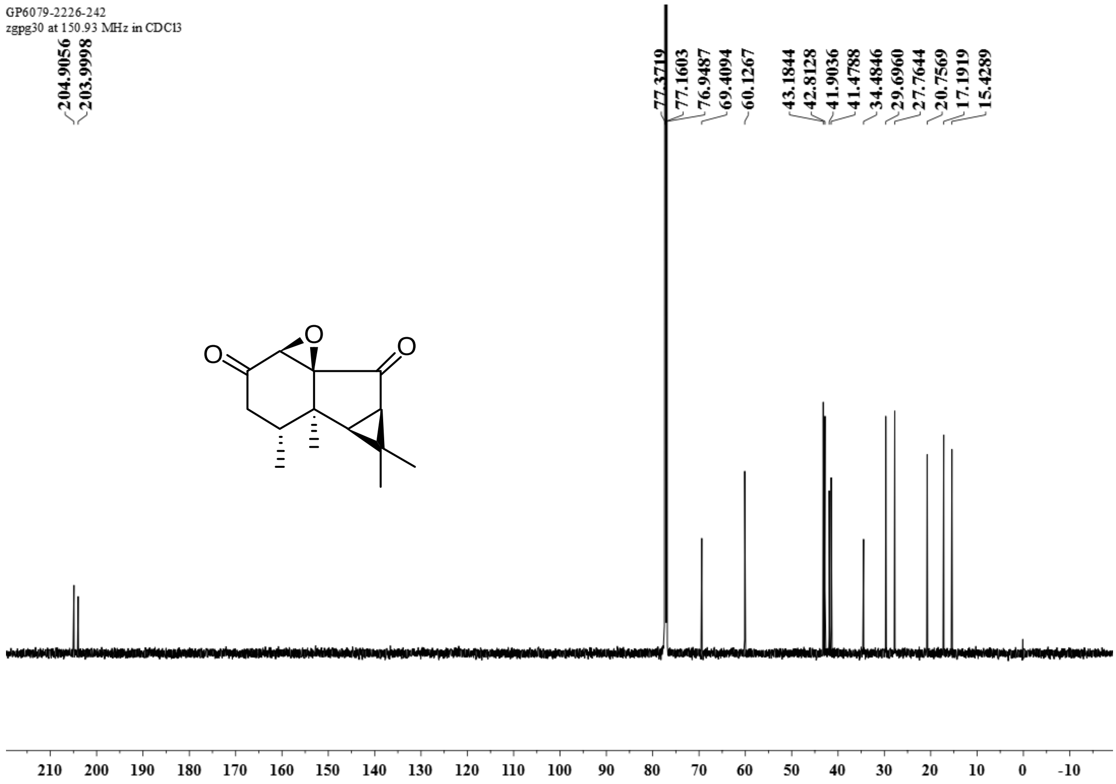

Figure S4. HSQC spectrum of nardoaristolone C (**1**)

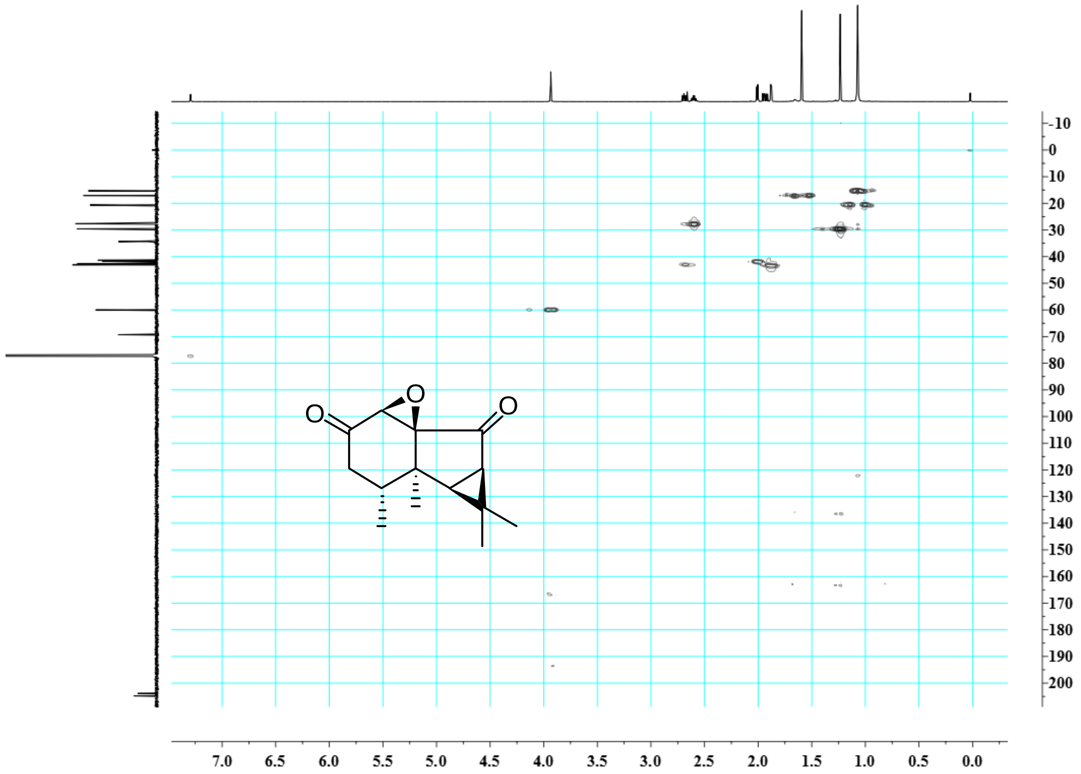

Figure S5. HMBC spectrum of nardoaristolone C (**1**)

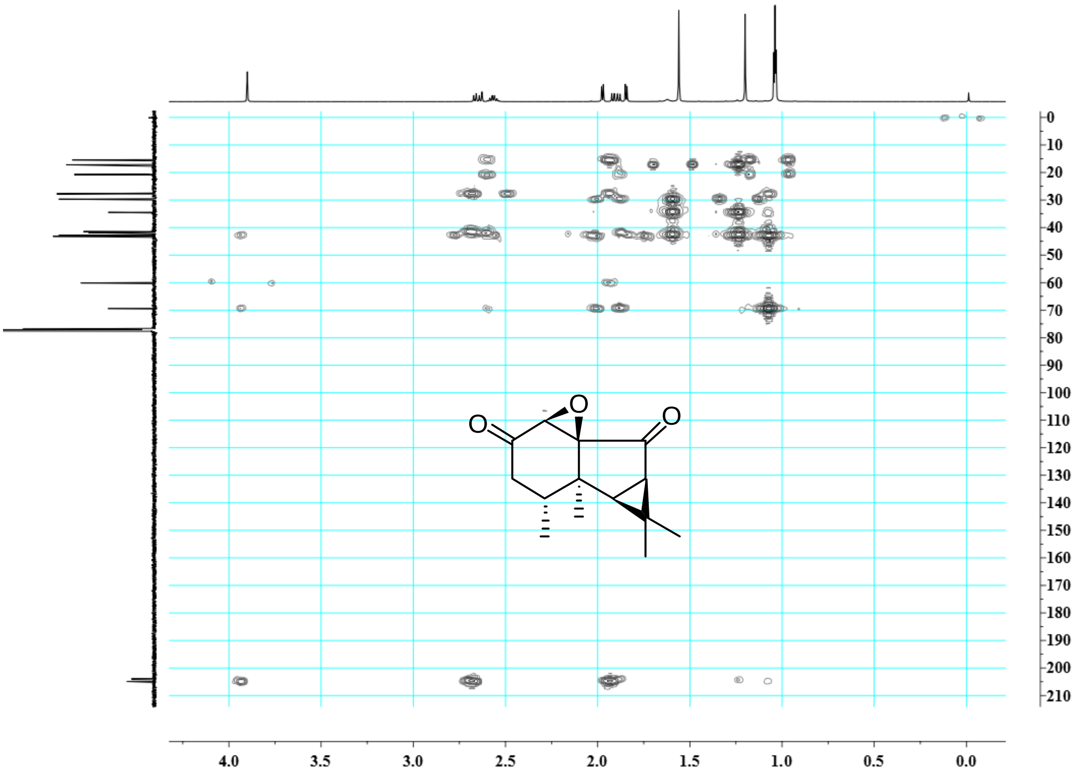

Figure S6. NOESY spectrum of nardoaristolone C (**1**)

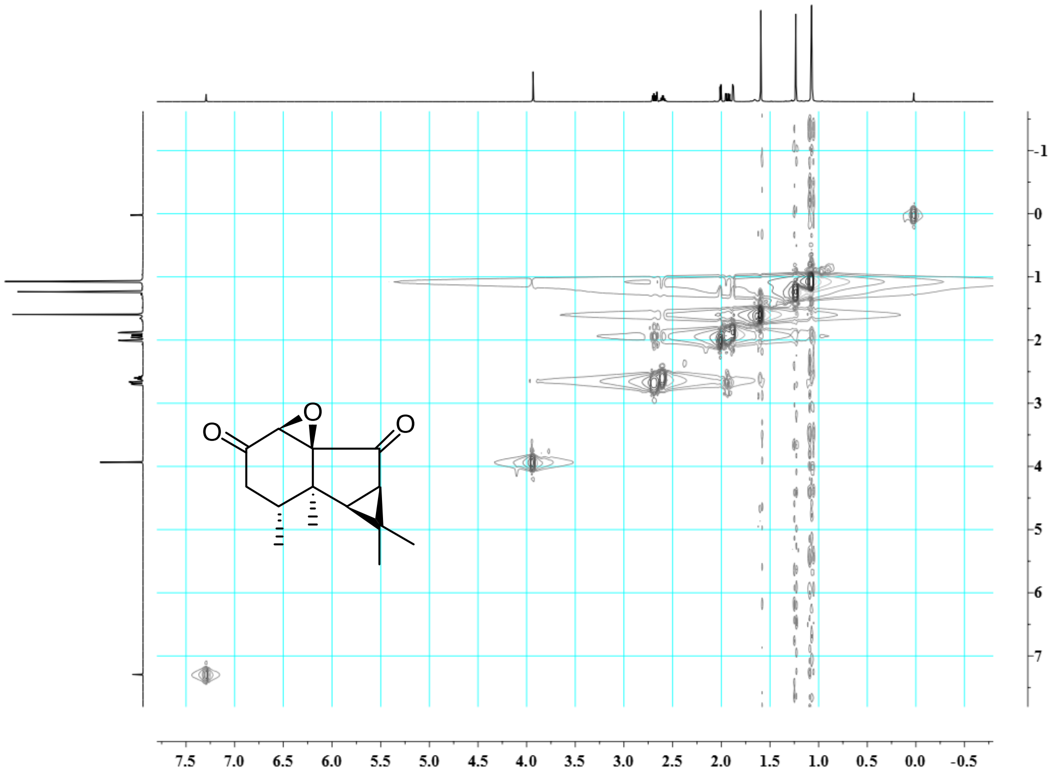

Figure S7. ^{1}H NMR (400.13 MHz, $CDCl_3$) spectrum of nardoaristol (**3**)

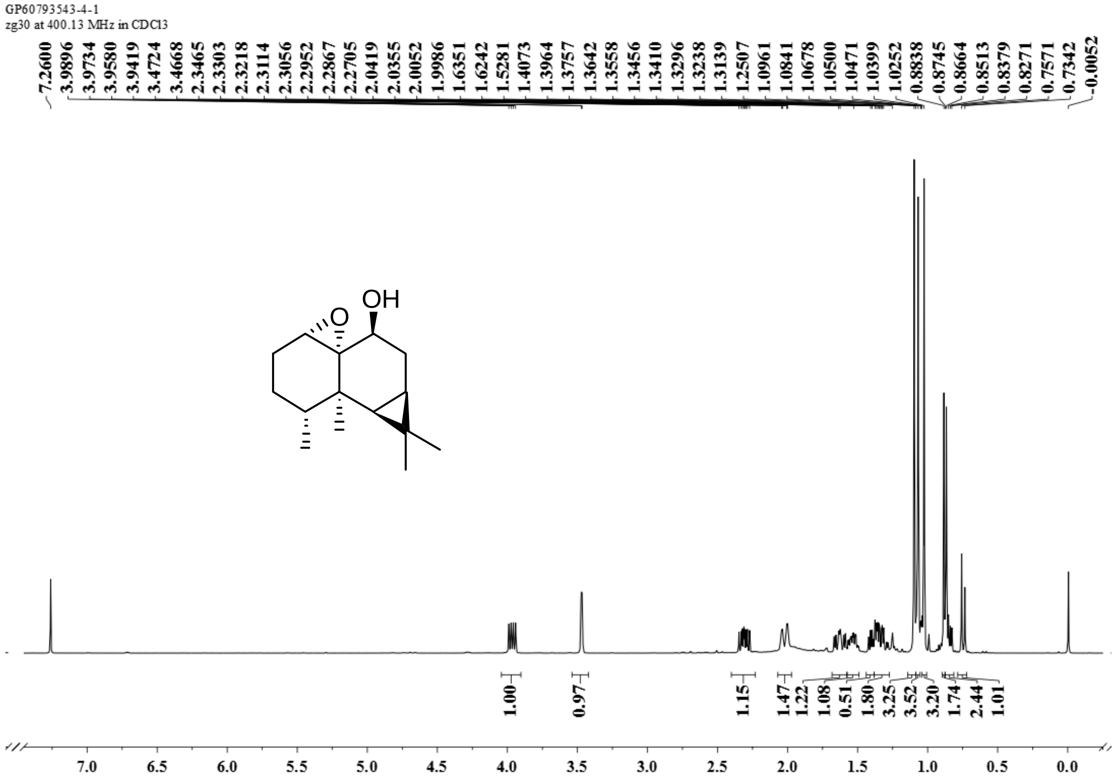

Figure S8. ^{13}C NMR (100.61 MHz, $CDCl_3$) spectrum of nardoaristol (**3**)

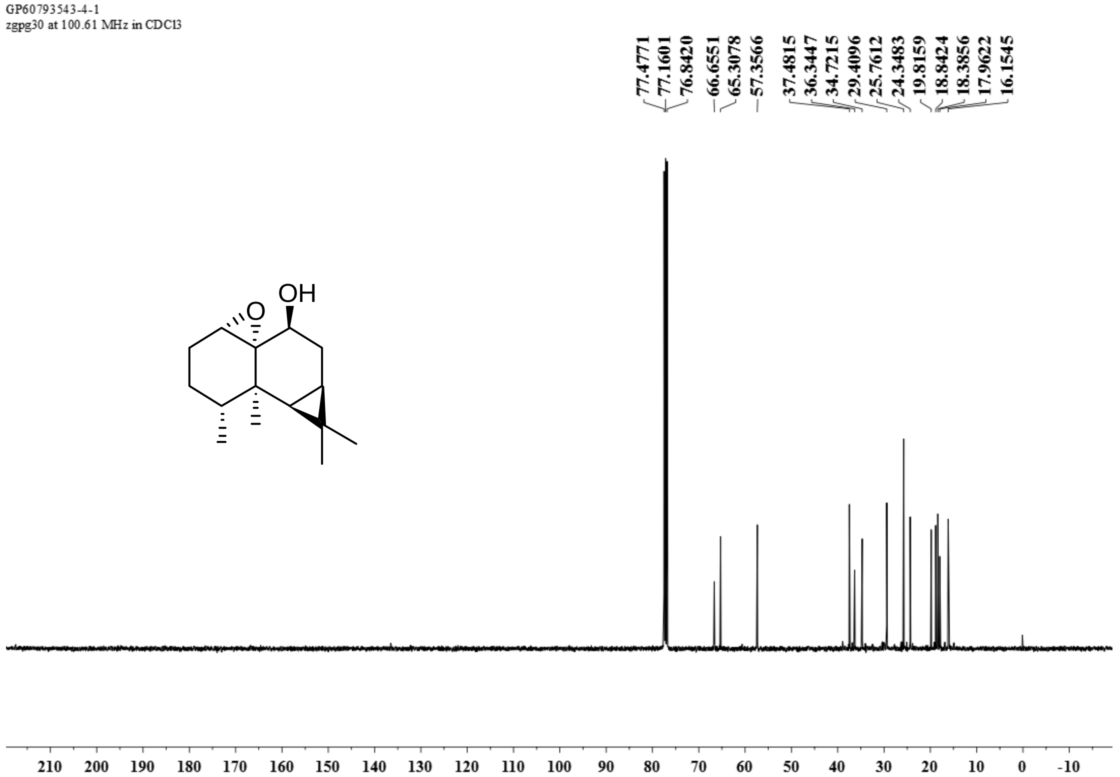

Figure S9. HSQC spectrum of nardoaristol (**3**)

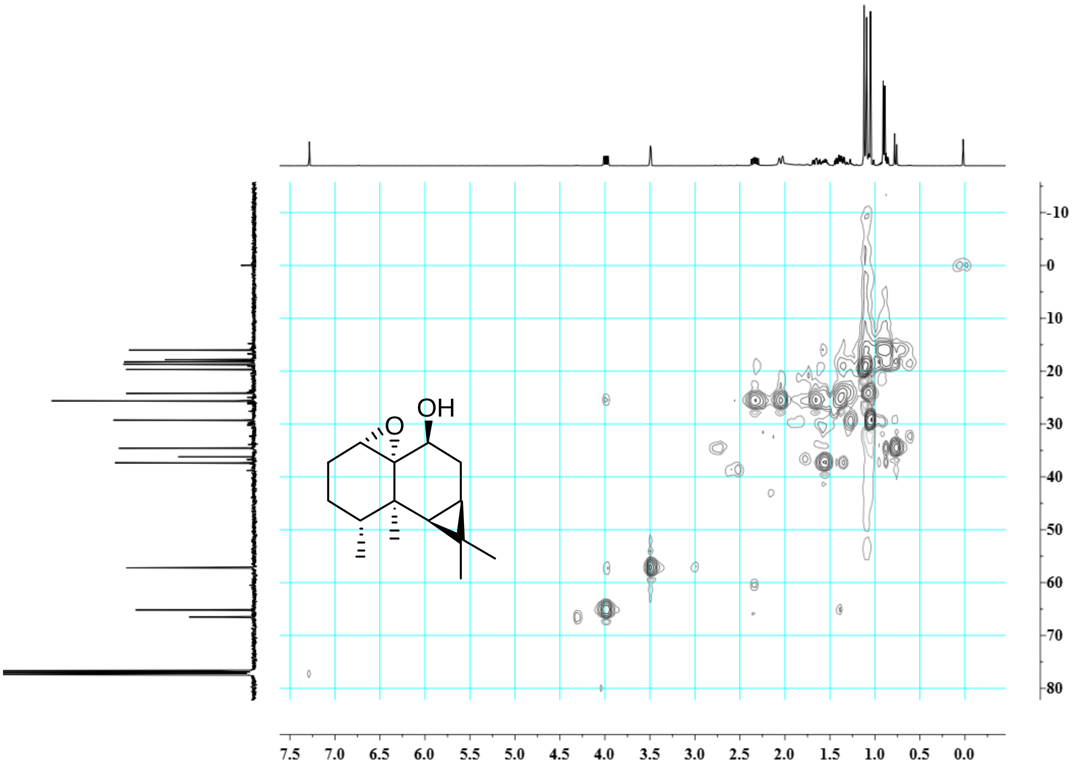

Figure S10. HMBC spectrum of nardoaristol (**3**)

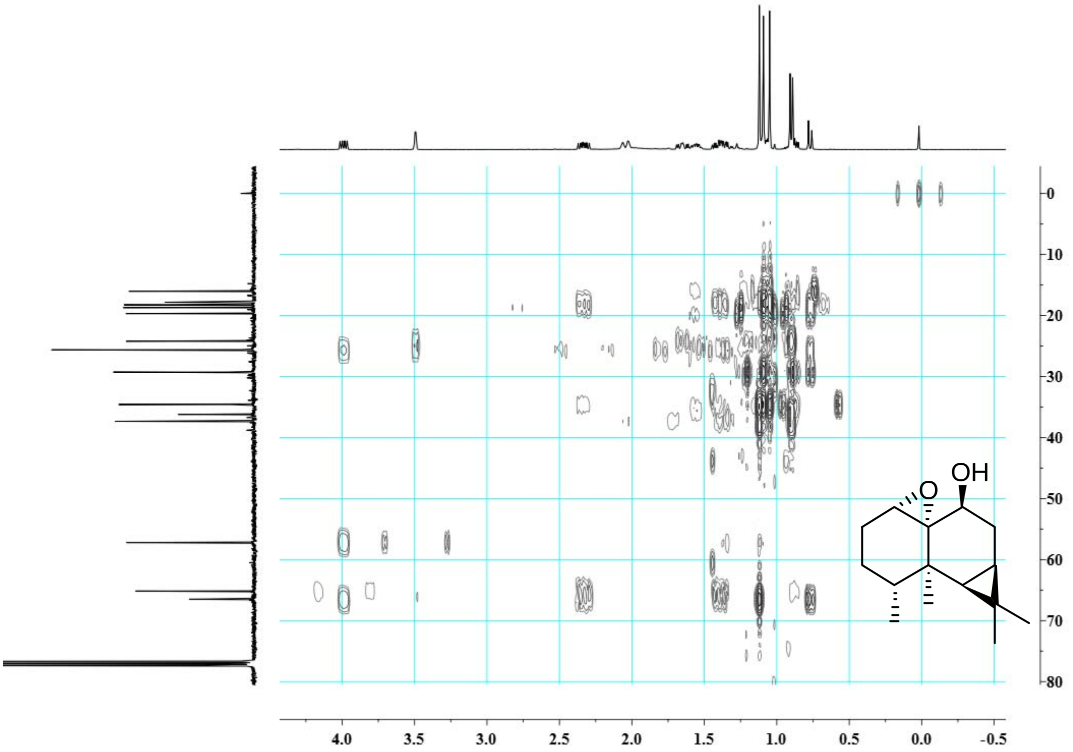

Figure S11. NOESY spectrum of nardoaristol (**3**)

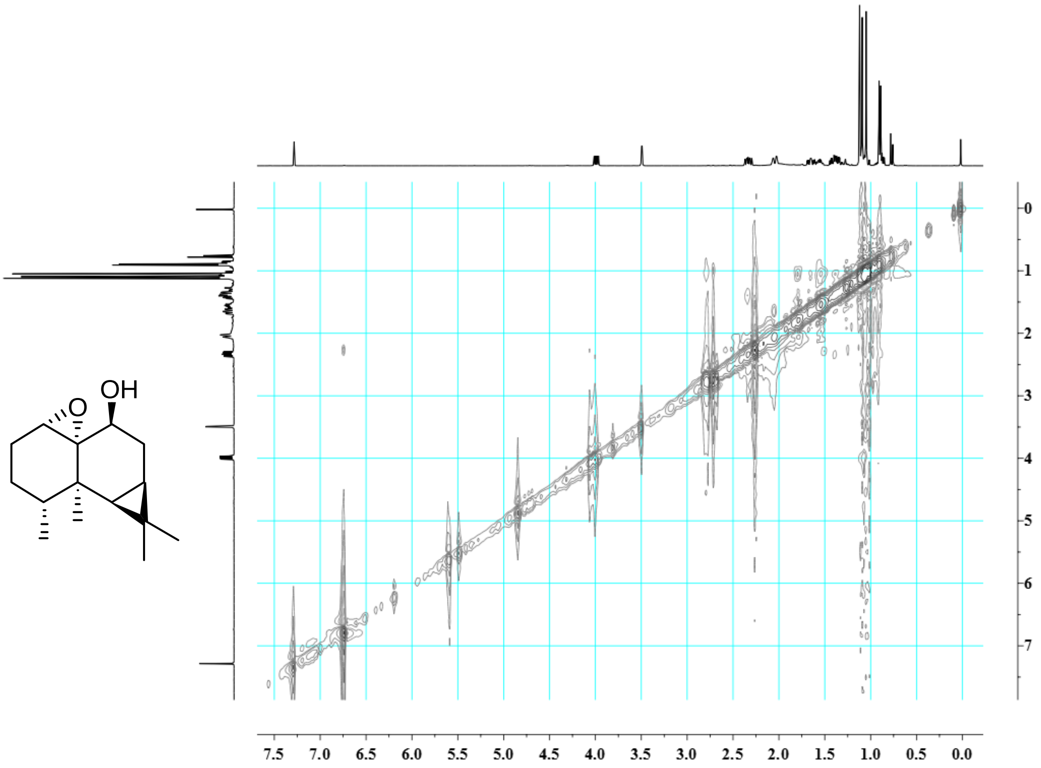

Figure S12. (-)-HRESIMS of 3-hydroxylkanshone H (**6**)

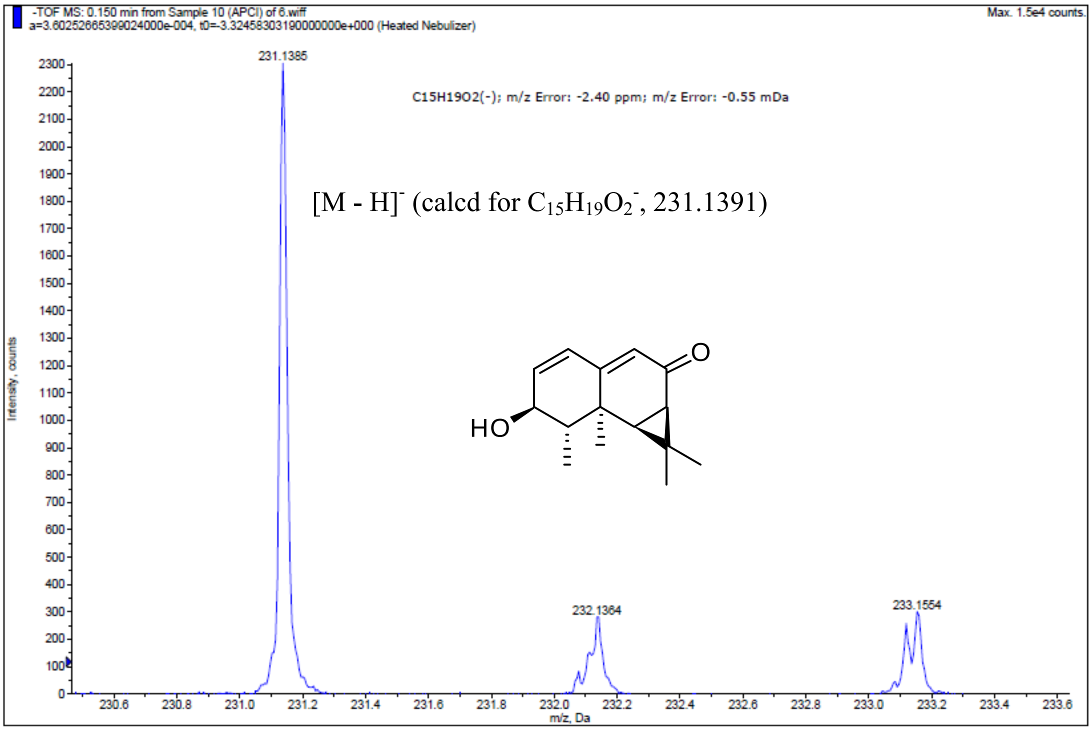

Figure S13. ^{1}H NMR (400.13 MHz, $CDCl_3$) spectrum of 3-hydroxylkanshone H (**6**)

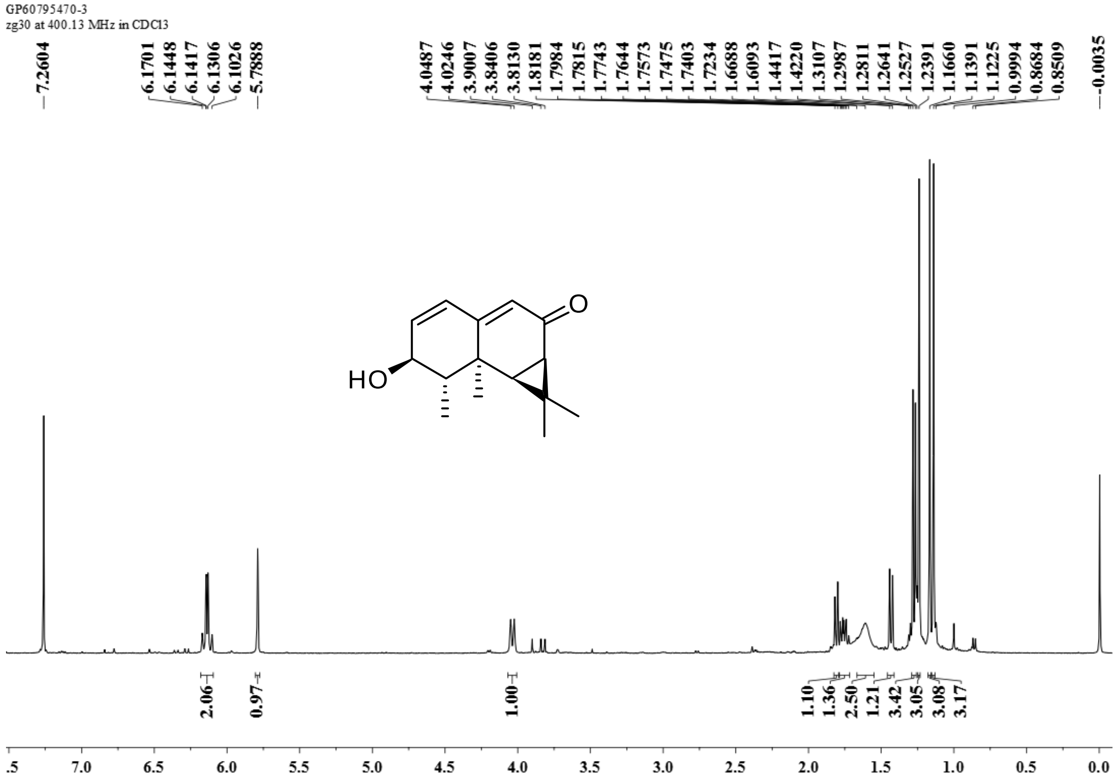

Figure S14. ^{13}C NMR (100.61 MHz, $CDCl_3$) spectrum of 3-hydroxylkanshone H (**6**)

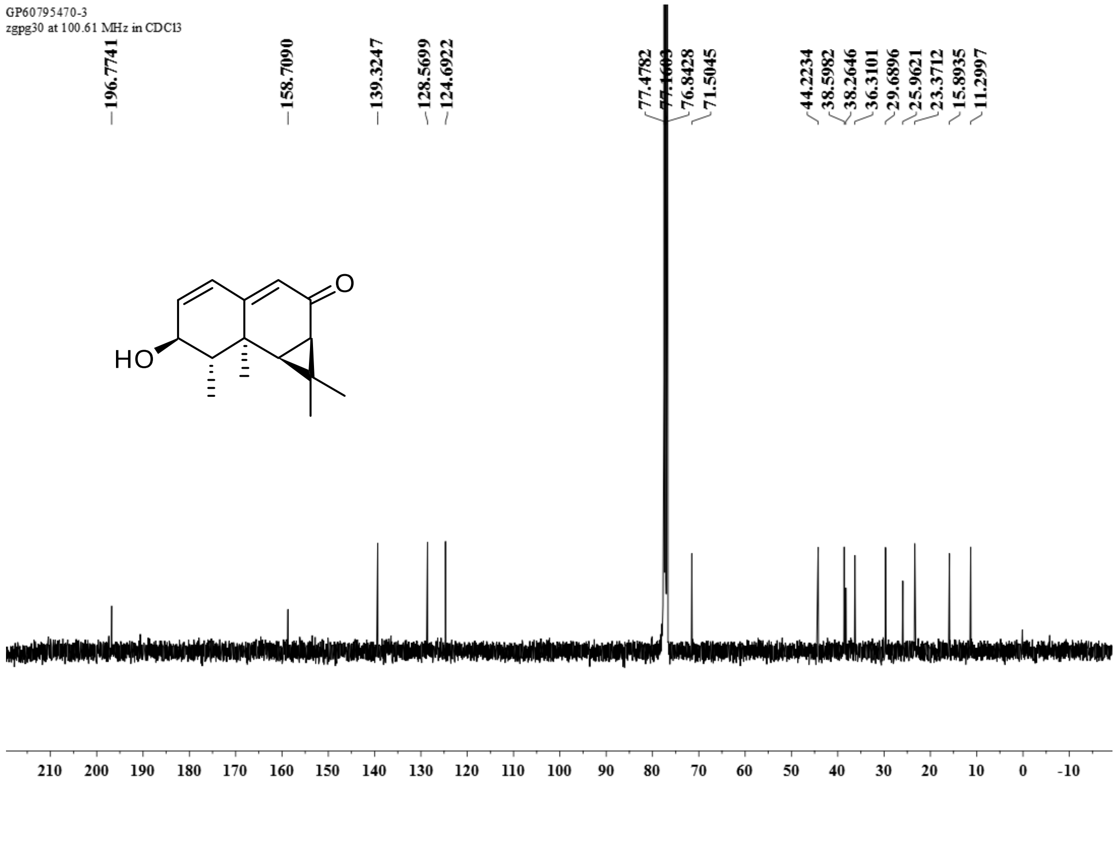

Figure S15. HSQC spectrum of 3-hydroxylkanshone H (**6**)

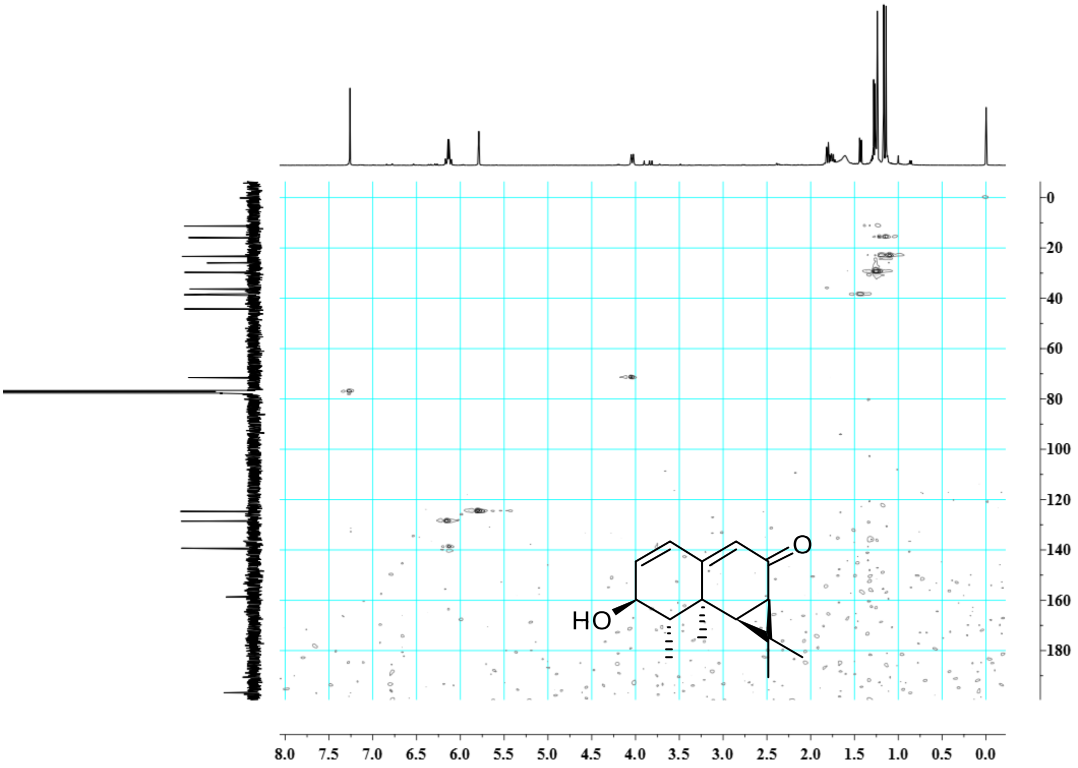

Figure S16. HMBC spectrum of 3-hydroxylkanshone H (**6**)

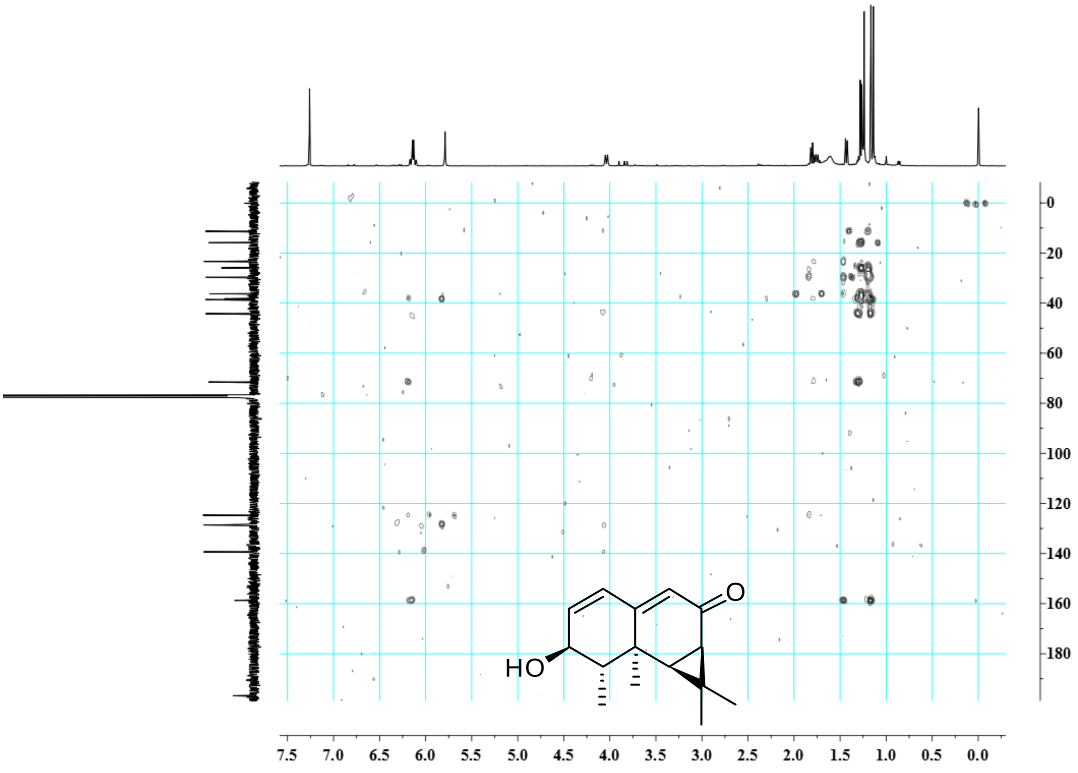

Figure S17. NOESY spectrum of 3-hydroxylkanshone H (**6**)

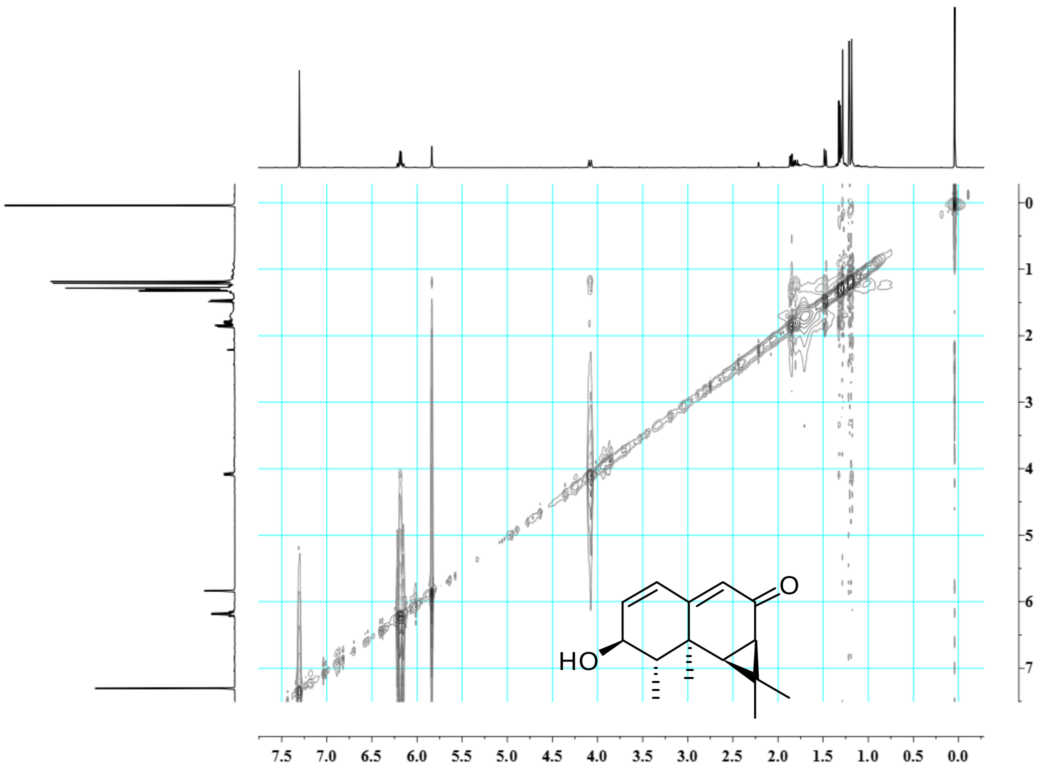

Figure S18. (+)-HRESIMS of 3-oxokanshone H (**7**)

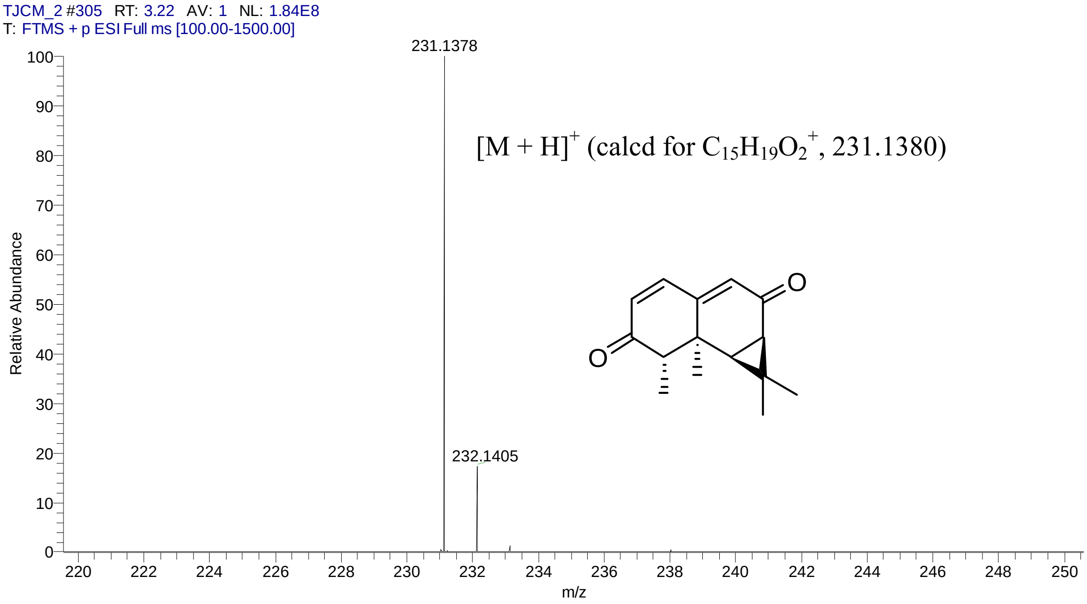

Figure S19. ^{1}H NMR (400.13 MHz, $CDCl_3$) spectrum of 3-oxokanshone H (**7**)

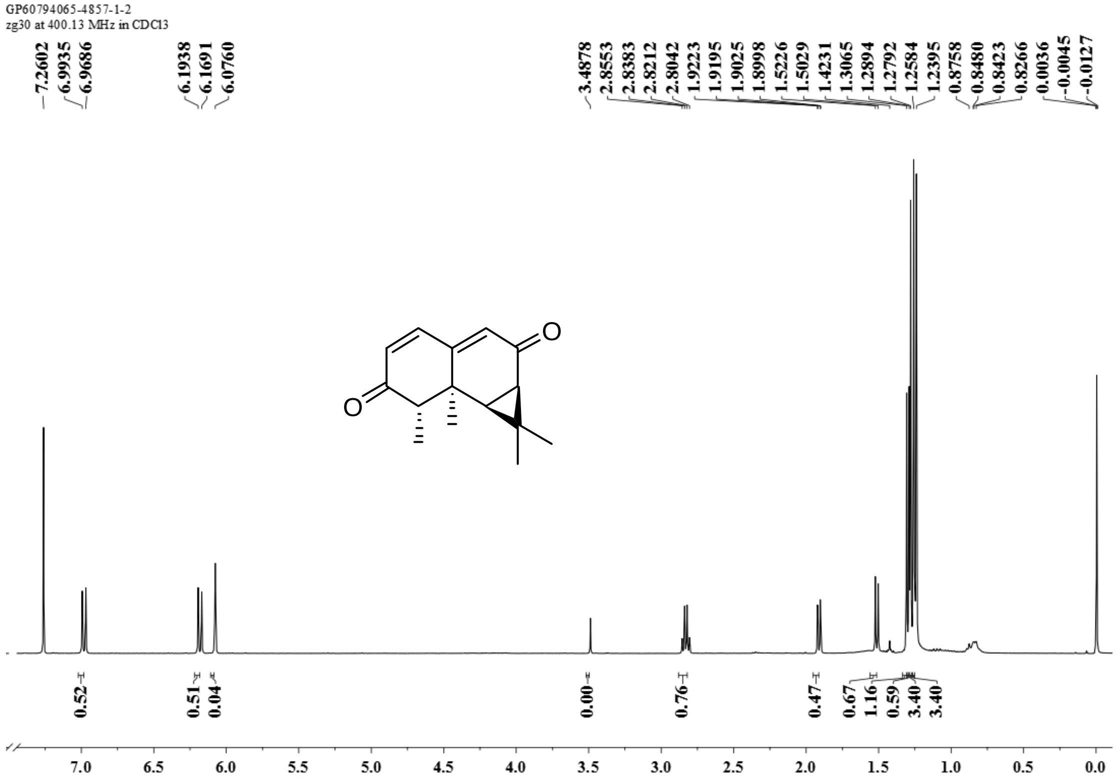

Figure S20. ^{13}C NMR (100.61 MHz, $CDCl_3$) spectrum of 3-oxokanshone H (**7**)

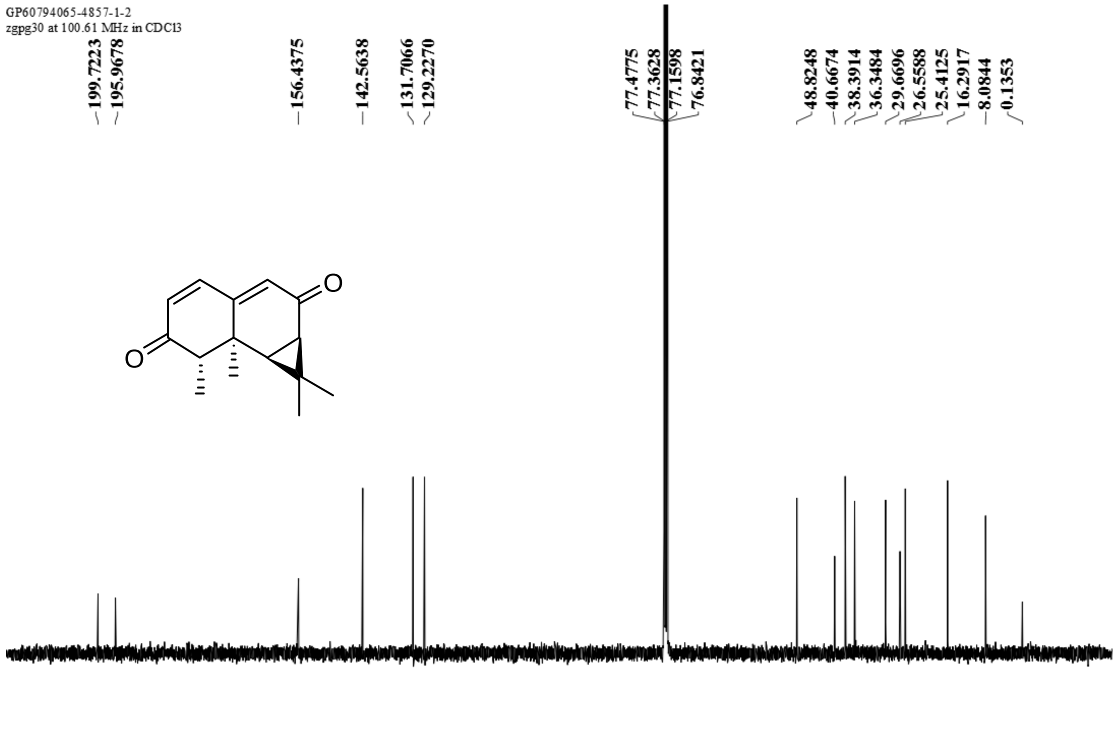

Figure S21. HSQC spectrum of 3-oxokanshone H (**7**)

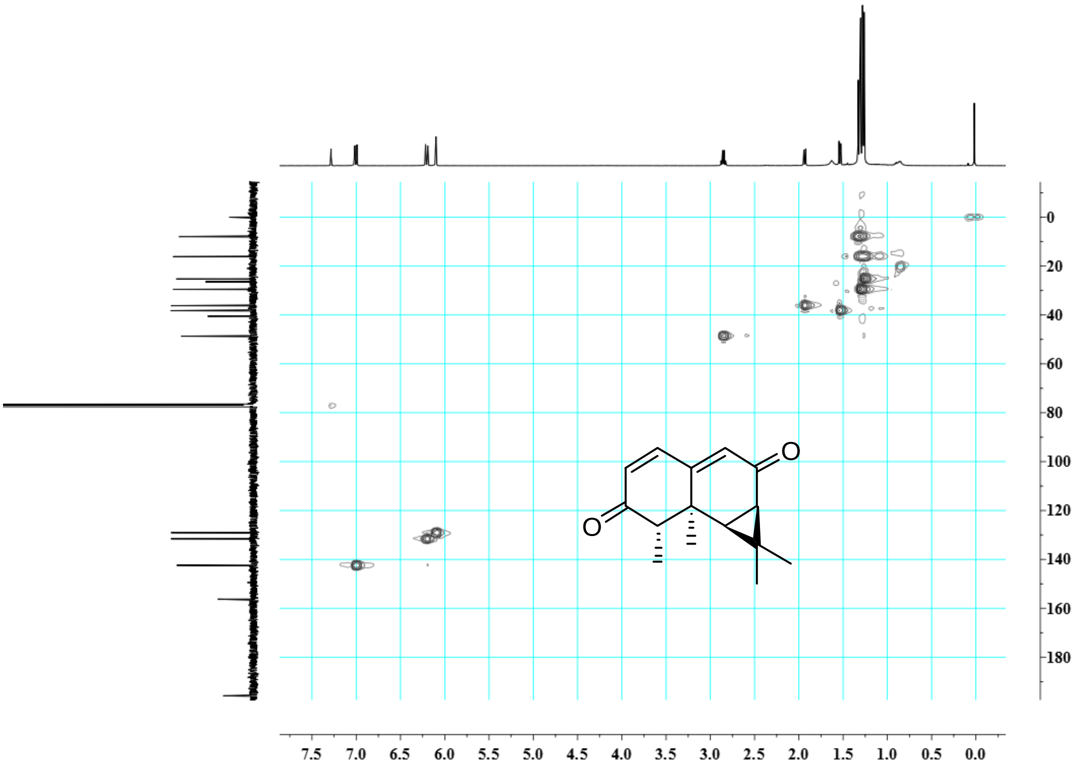

Figure S22. HMBC spectrum of 3-oxokanshone H (**7**)

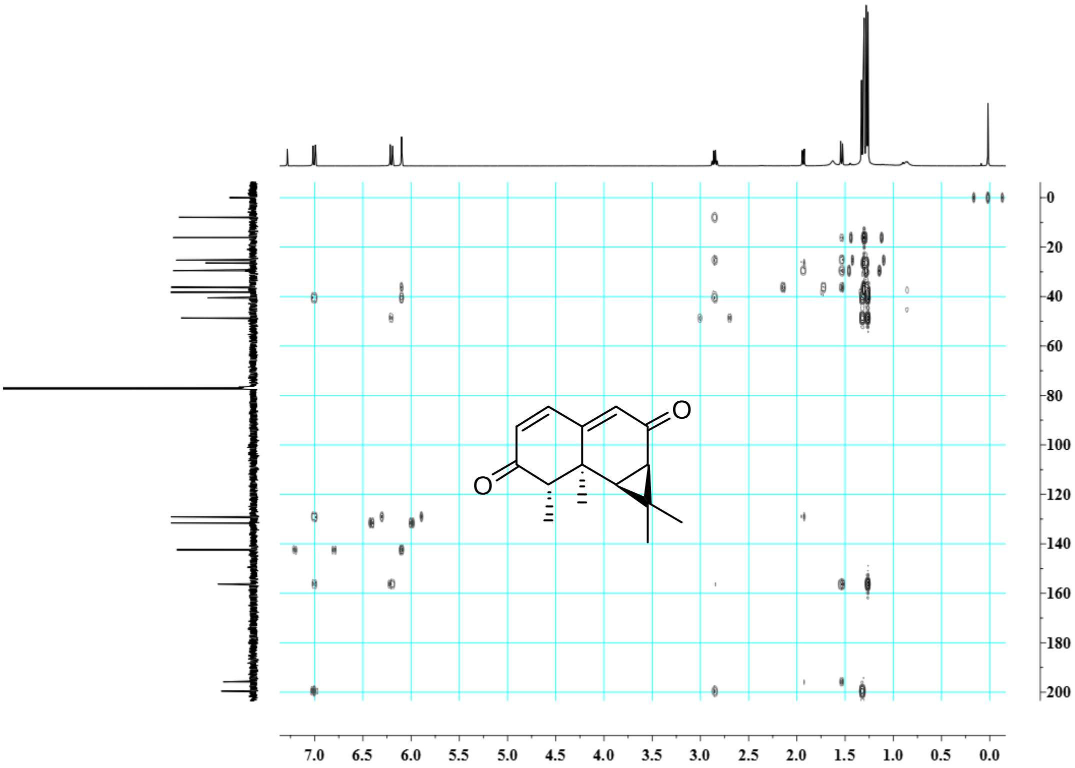

Figure S23. NOESY spectrum of 3-oxokanshone H (**7**)

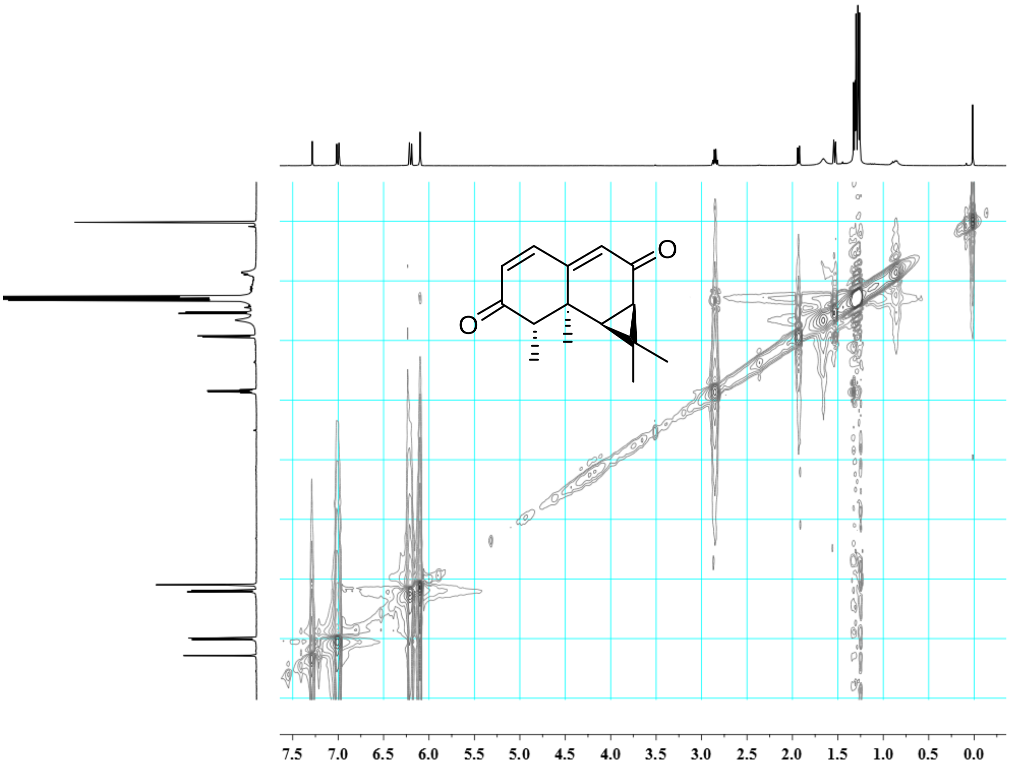

Figure S24. HRESIMS of 9*β*-debilon (**14**)

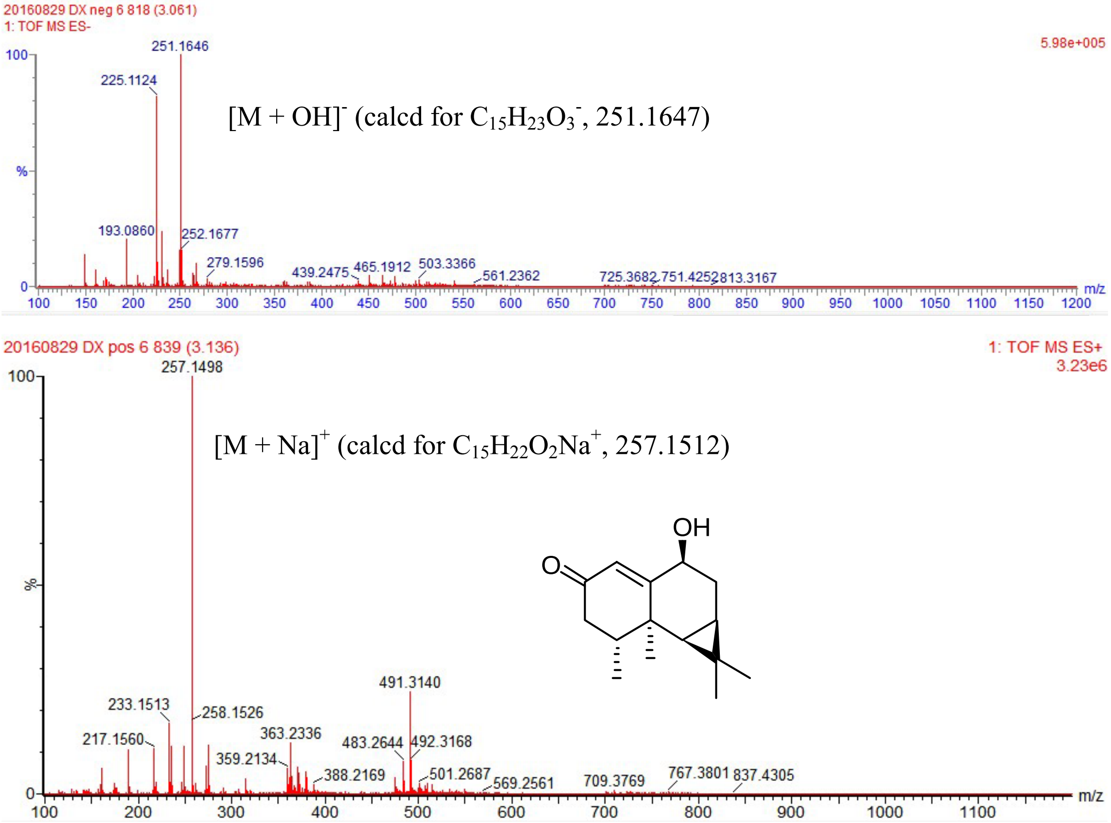

Figure S25. ^{1}H NMR (400.13 MHz, $CDCl_3$) spectrum of 9*β*-debilon (**14**)

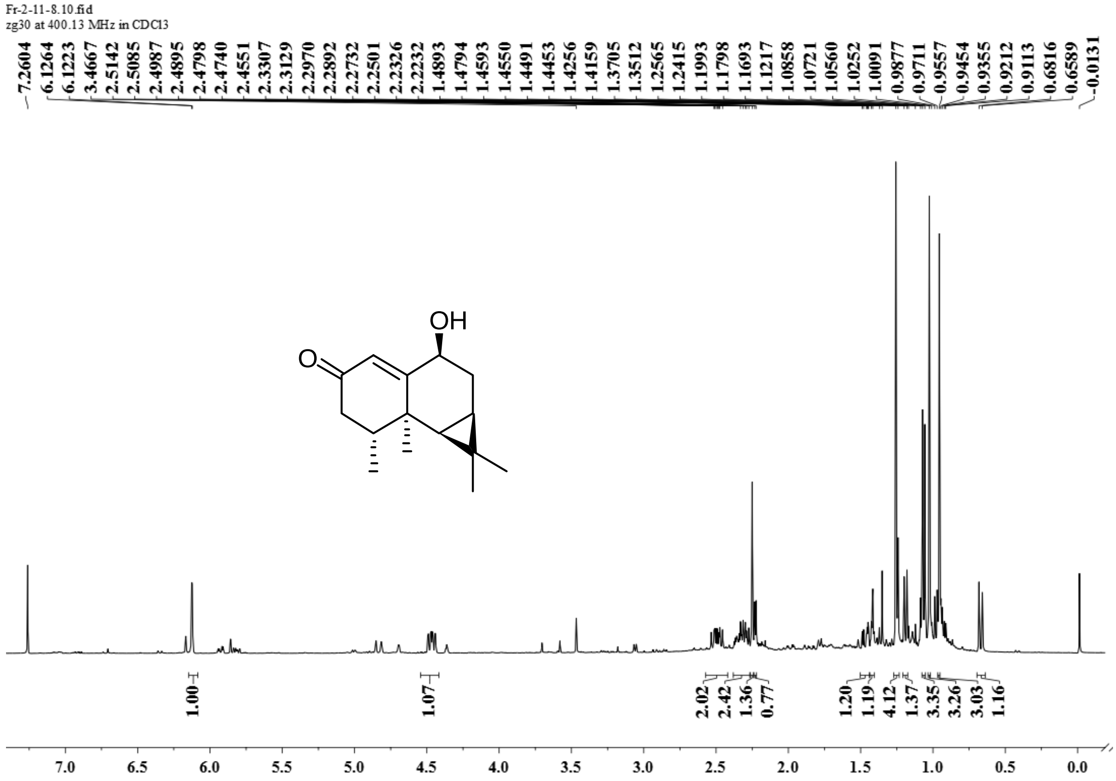

Figure S26. ^{13}C NMR (100.62 MHz, $CDCl_3$) spectrum of 9*β*-debilon (**14**)

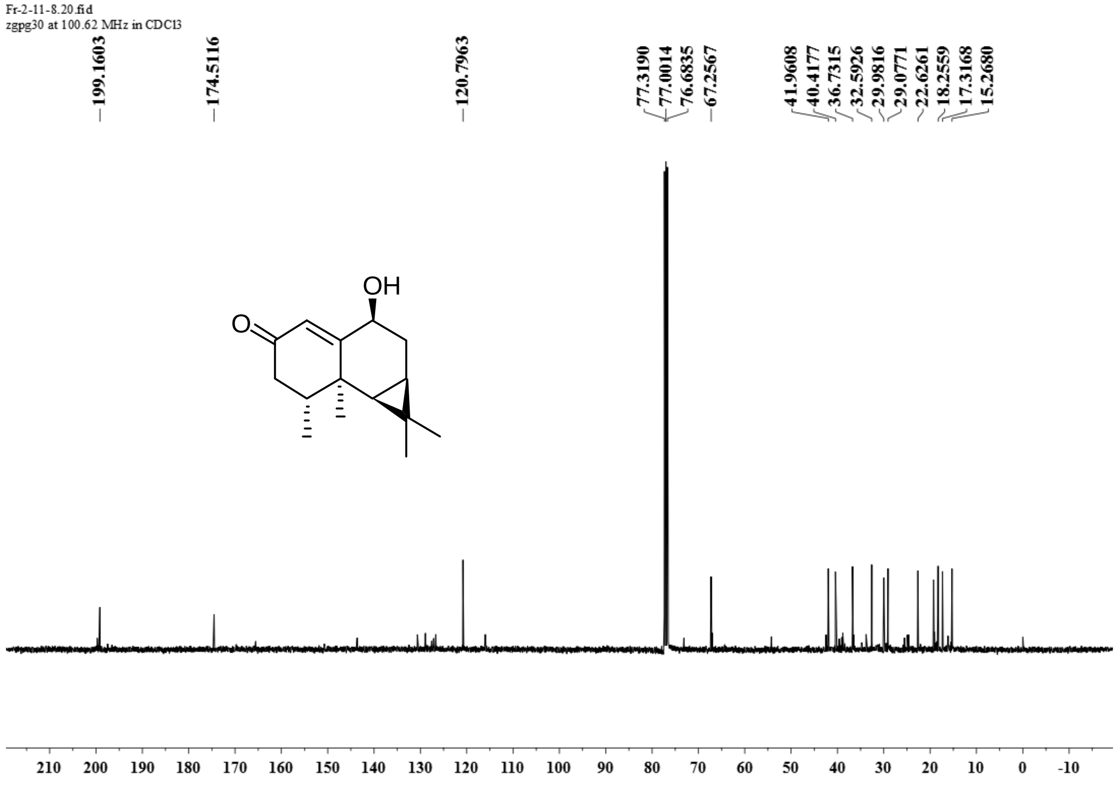

Figure S27. HSQC spectrum of 9*β*-debilon (**14**)

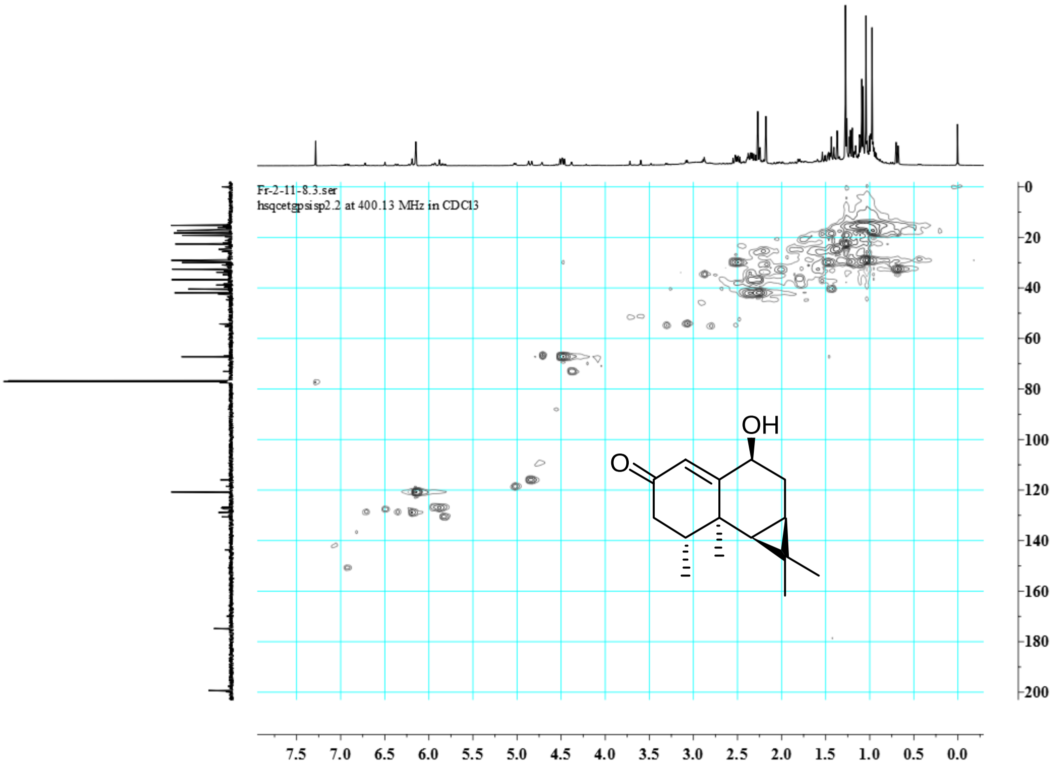

Figure S28. HMBC spectrum of 9*β*-debilon (**14**)

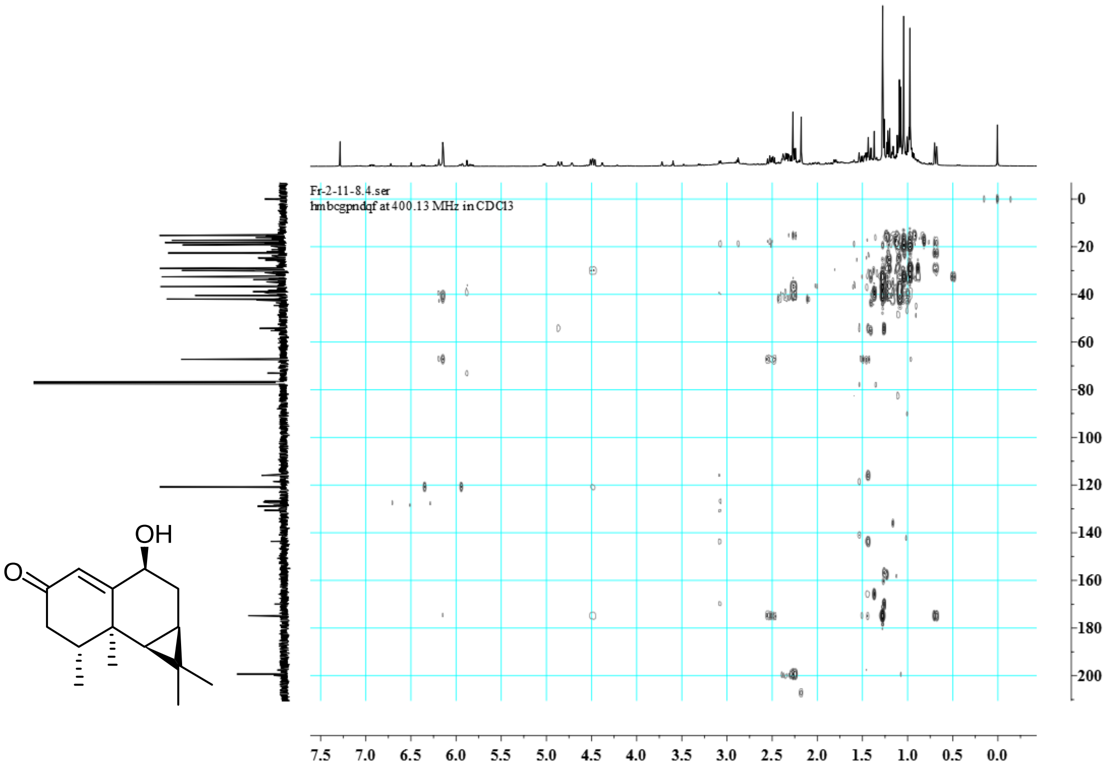

Figure S29. HRESIMS of aristolanhydride (**18**)

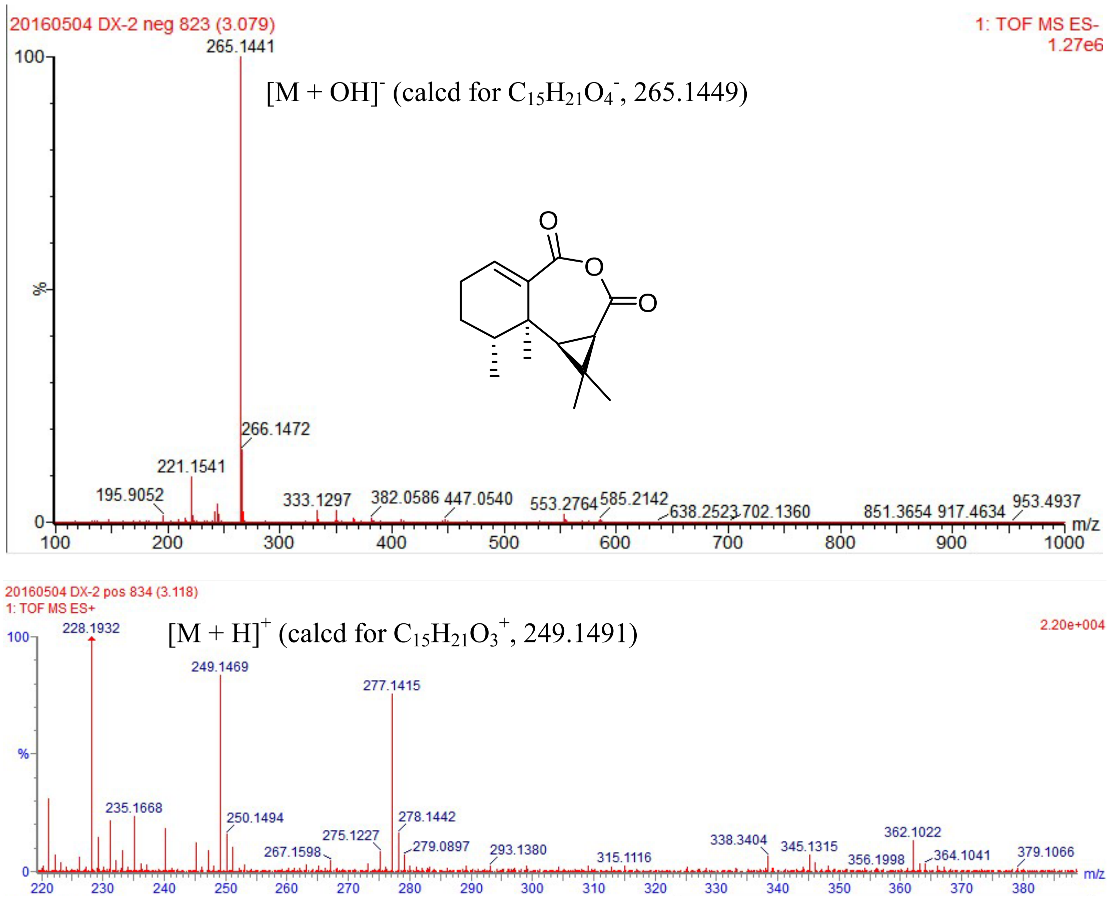

Figure S30. ^{1}H NMR (400.13 MHz, CD_3OD) spectrum of aristolanhydride (**18**)

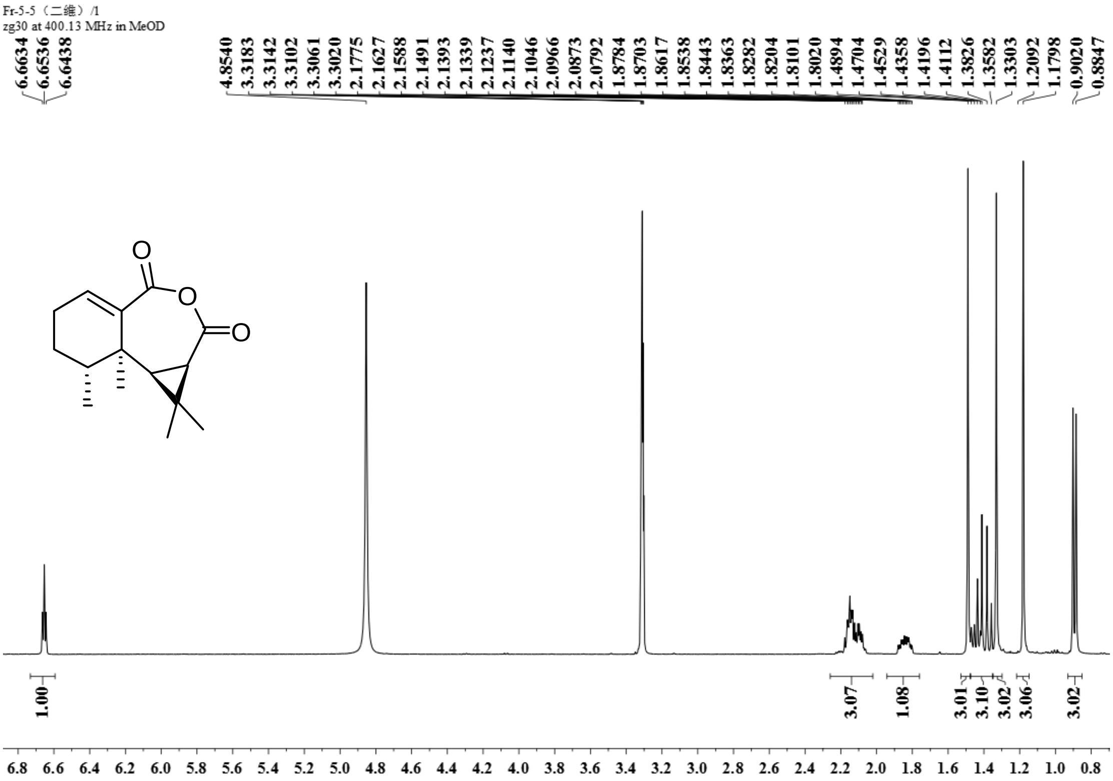

Figure S31. ^{13}C NMR (100.62 MHz, CD_3OD) spectrum of aristolanhydride (**18**)

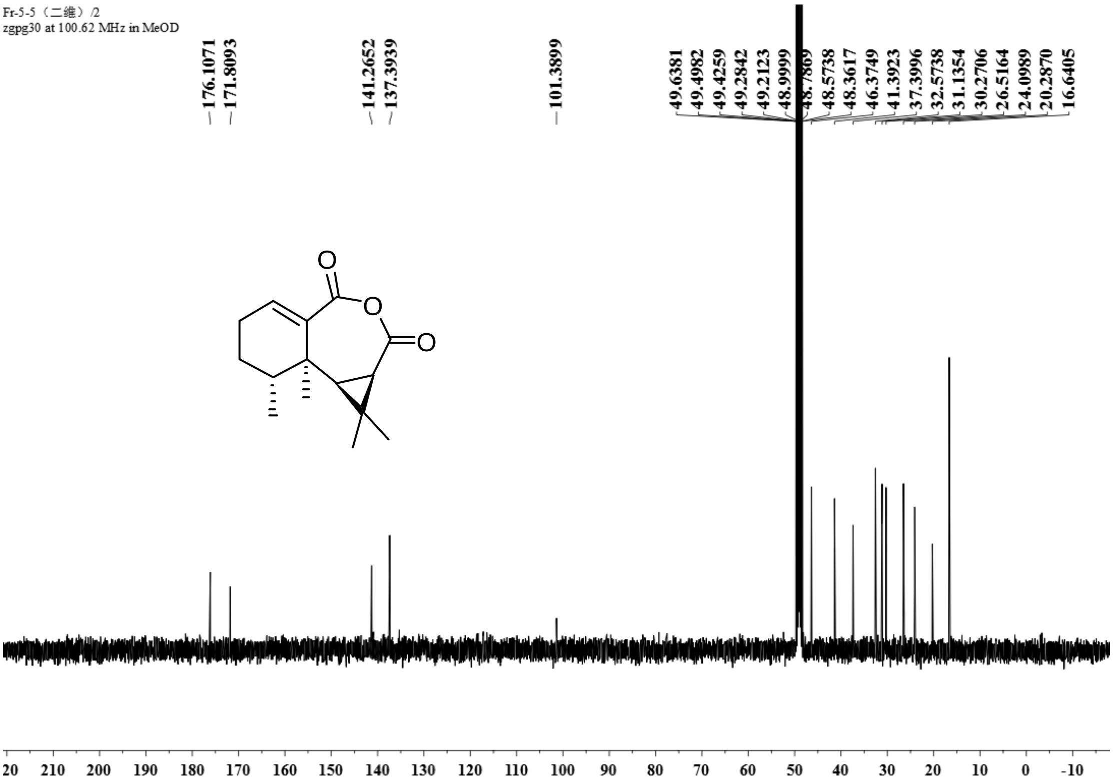

Figure S32. HSQC spectrum of aristolanhydride (**18**)

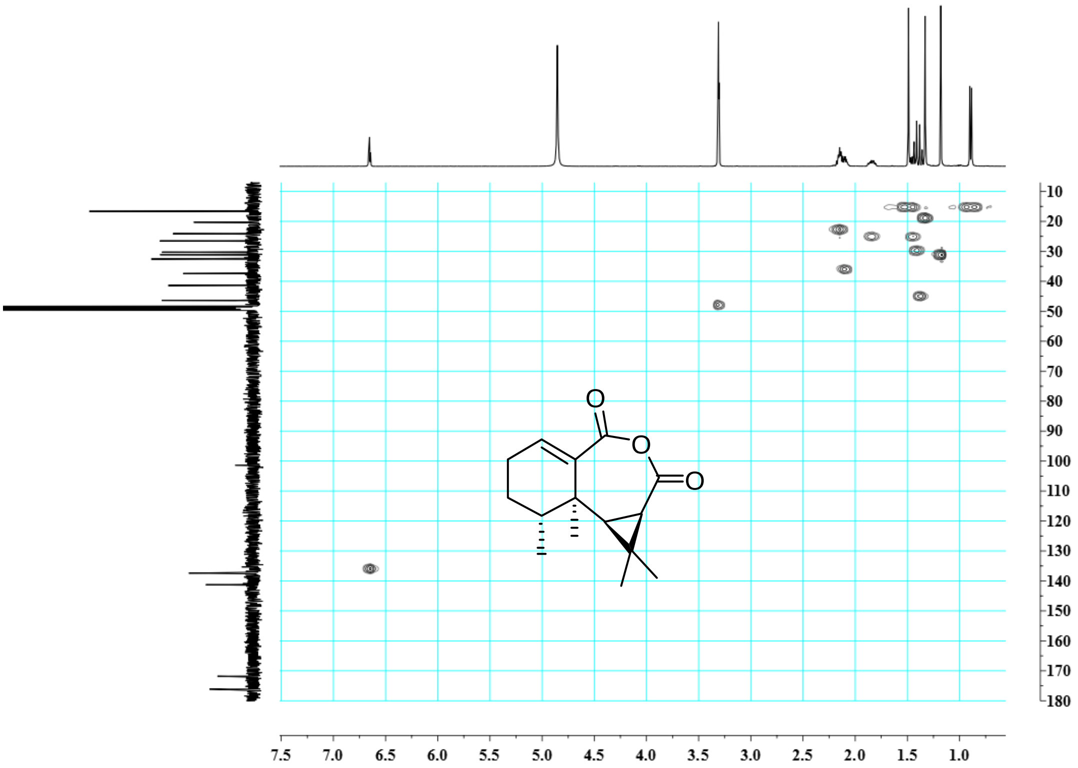

Figure S33. HMBC spectrum of aristolanhydride (**18**)

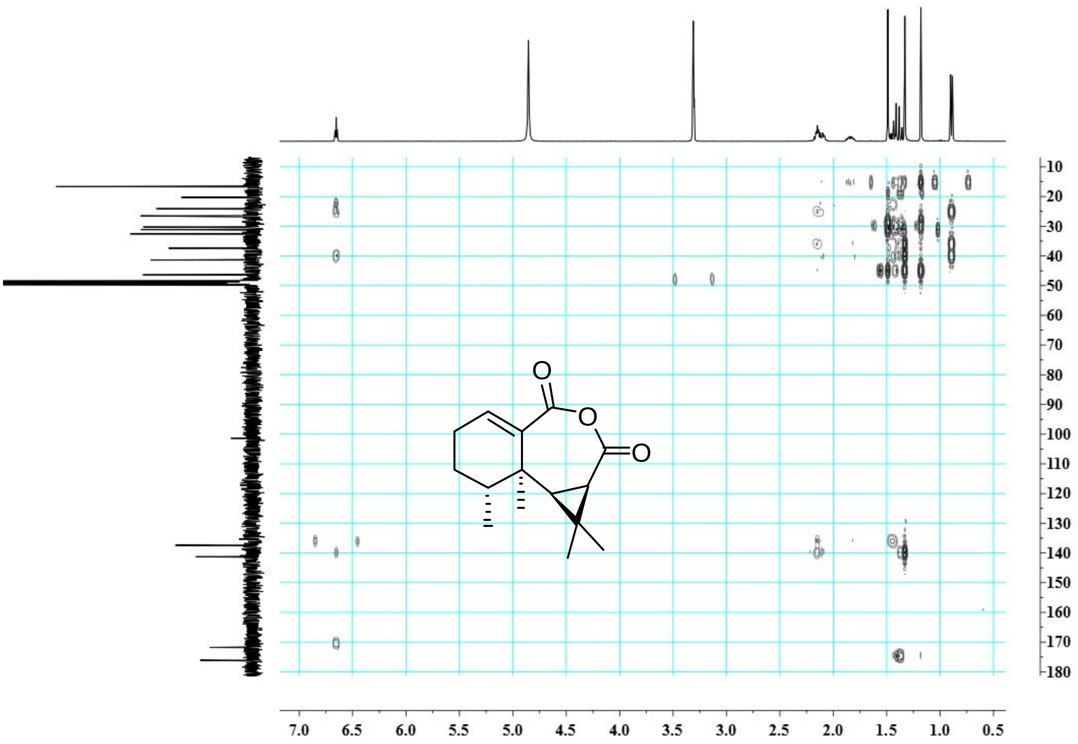

Figure S34. NOESY spectrum of aristolanhydride (**18**)

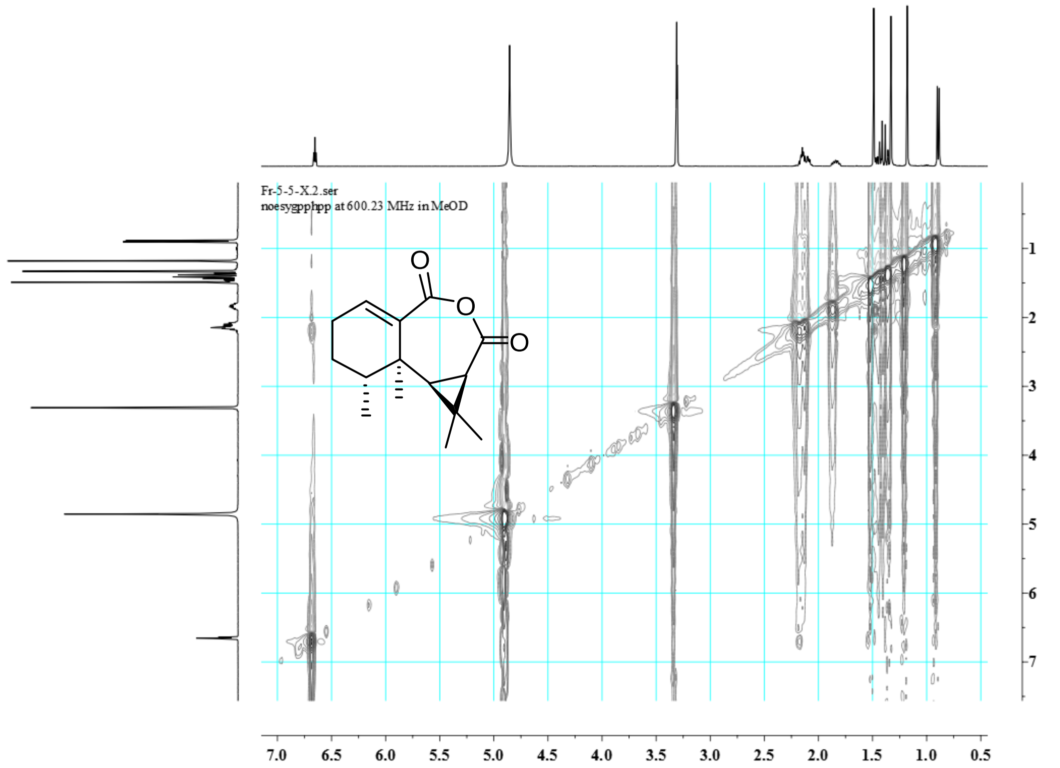

Figure S35. (-)-HRESIMS of epoxynardosinone (**19**)

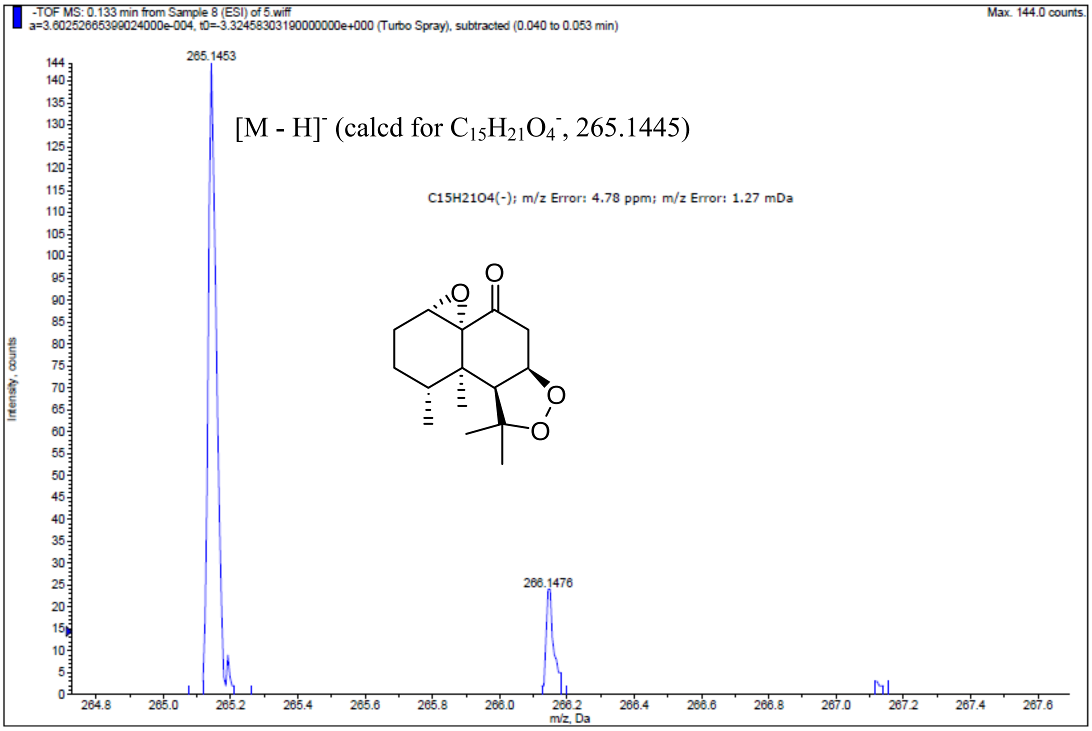

Figure S36. ^{1}H NMR (400.13 MHz, $CDCl_3$) spectrum of epoxynardosinone (**19**)

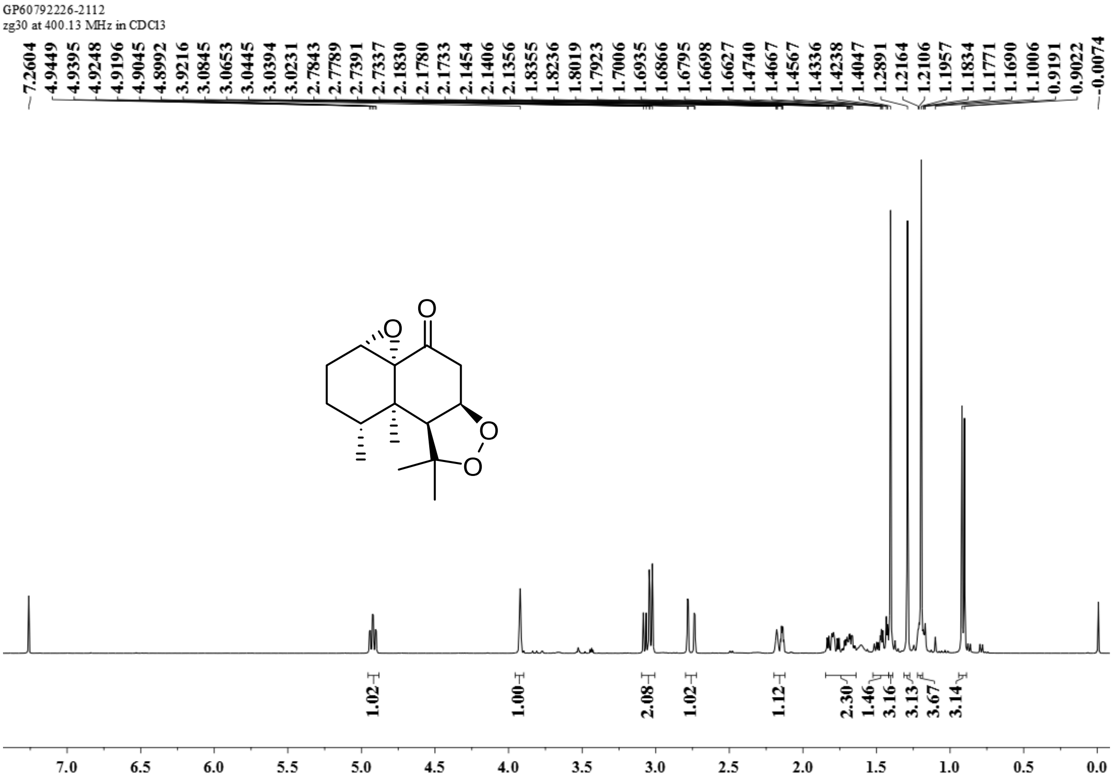

Figure S37. ^{13}C NMR (100.61 MHz, CDCl$_3$) spectrum of epoxynardosinone (**19**)

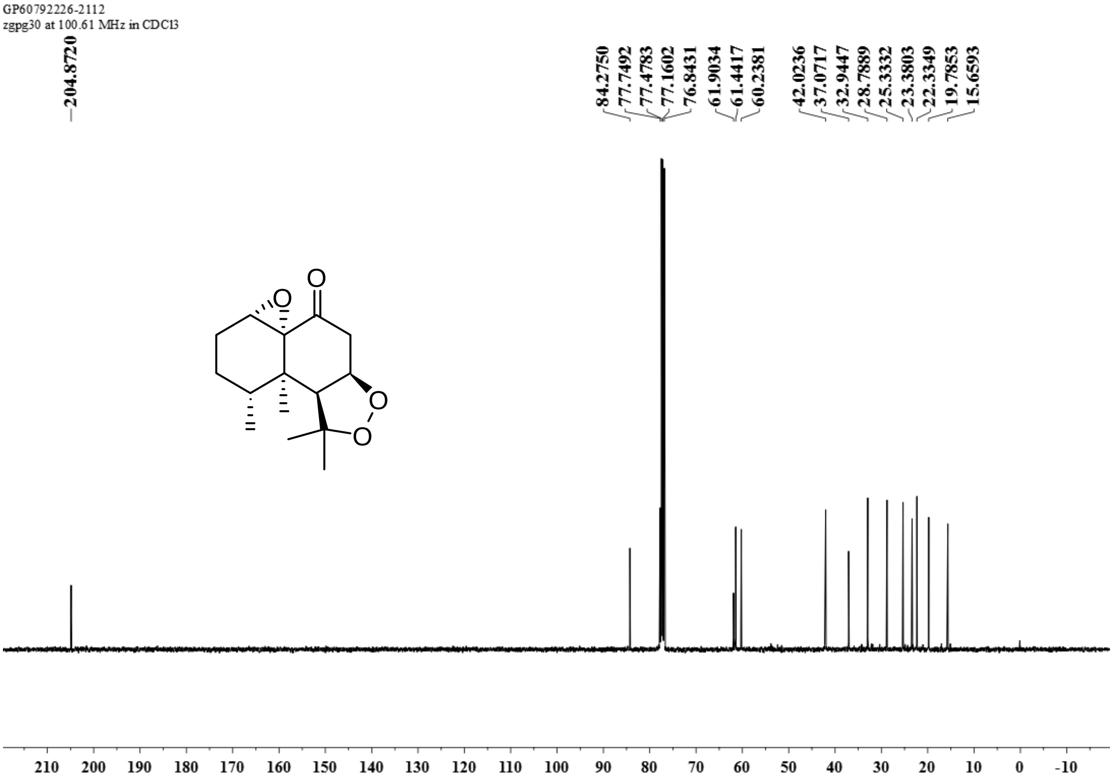

Figure S38. HSQC spectrum of epoxynardosinone (**19**)

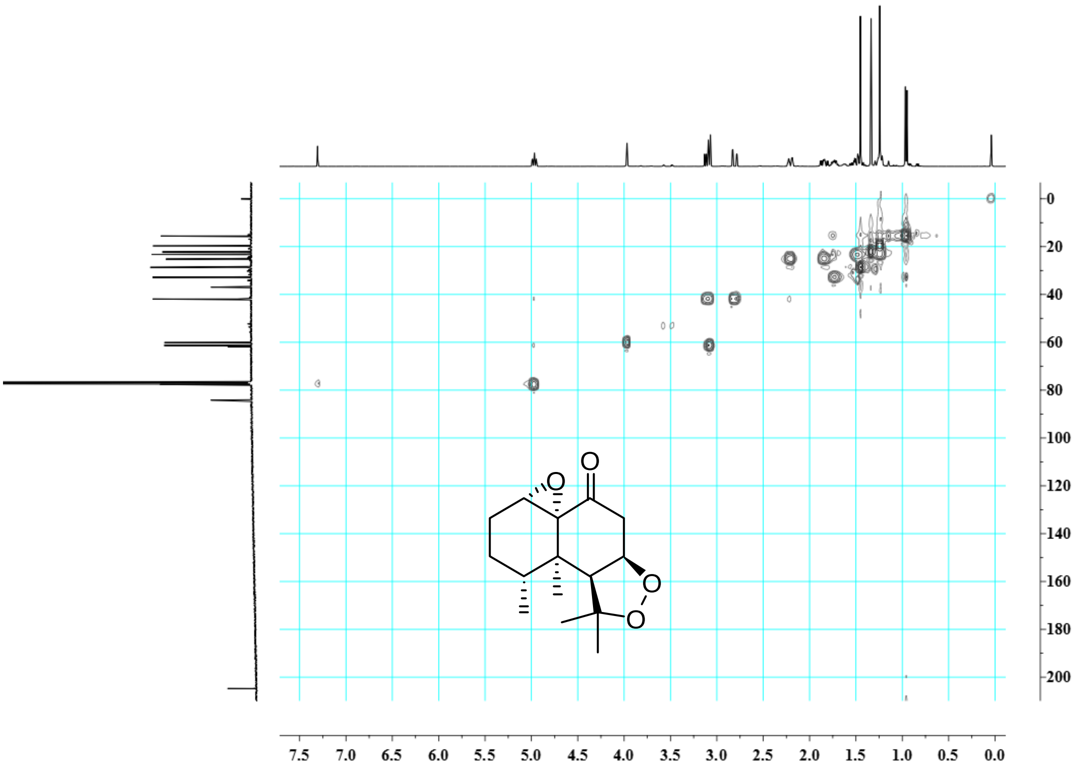

Figure S39. HMBC spectrum of epoxynardosinone (**19**)

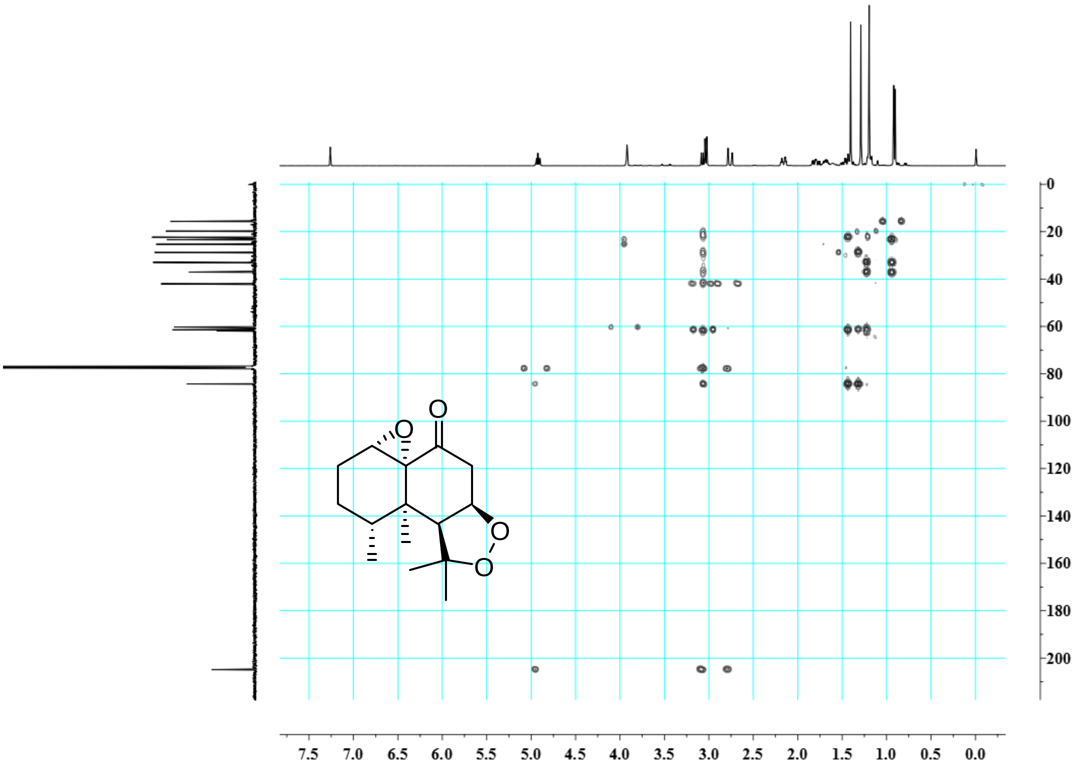

Figure S40. NOESY spectrum of epoxynardosinone (**19**)

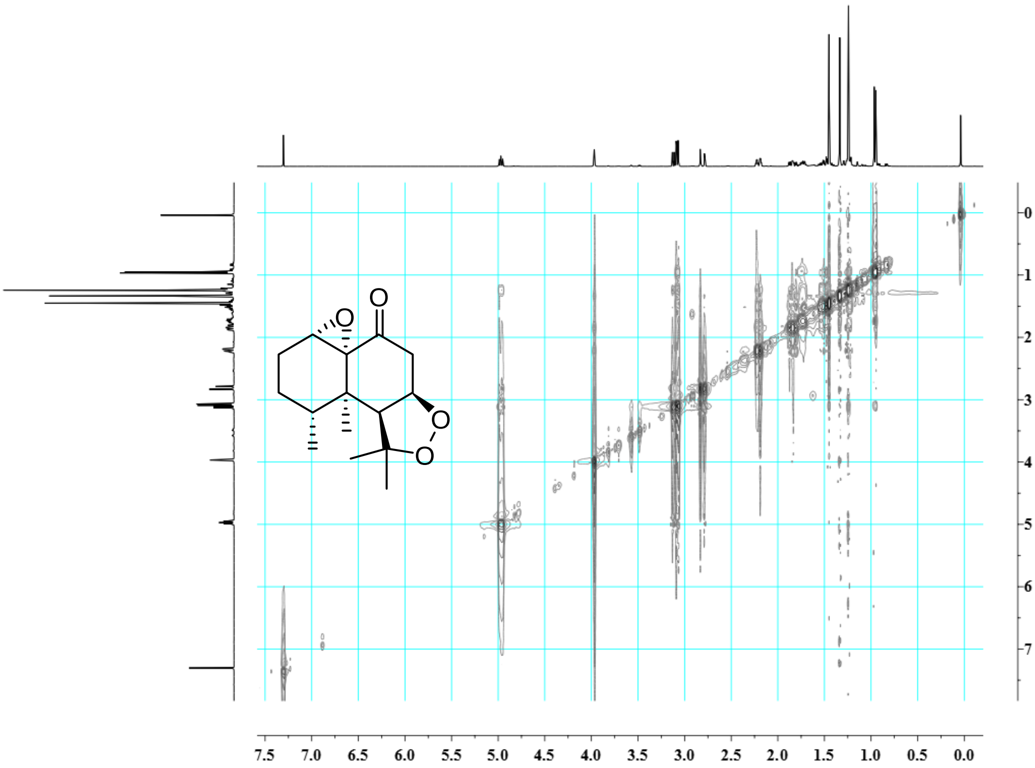

Figure S41. HRESIMS of nardosinonetriol (**22**)

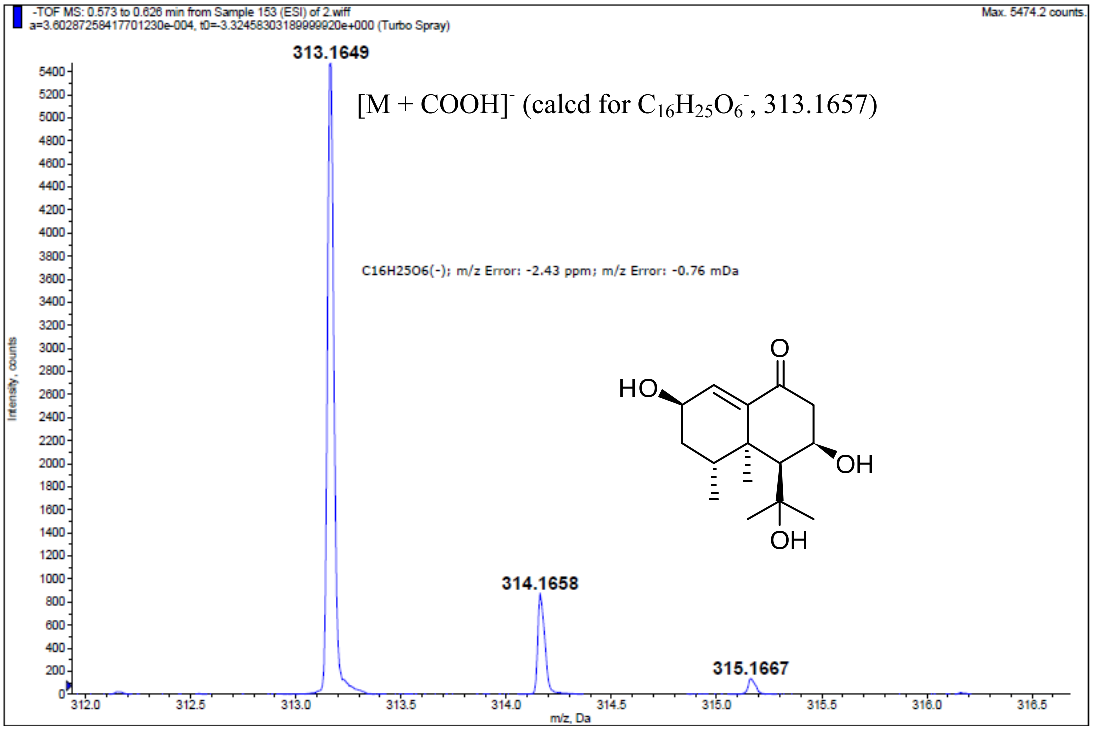

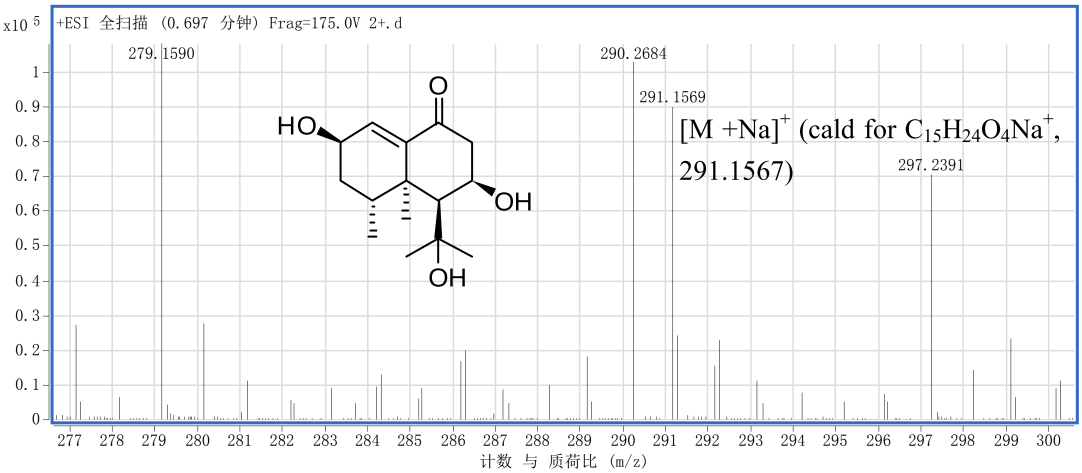

Figure S42. ^{1}H NMR (600.23 MHz, DMSO-d_6) spectrum of nardosinonetriol (**22**)

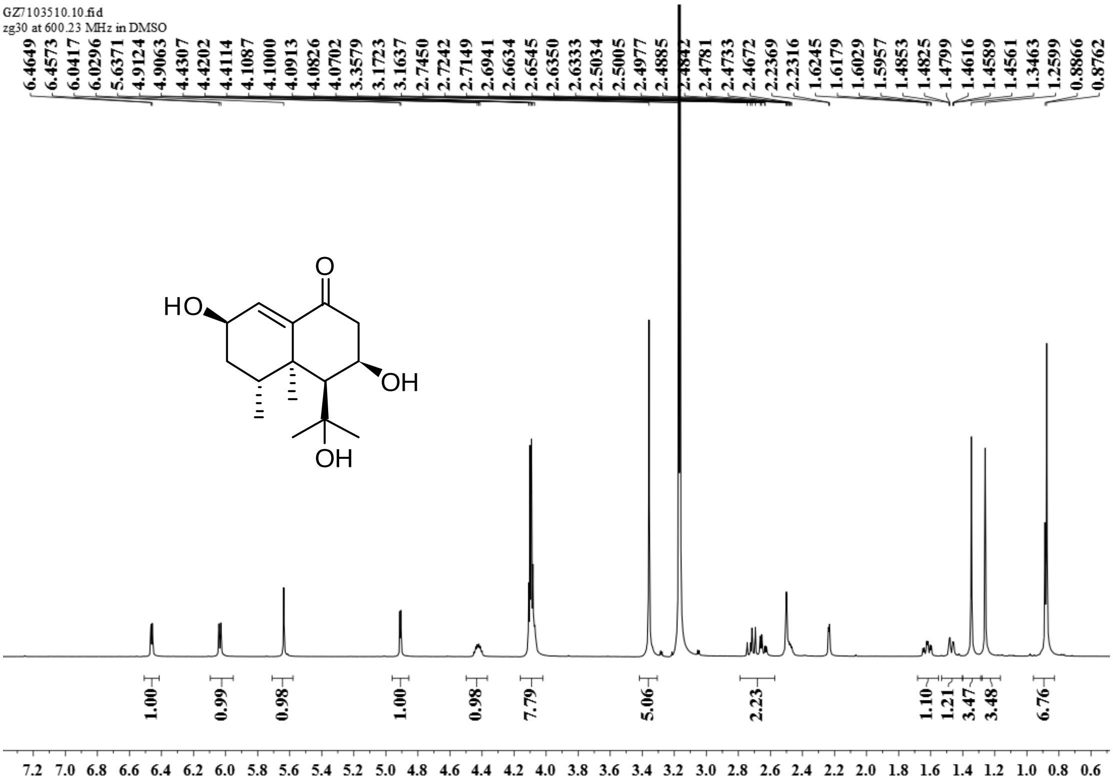

Figure S43. ^{13}C NMR (150.94 MHz, DMSO-d_6) spectrum of nardosinonetriol (**22**)

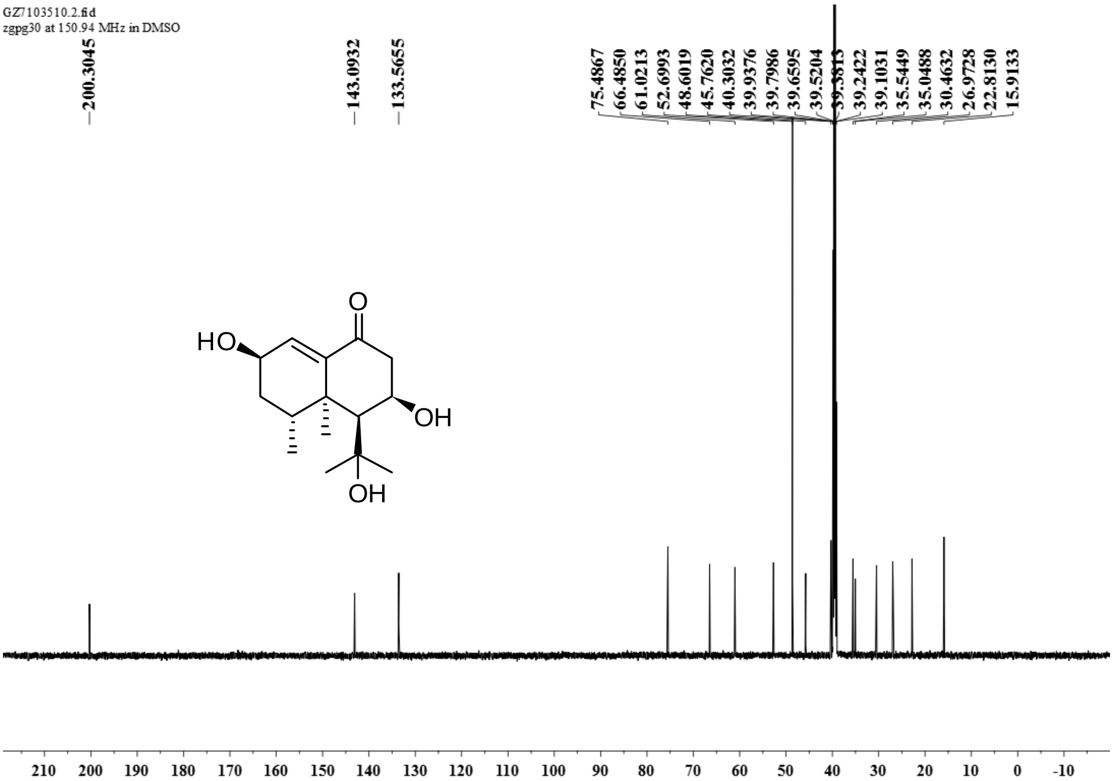

Figure S44. HSQC spectrum of nardosinonetriol (**22**)

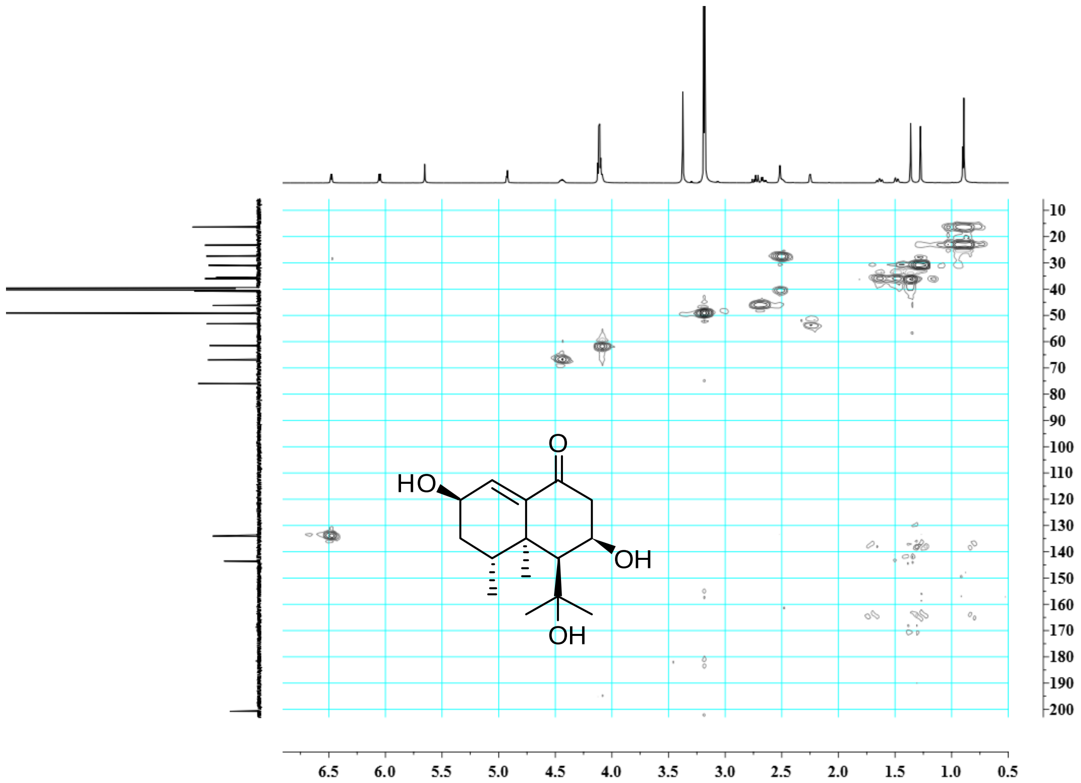

Figure S45. HMBC spectrum of nardosinonetriol (**22**)

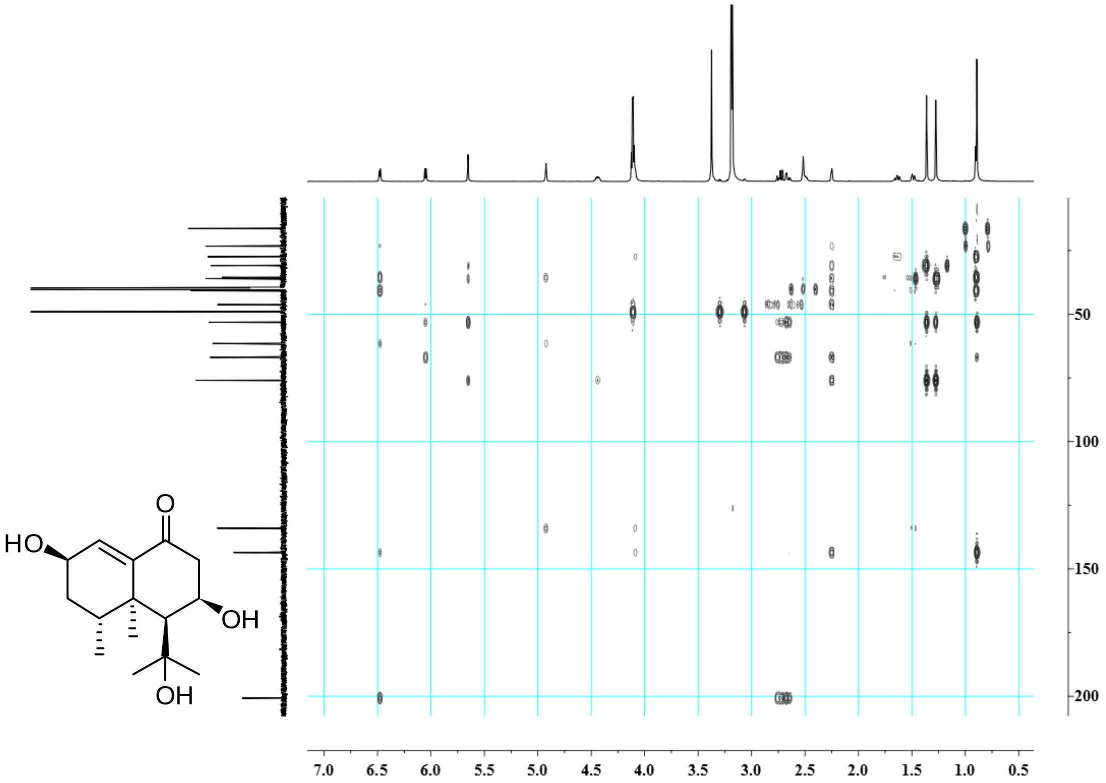

Figure S46. NOESY spectrum of nardosinonetriol (**22**)

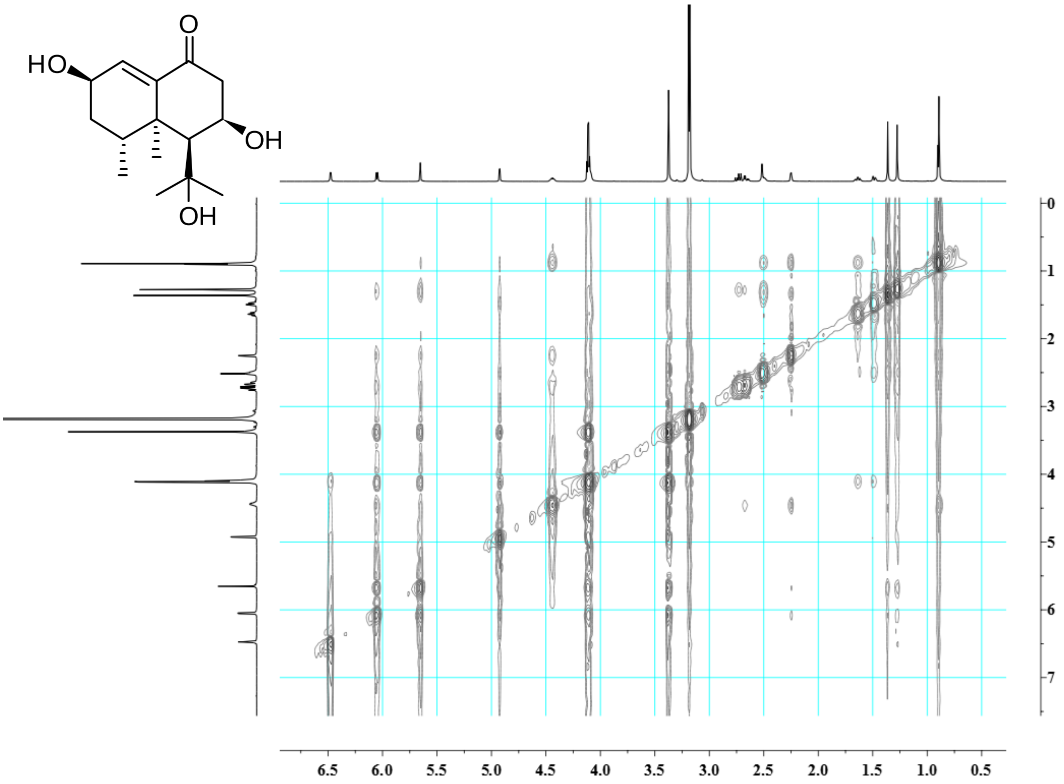

Figure S47. (+)-HRESIMS of 7-oxonardosinone (**23**)

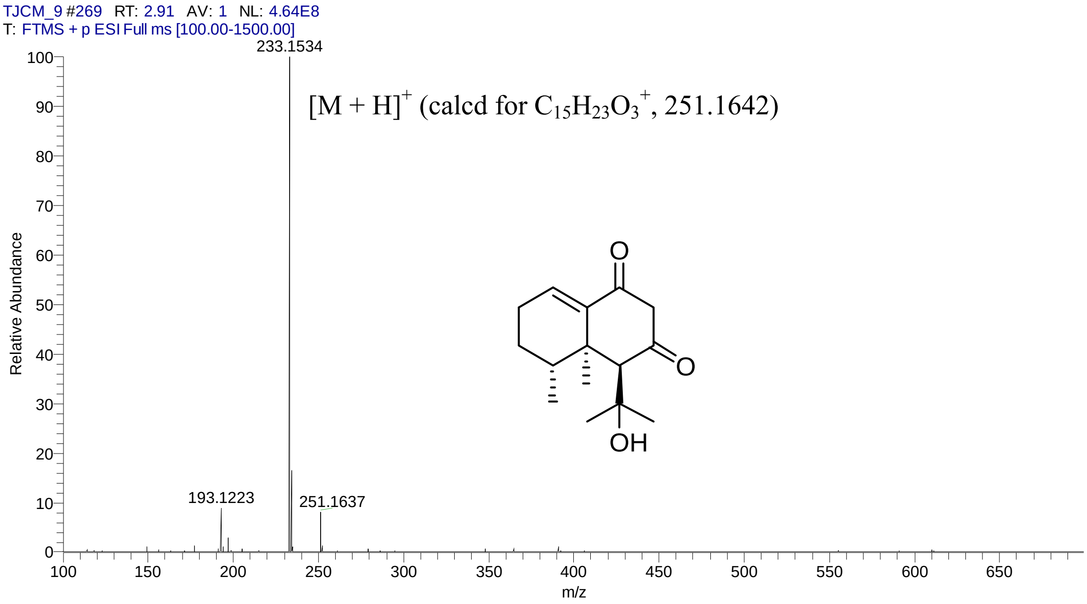

Figure S48. ^{1}H NMR (600.25 MHz, $CDCl_3$) spectrum of 7-oxonardosinone (**23**)

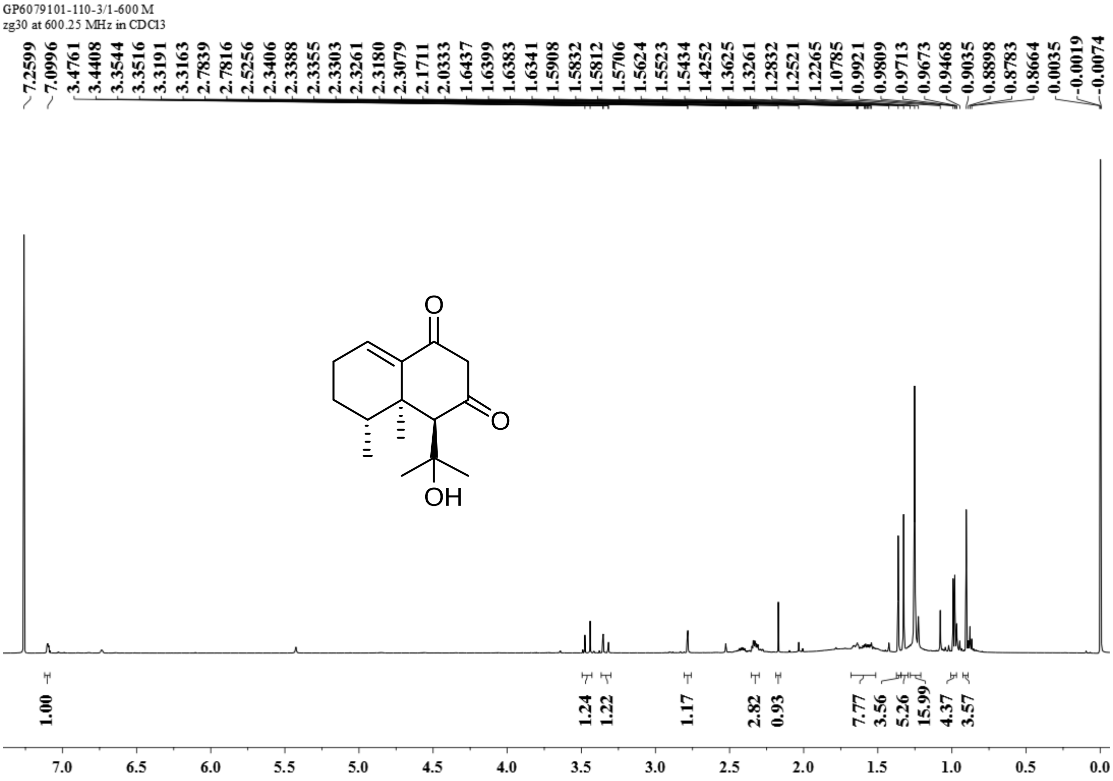

Figure S49. ^{13}C NMR (150.95 MHz, $CDCl_3$) spectrum of 7-oxonardosinone (**23**)

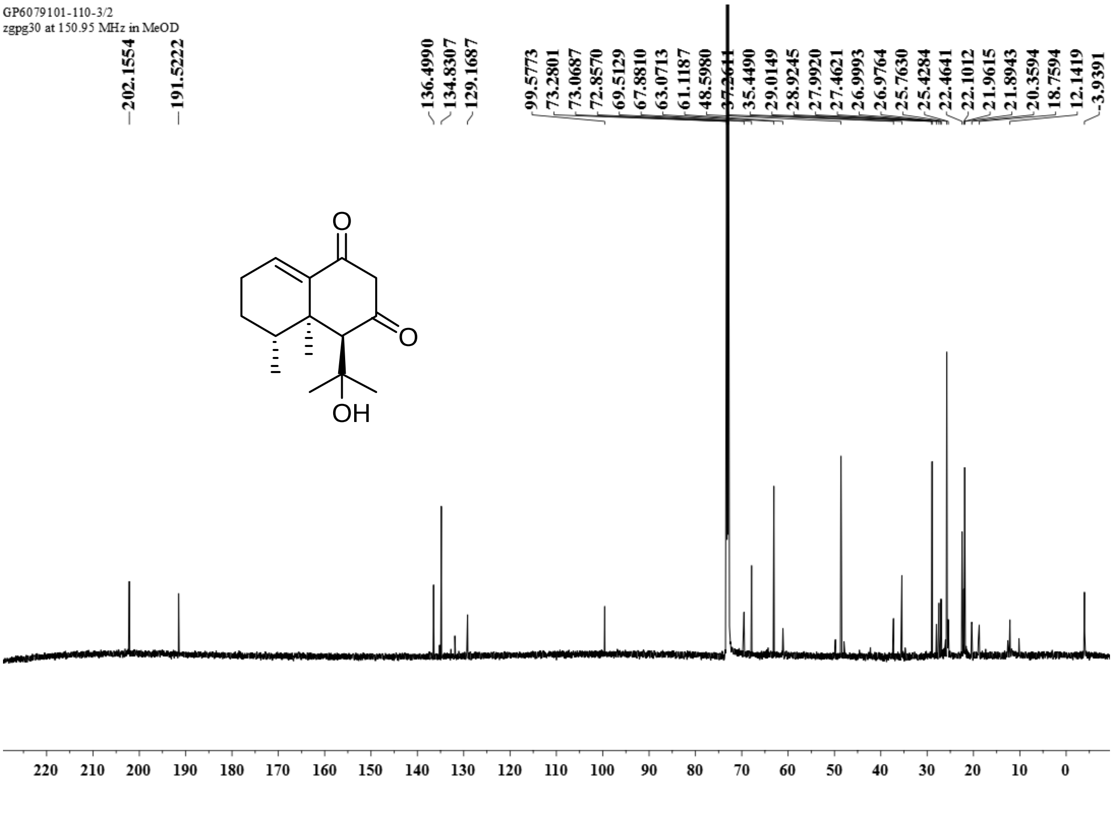

Figure S50. HSQC spectrum of 7-oxonardosinone (**23**)

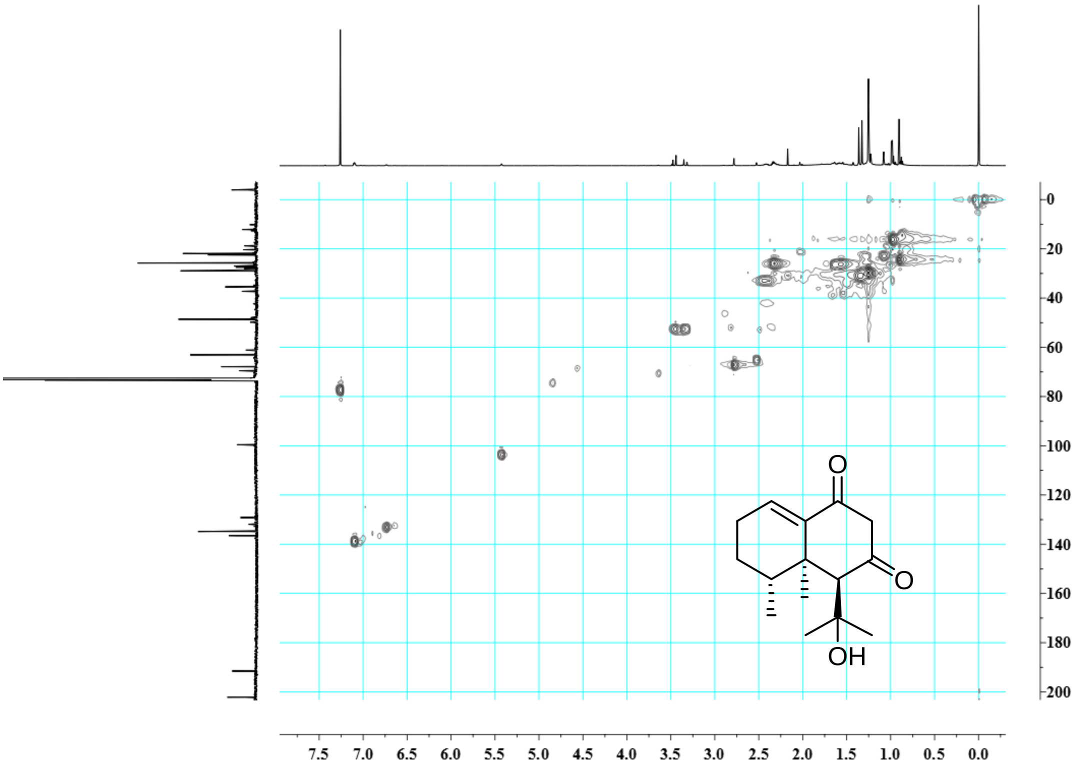

Figure S51. HMBC spectrum of 7-oxonardosinone (**23**)

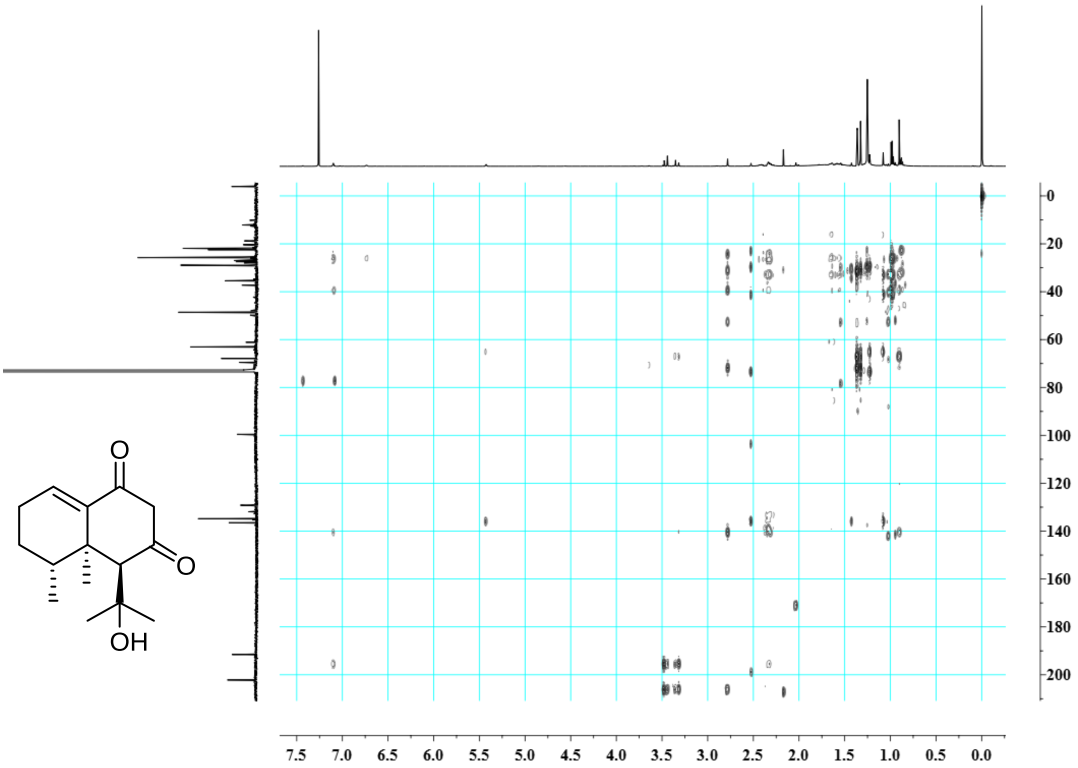

Figure S52. NOESY spectrum of 7-oxonardosinone (**23**)

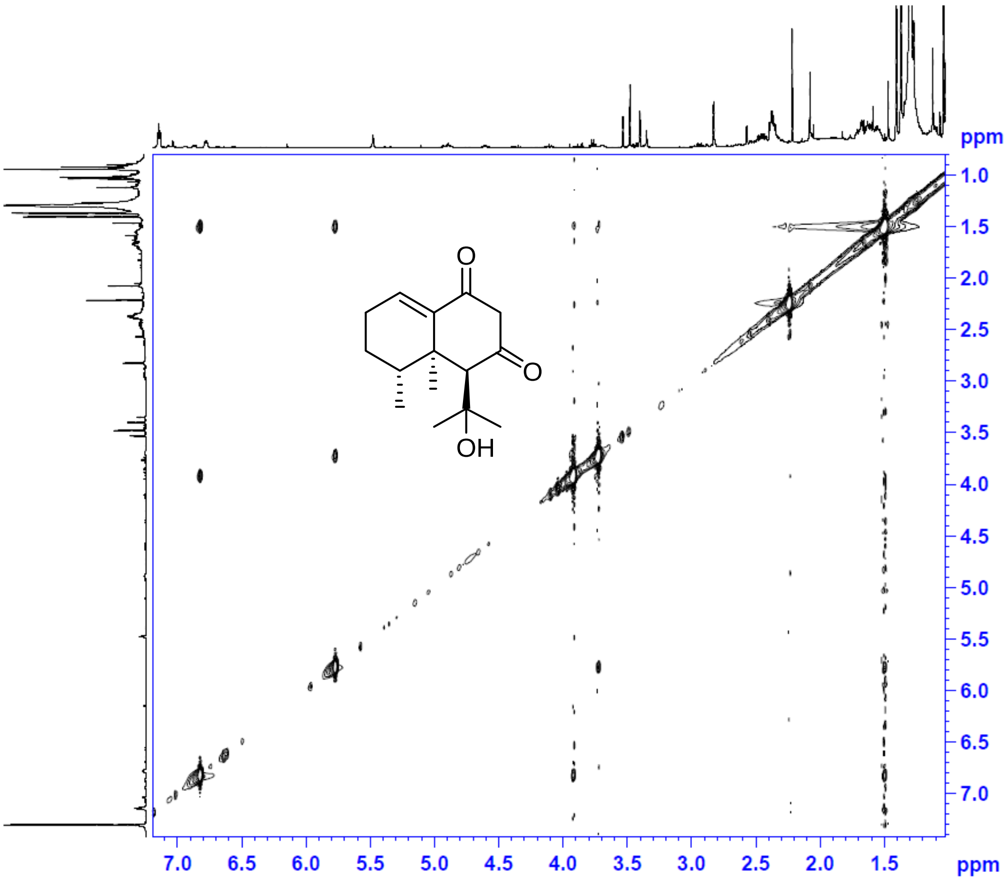

Figure S53. (+)-HRESIMS of 7-oxonardosinoperoxide (**24**)

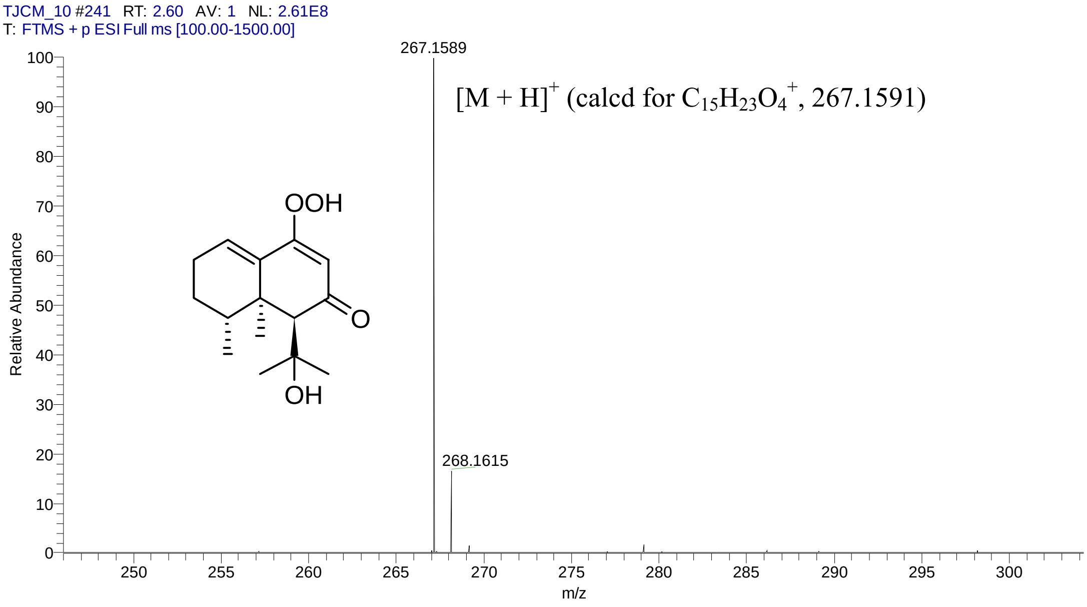

Figure S54. ^{1}H NMR (400.13 MHz, DMSO-d_6) spectrum of 7-oxonardosinoperoxide (**24**)

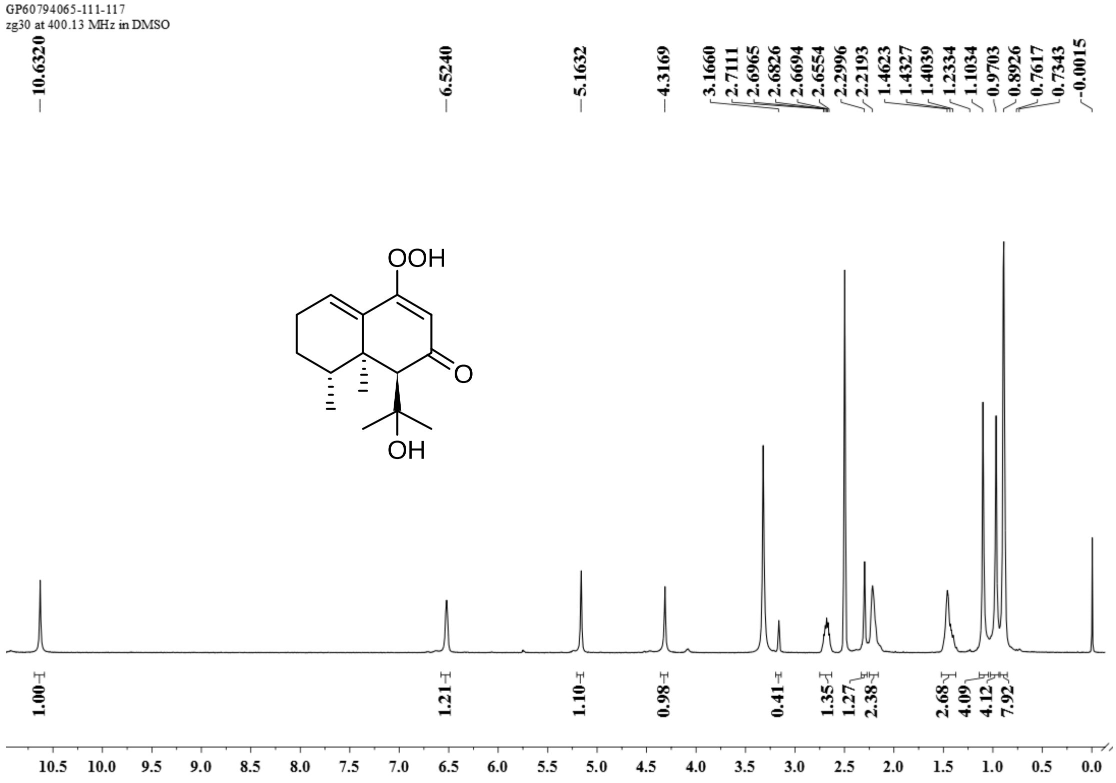

Figure S55. ^{13}C NMR (100.61 MHz, DMSO-d_6) spectrum of 7-oxonardosinoperoxide (**24**)

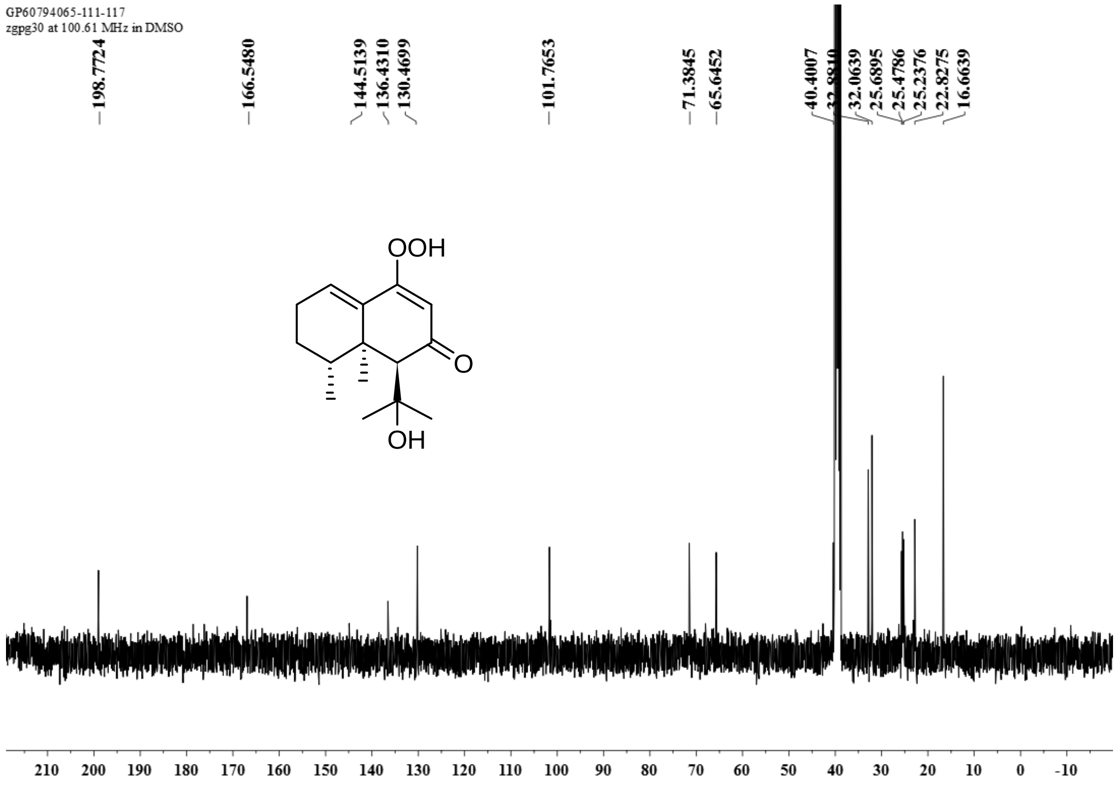

Figure S56. HSQC spectrum of 7-oxonardosinoperoxide (**24**)

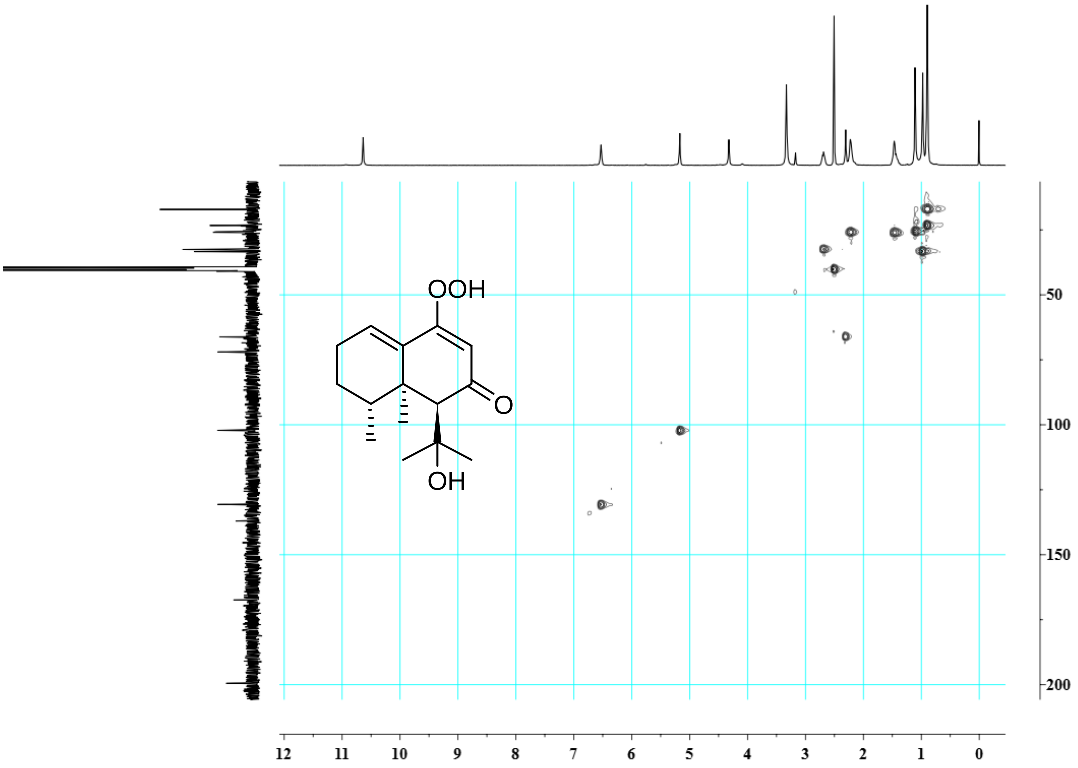

Figure S57. HMBC spectrum of 7-oxonardosinoperoxide (**24**)

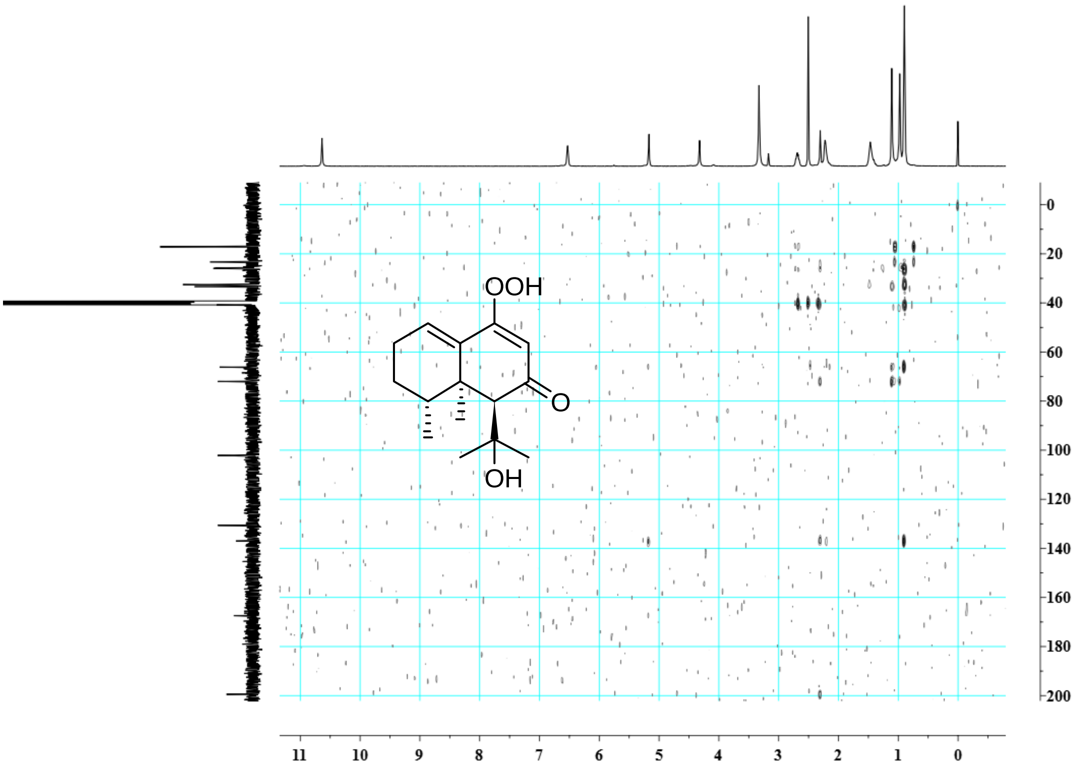

Figure S58. NOESY spectrum of 7-oxonardosinoperoxide (**24**)

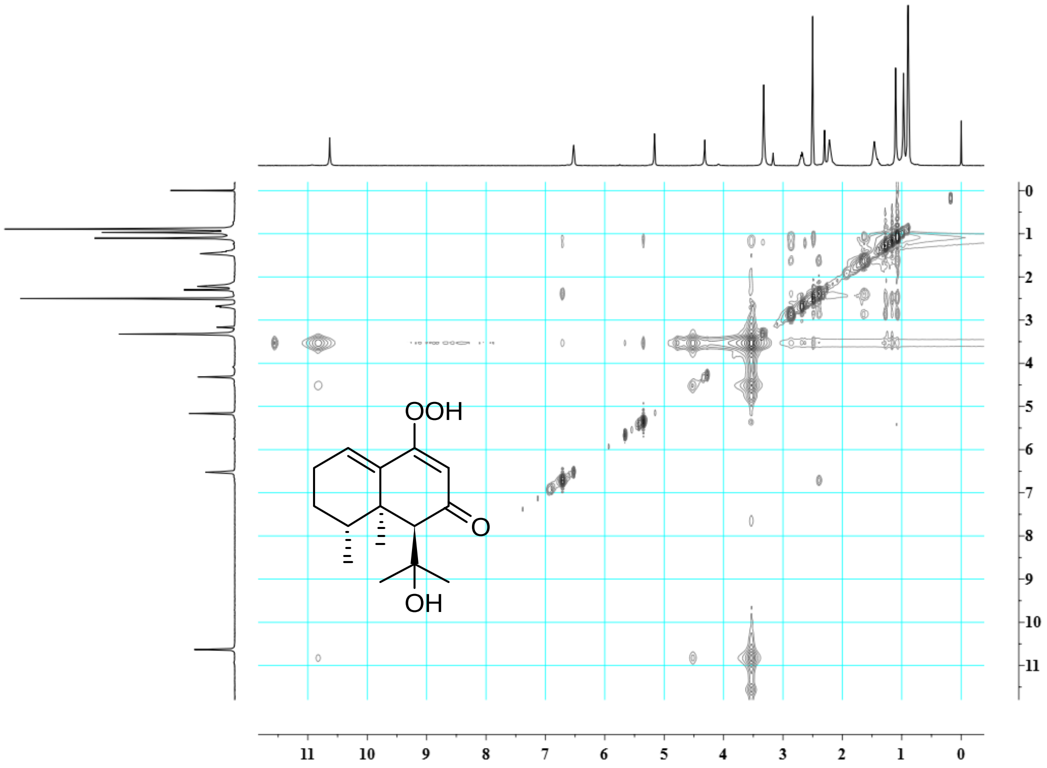

Figure S59. (+)-HRESIMS of 2-oxokanshone A (**26**)

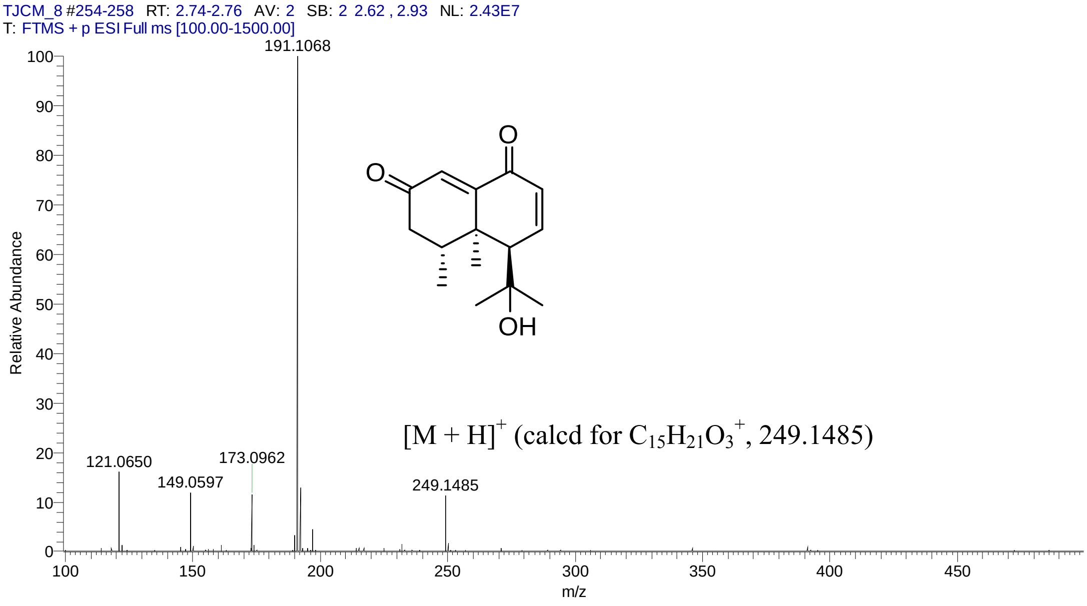

Figure S60. ^{1}H NMR (400.13 MHz, $CDCl_3$) spectrum of 2-oxokanshone A (**26**)

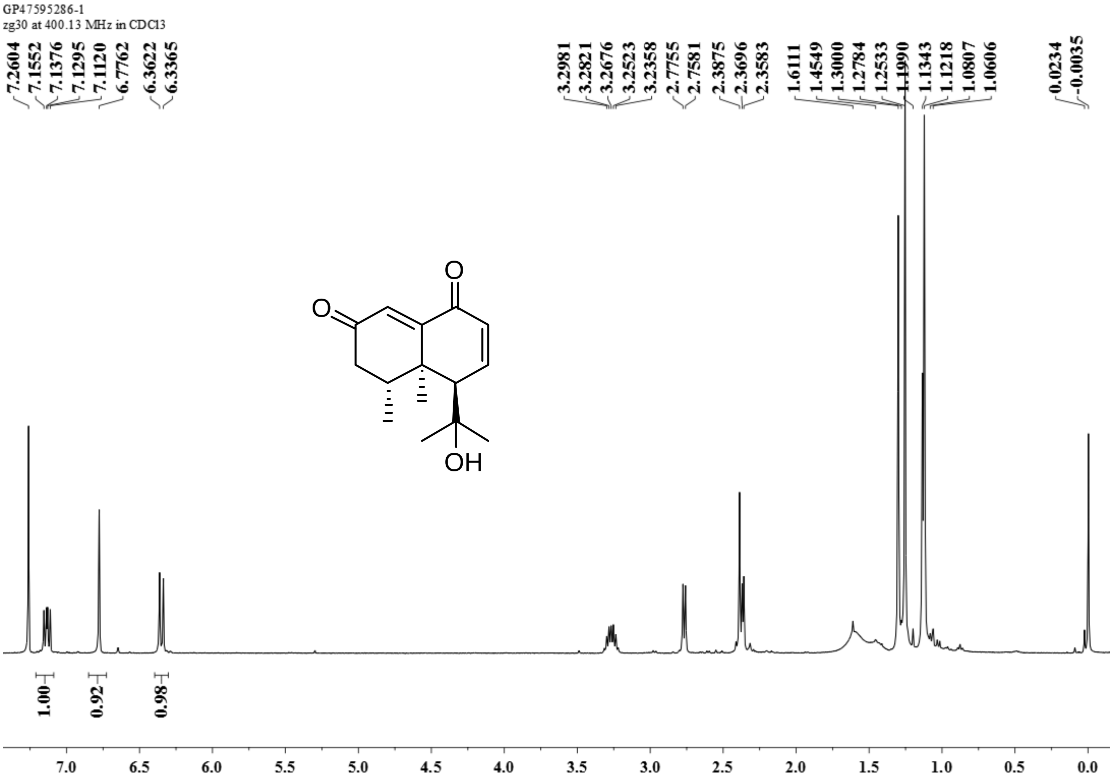

Figure S61. ^{13}C NMR (100.61 MHz, $CDCl_3$) spectrum of 2-oxokanshone A (**26**)

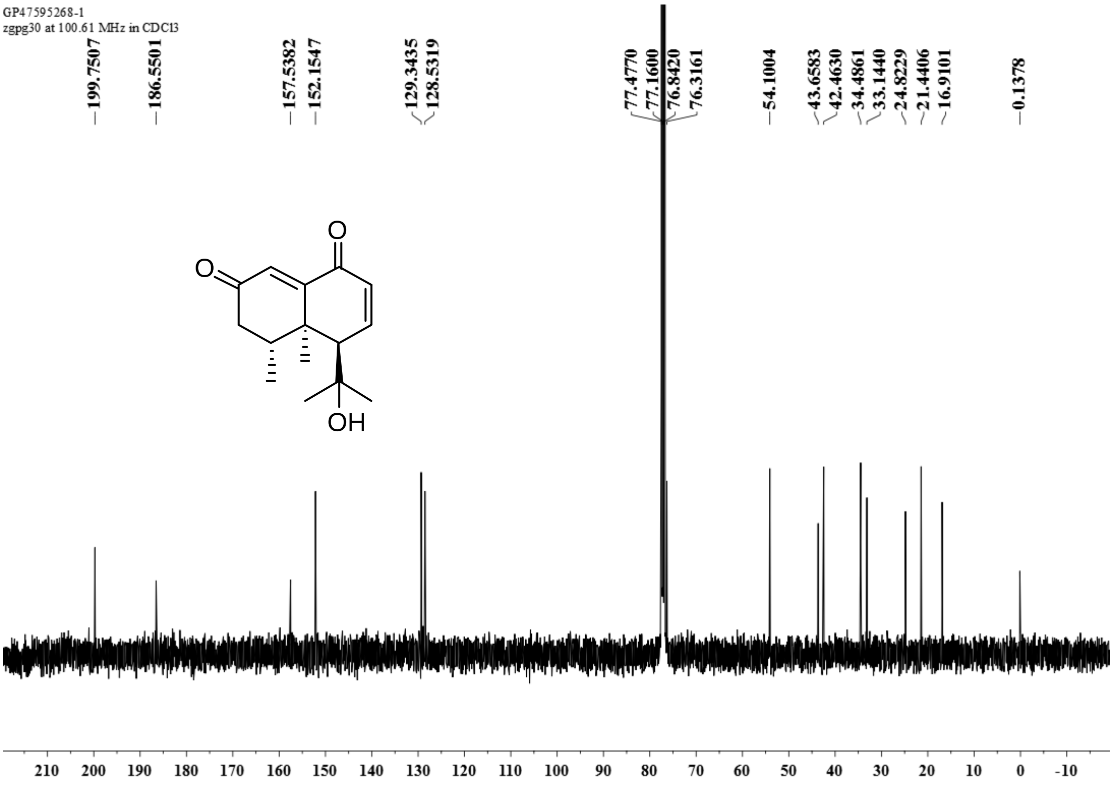

Figure S62. HSQC spectrum of 2-oxokanshone A (**26**)

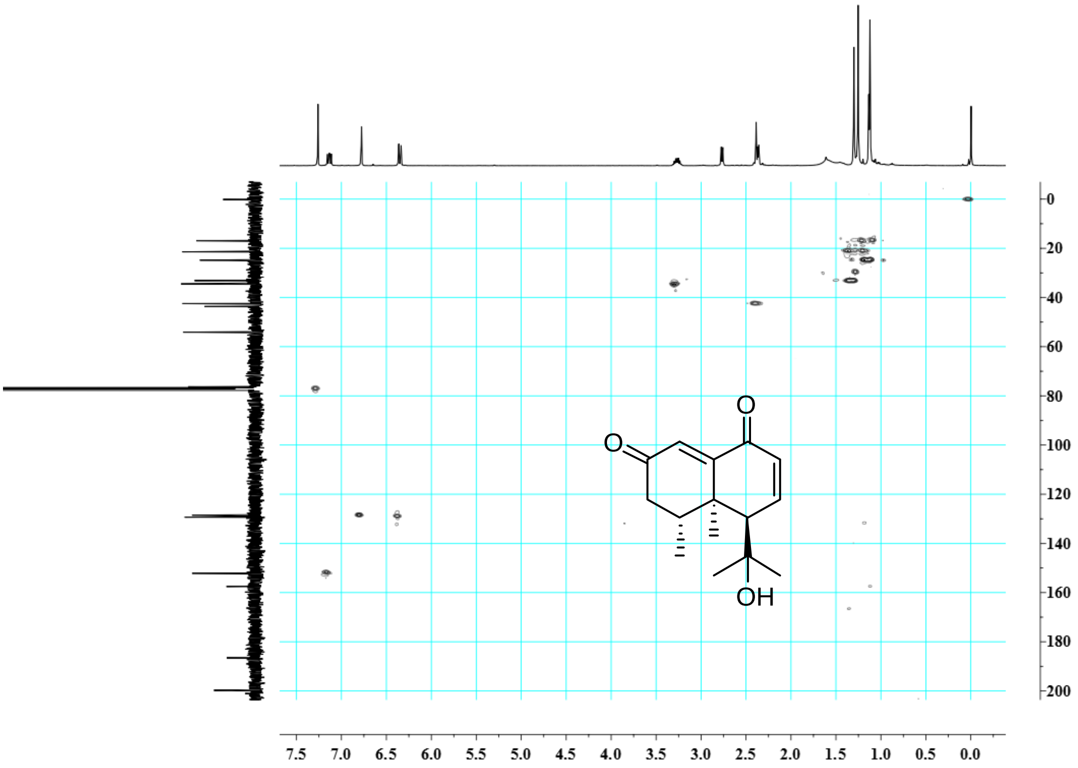

Figure S63. HMBC spectrum of 2-oxokanshone A (**26**)

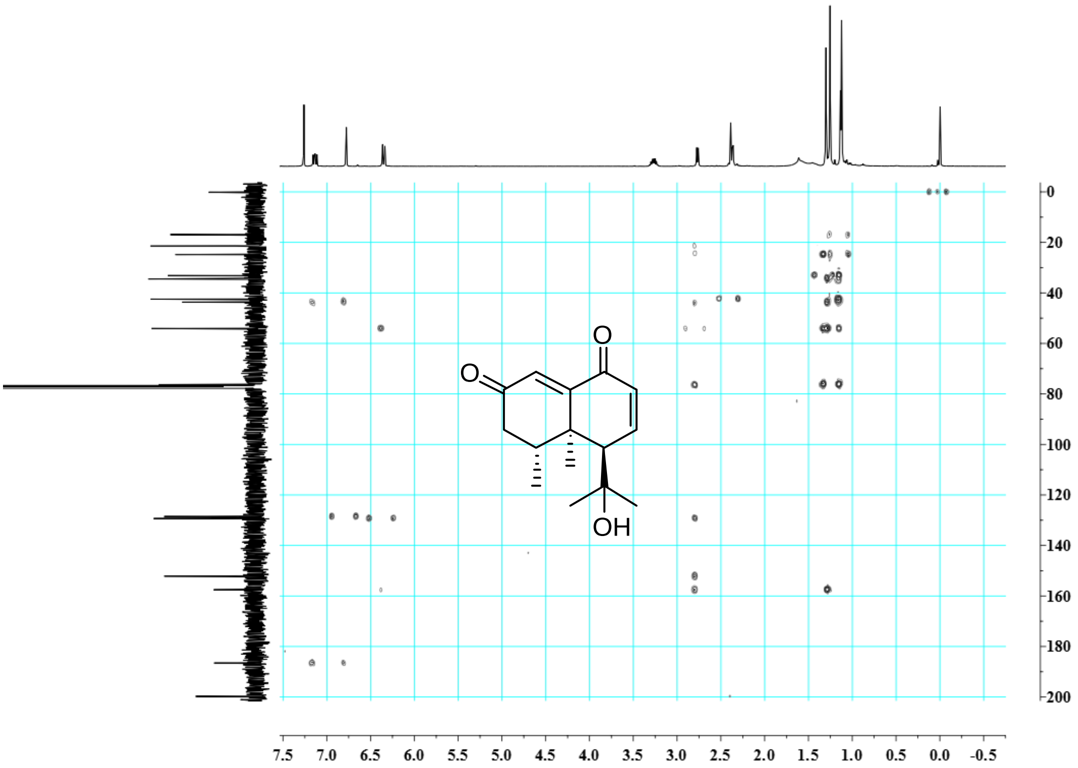

Figure S64. NOESY spectrum of 2-oxokanshone A (**26**)

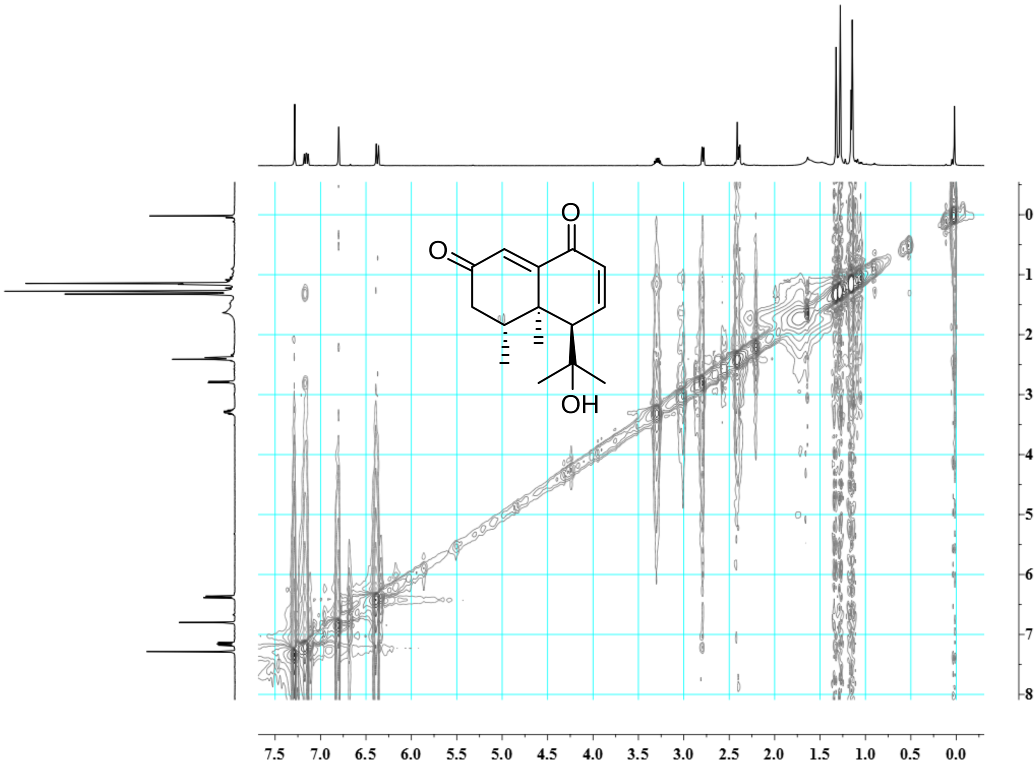

Figure S65. (+)-HRESIMS of epoxynardosinanone H (**30**)

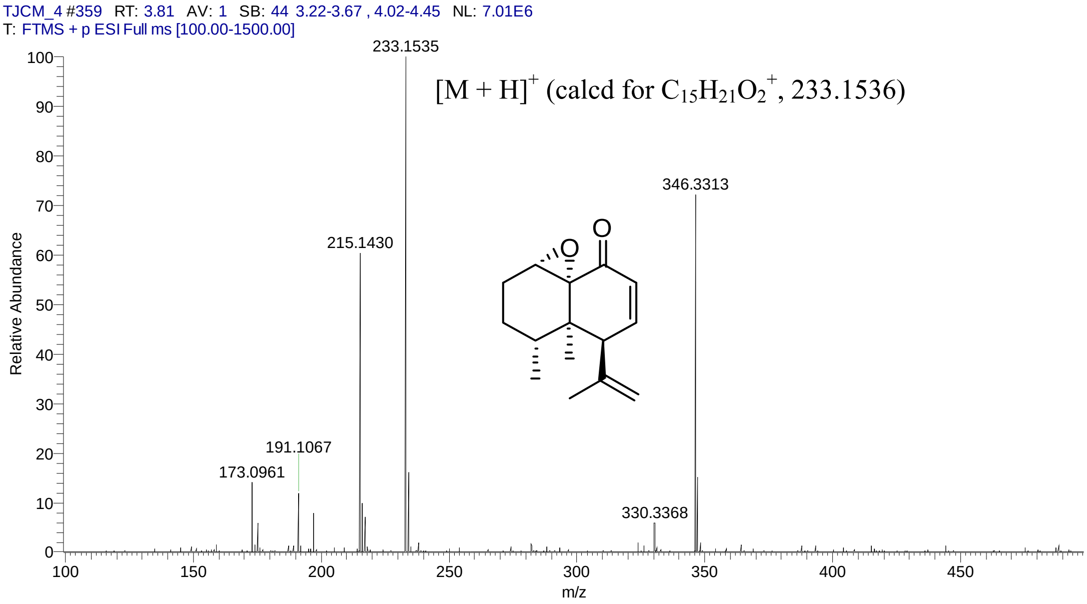

Figure S66. ^{1}H NMR (400.13 MHz, $CDCl_3$) spectrum of epoxynardosinanone H (**30**)

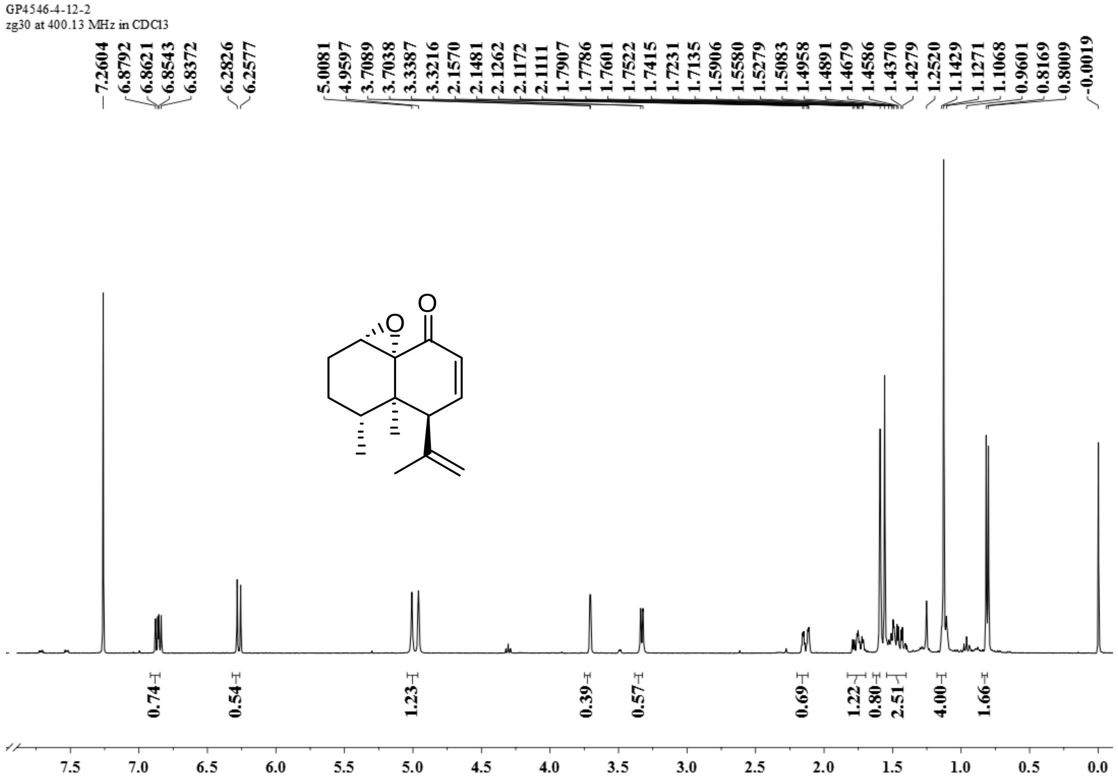

Figure S67. ^{13}C NMR (100.61 MHz, $CDCl_3$) spectrum of epoxynardosinanone H (**30**)

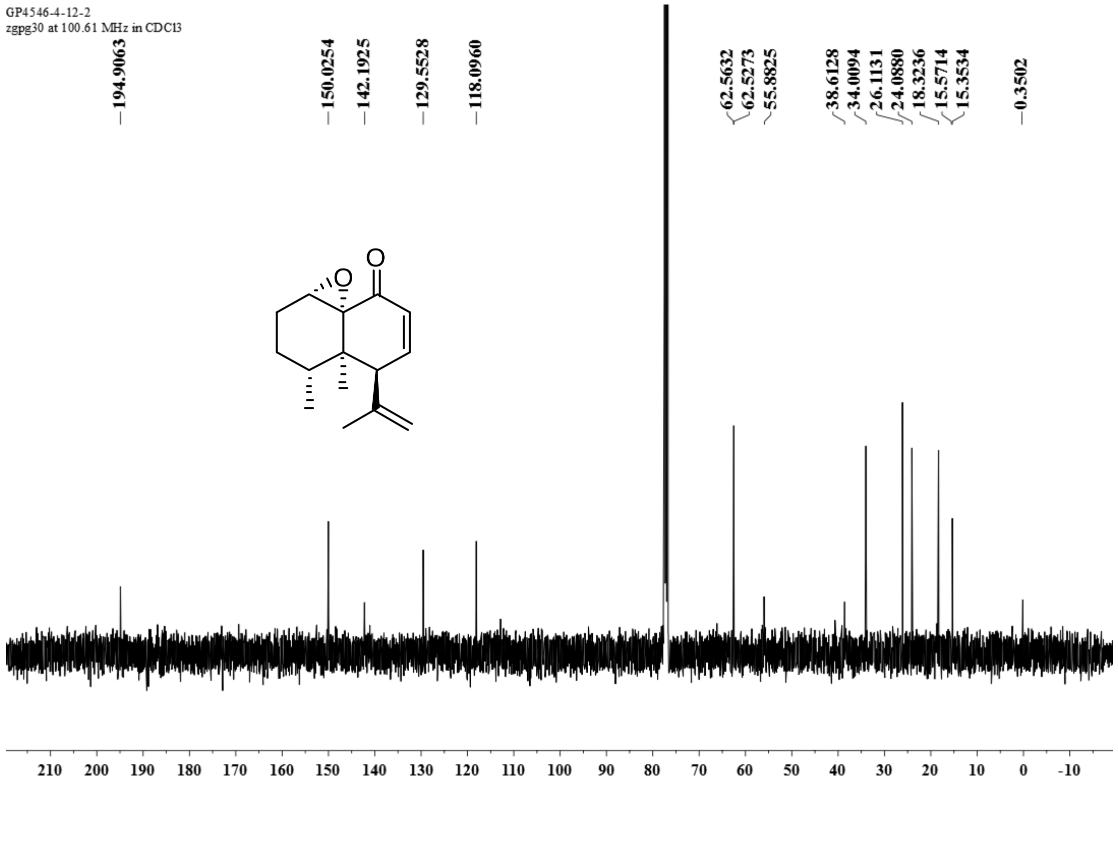

Figure S68. DEPT(135°) spectrum of epoxynardosinanone H (**30**)

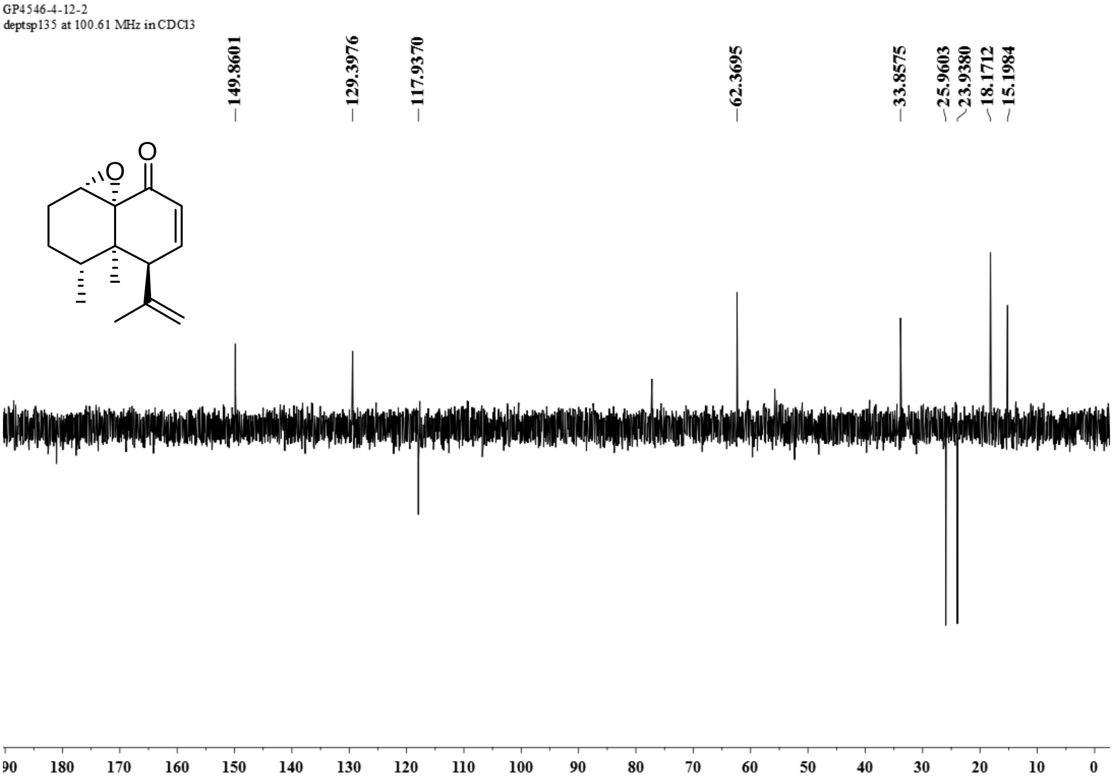

Figure S69. HSQC spectrum of epoxynardosinanone H (**30**)

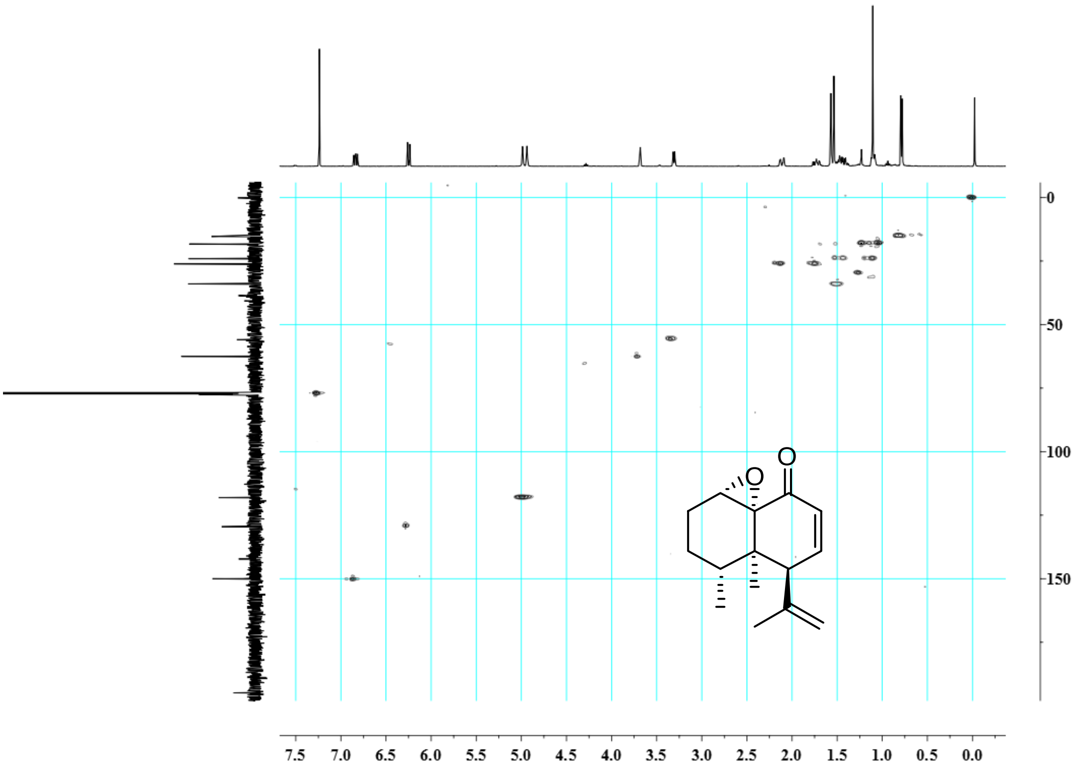

Figure S70. HMBC spectrum of epoxynardosinanone H (**30**)

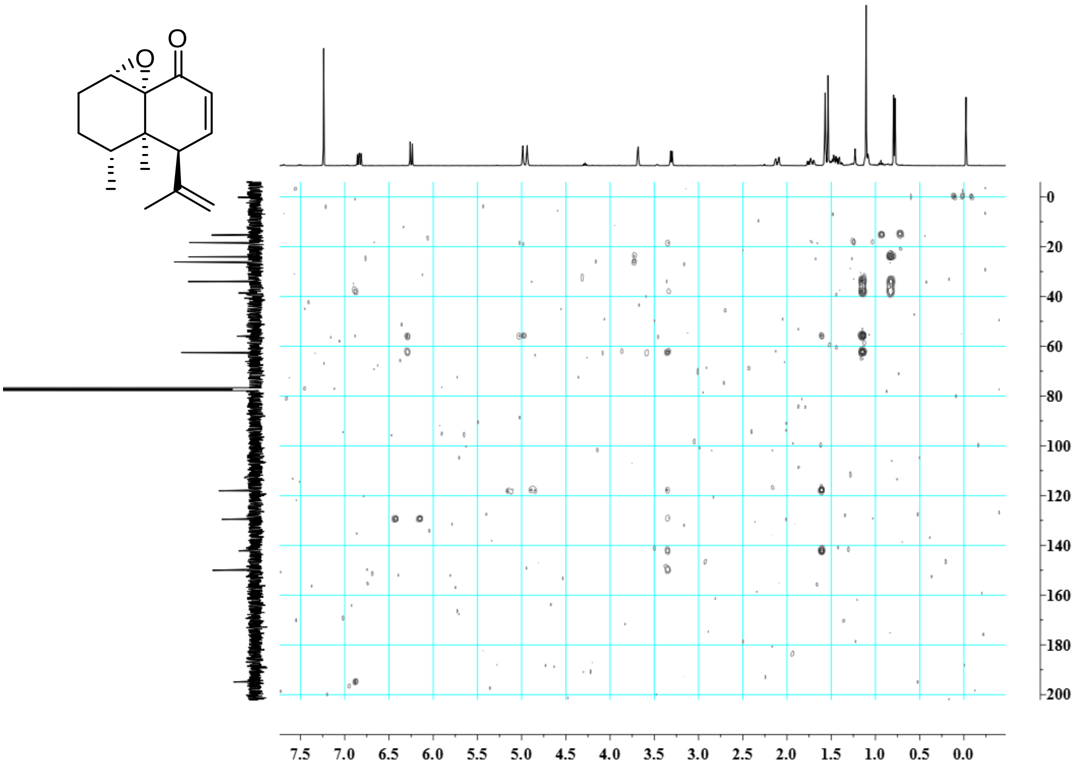

Figure S71. (+)-HRESIMS of nardosinanone M (**35**)

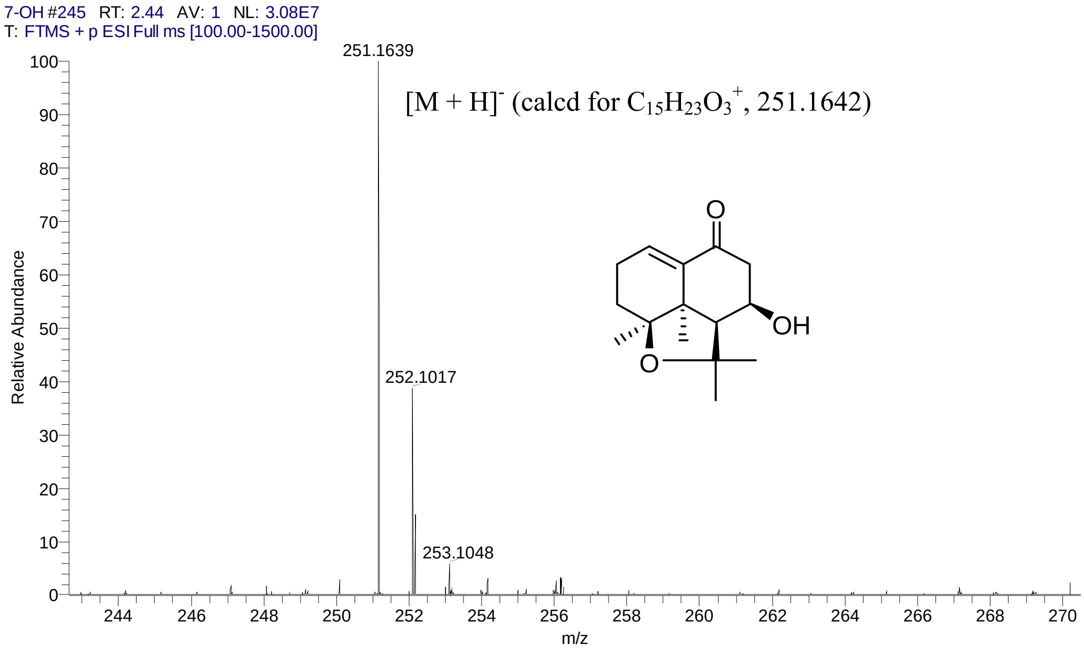

Figure S72. ^{1}H NMR (400.13 MHz, $CDCl_3$) spectrum of nardosinanone M (**35**)

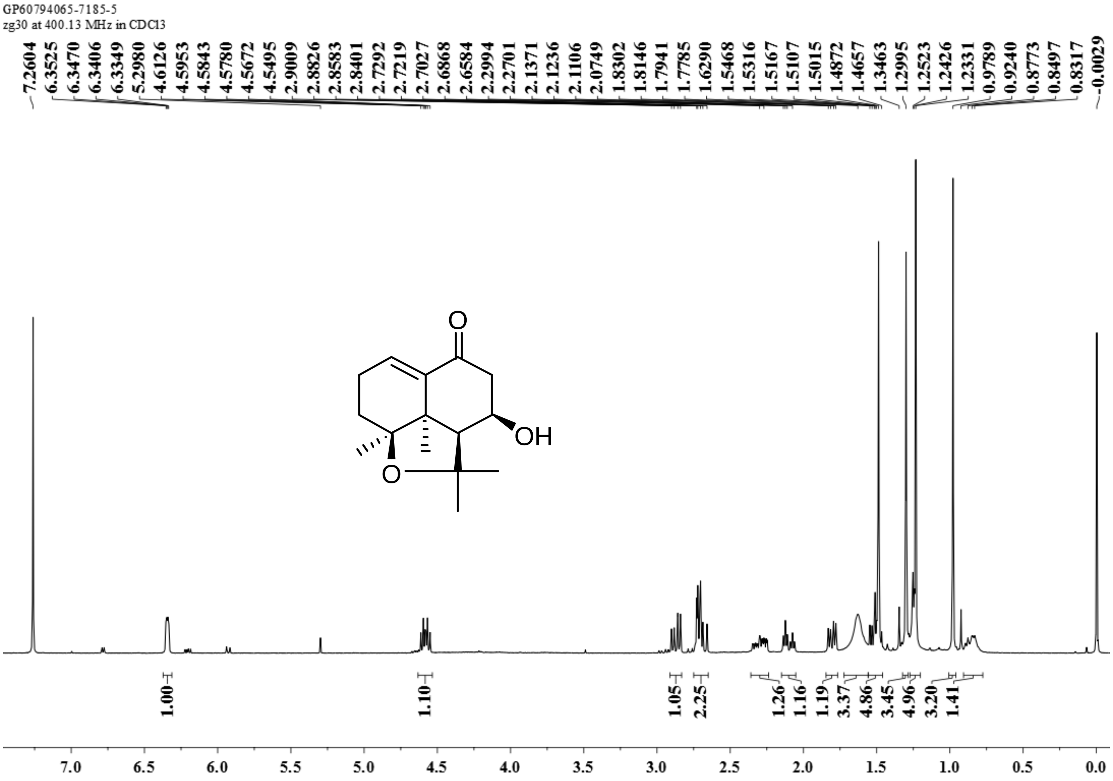

Figure S73. ^{13}C NMR (100.61 MHz, $CDCl_3$) spectrum of nardosinanone M (**35**)

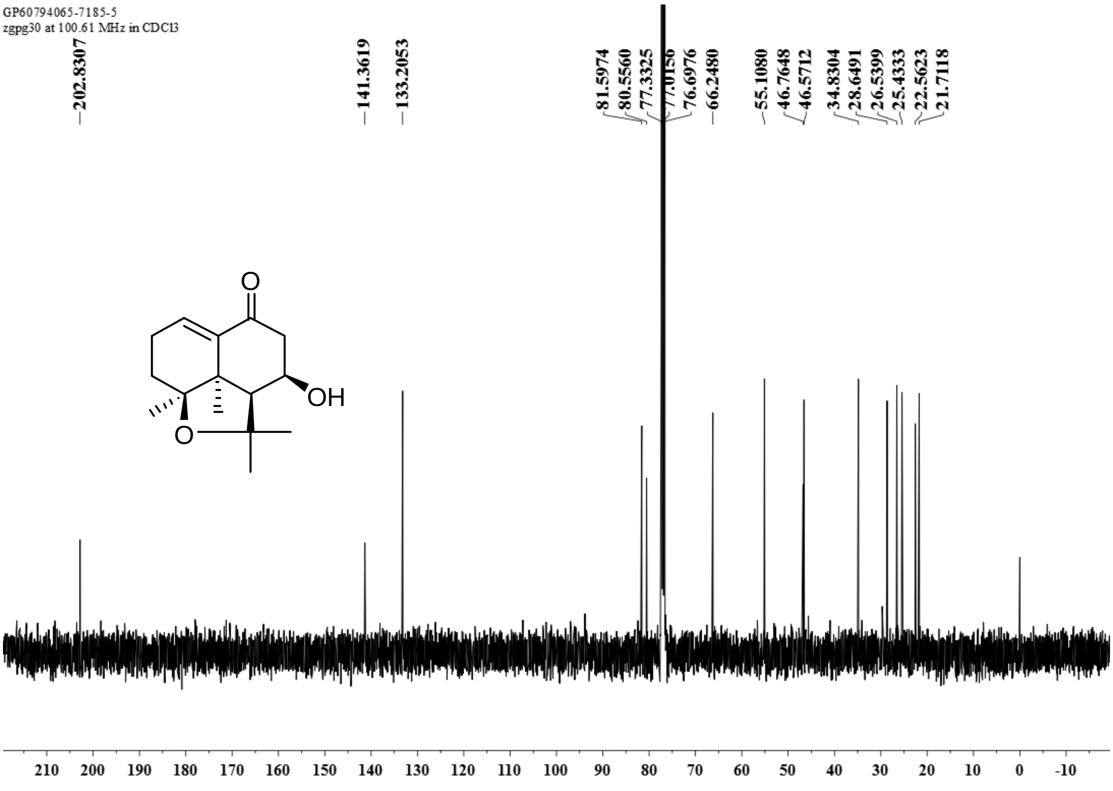

Figure S74. HSQC spectrum of nardosinanone M (**35**)

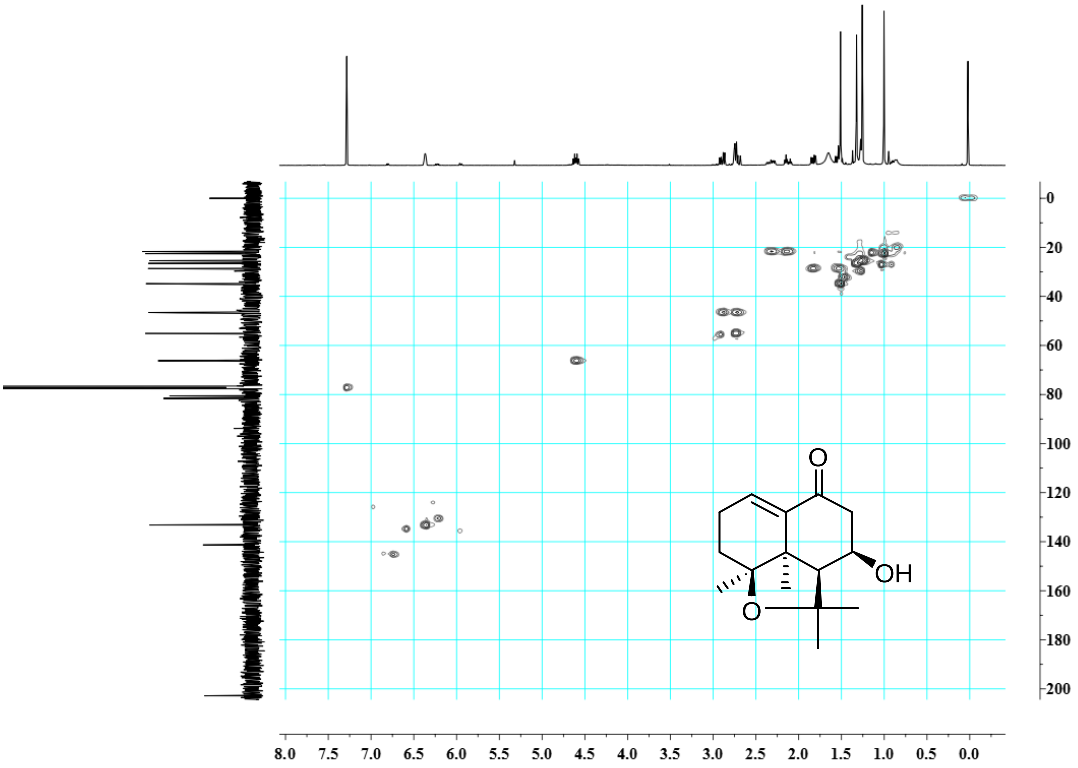

Figure S75. HMBC spectrum of nardosinanone M (**35**)

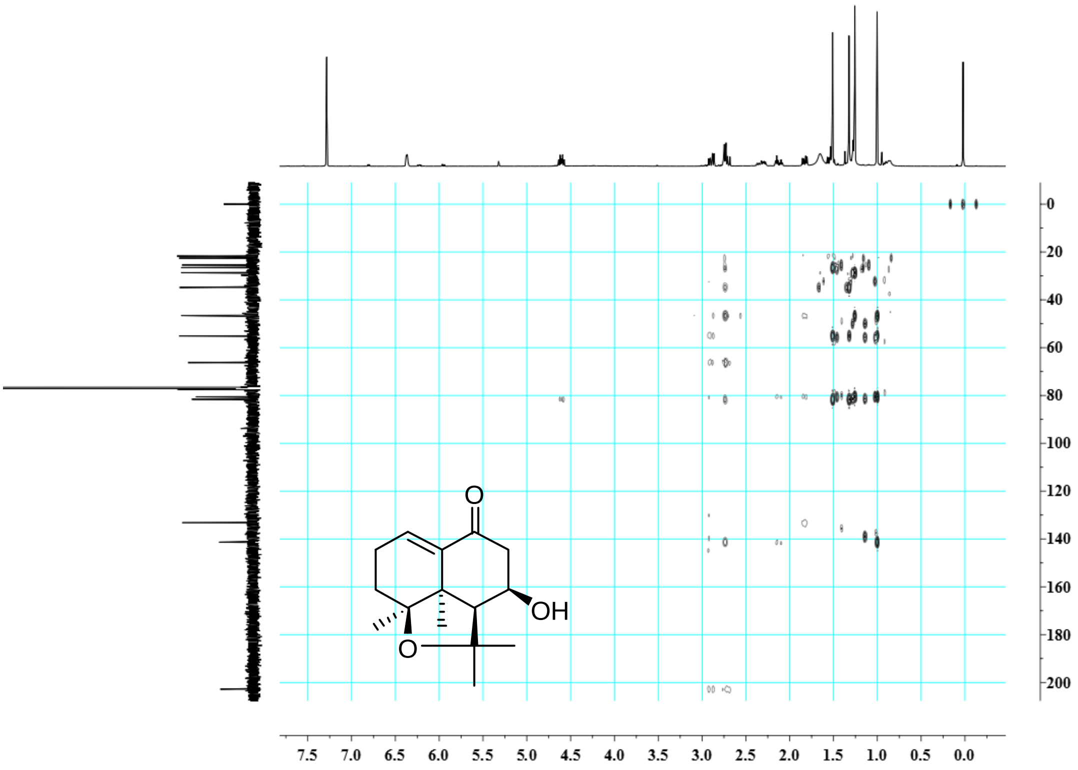

Figure S76. NOESY spectrum of nardosinanone M (**35**)

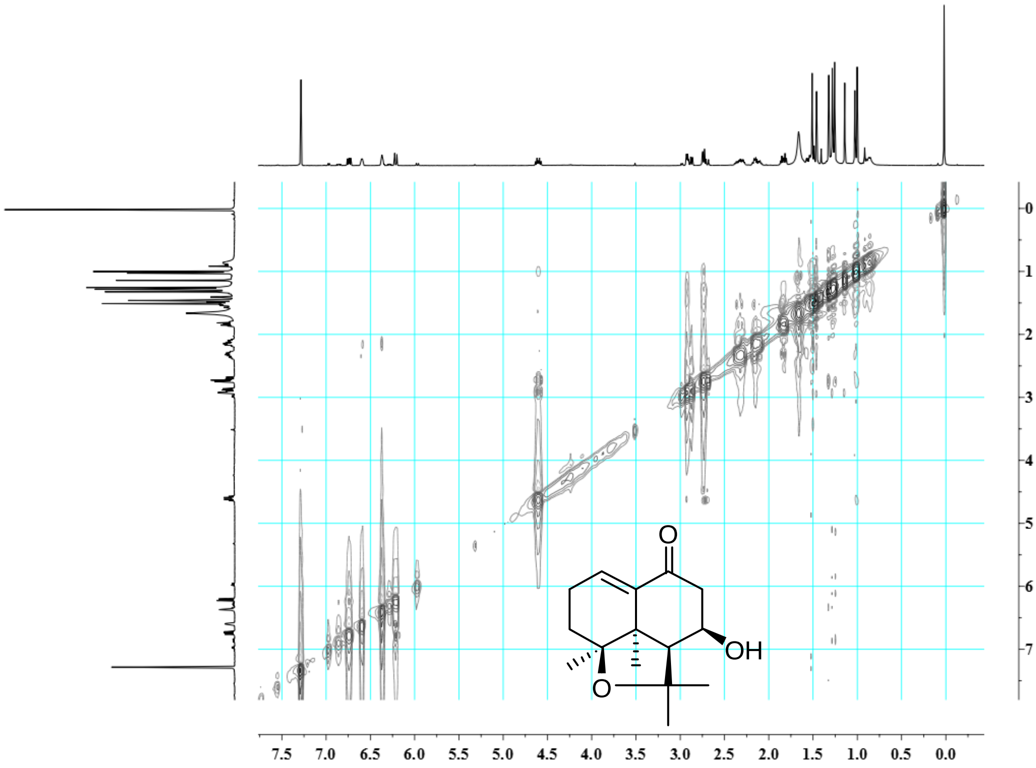

Figure S77. UV and CD spectra of compounds **1**, **3**, **6**, **7**, **11**, **14**, **18**, **19**, **22**, **23**, **24**, **26**, **30** and **35**

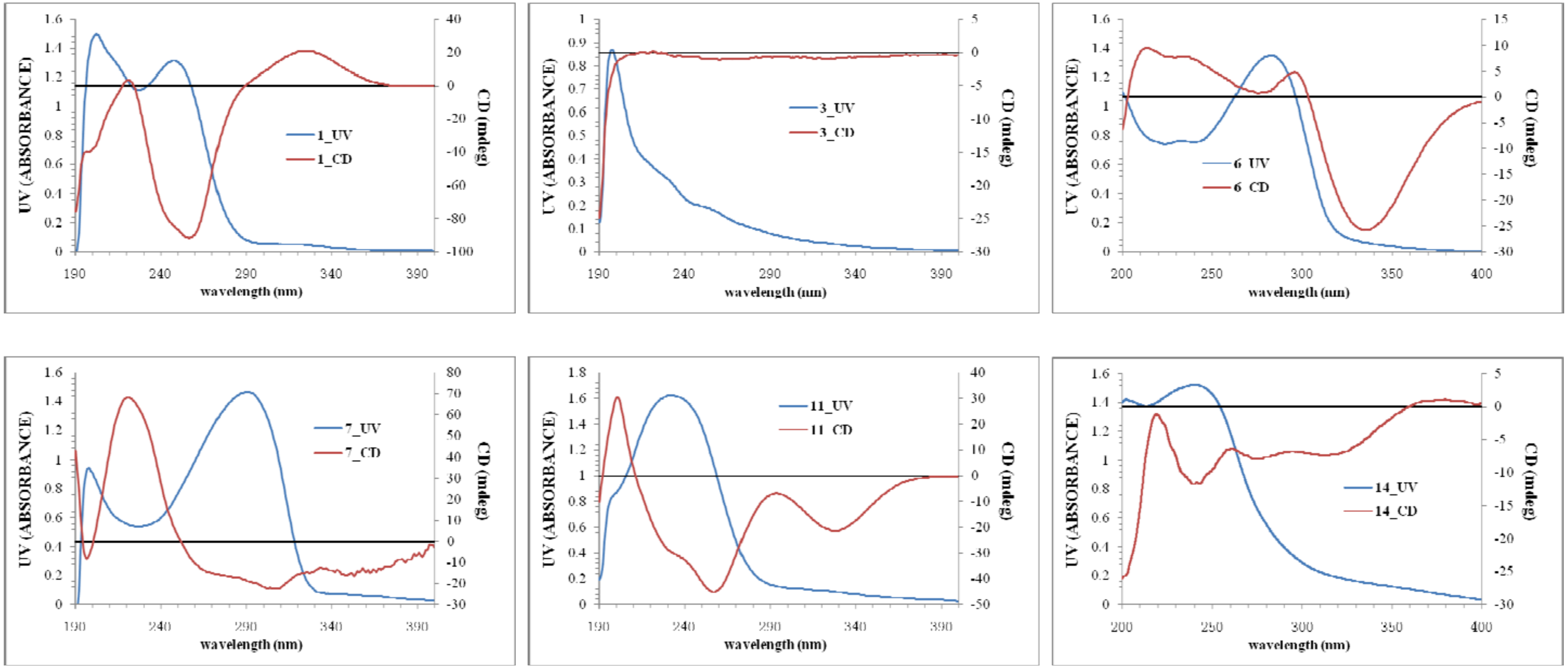

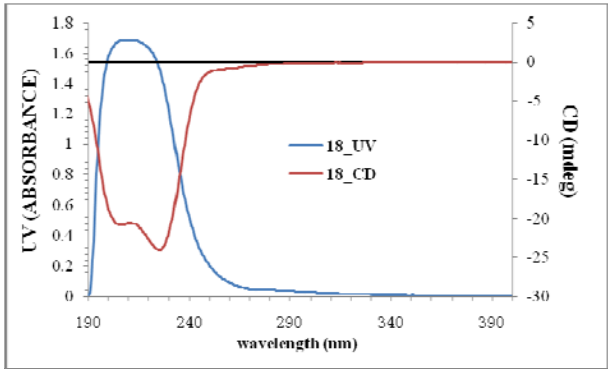
UV (ABSORBANCE)
CD (mdeg)
wavelength (nm)
18_UV
18_CD

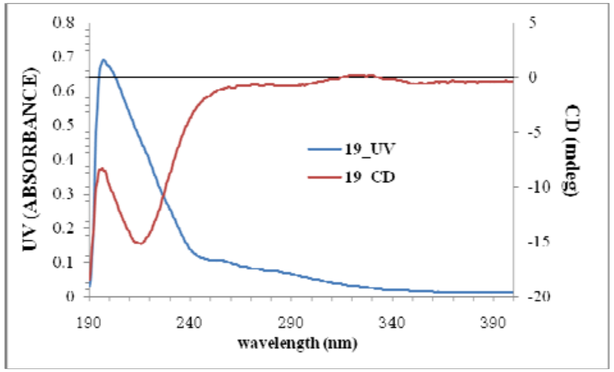
UV (ABSORBANCE)
CD (mdeg)
wavelength (nm)
19_UV
19_CD

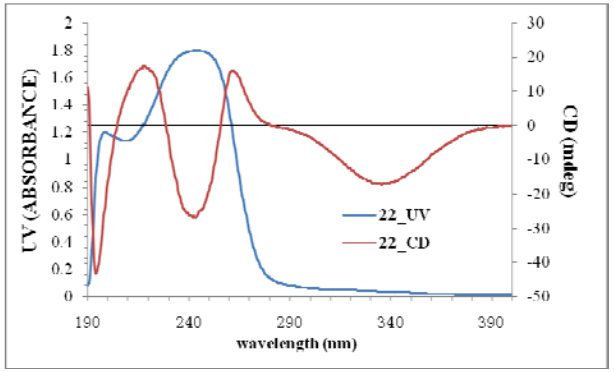
UV (ABSORBANCE)
CD (mdeg)
wavelength (nm)
22_UV
22_CD

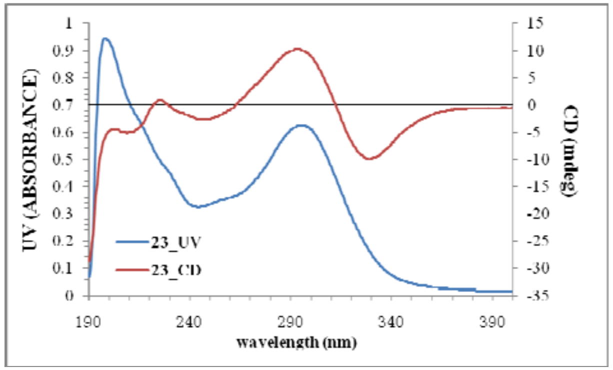
UV (ABSORBANCE)
CD (mdeg)
wavelength (nm)
23_UV
23_CD

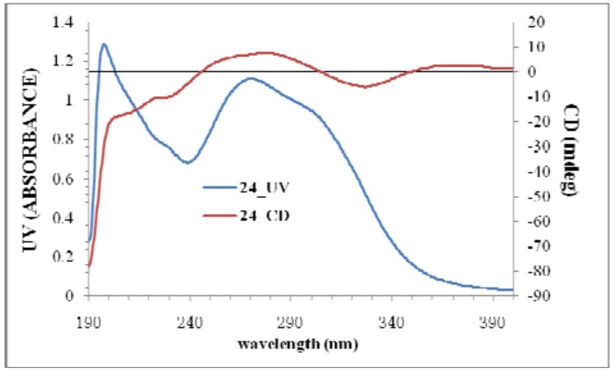
UV (ABSORBANCE)
CD (mdeg)
wavelength (nm)
24_UV
24_CD

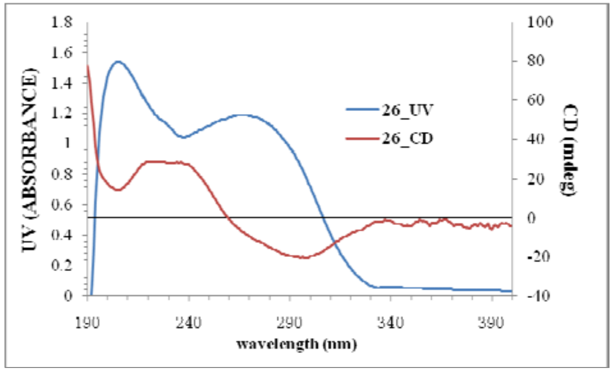
UV (ABSORBANCE)
CD (mdeg)
wavelength (nm)
26_UV
26_CD

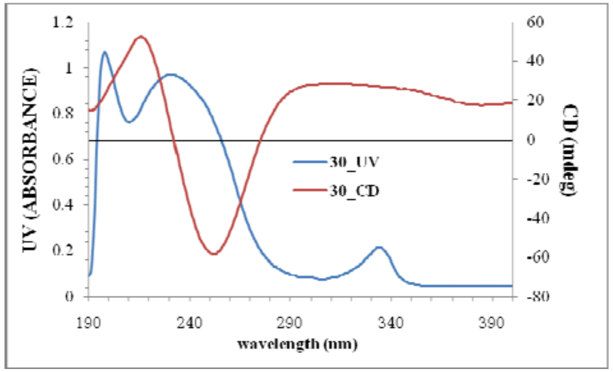
UV (ABSORBANCE)
CD (mdeg)
30_UV
30_CD
wavelength (nm)
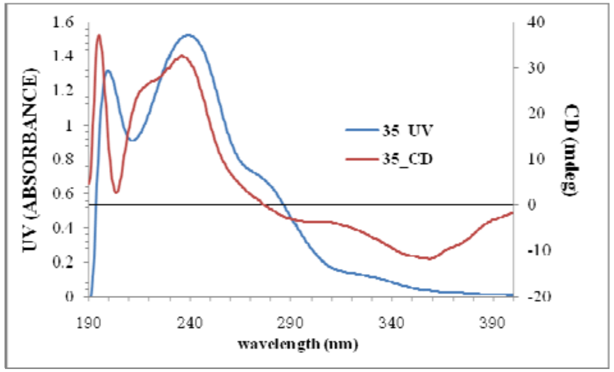
UV (ABSORBANCE)
CD (mdeg)
35_UV
35_CD
wavelength (nm)

Figure S78. Comparative UV spectra for compounds **1**– **40**

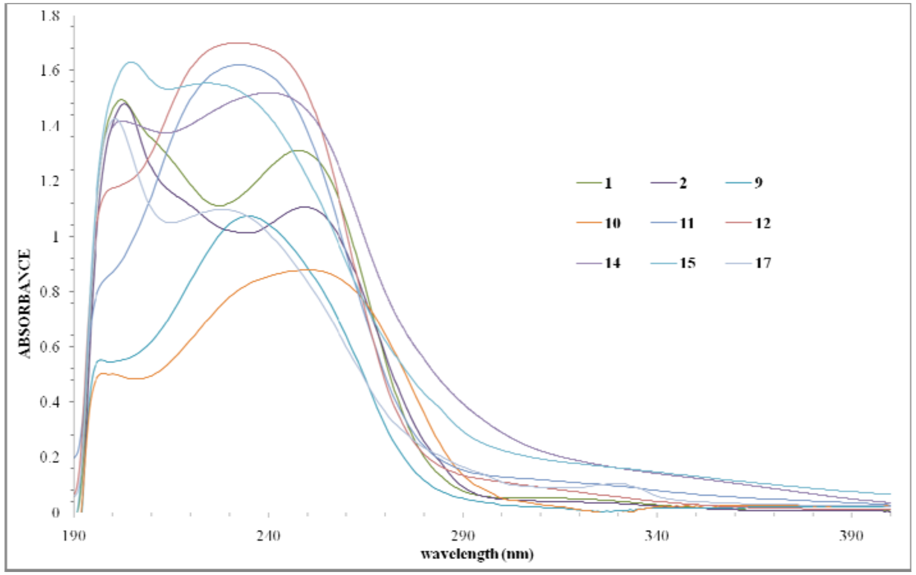

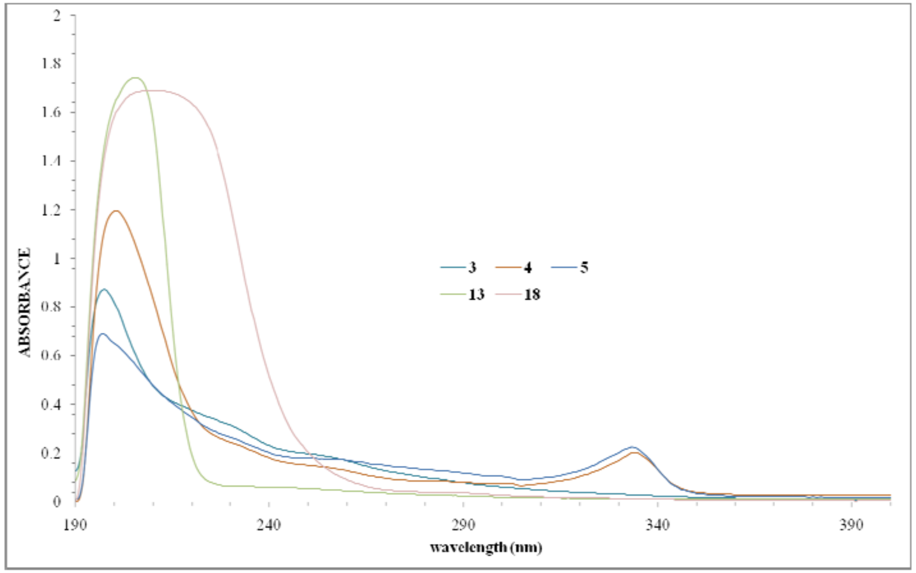

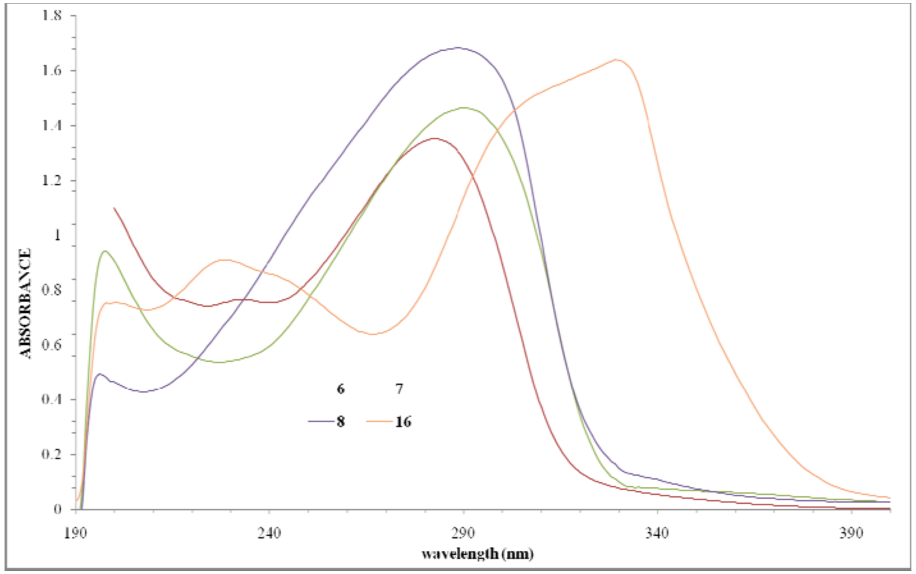
ABSORBANCE
wavelength (nm)
6
7
8
16

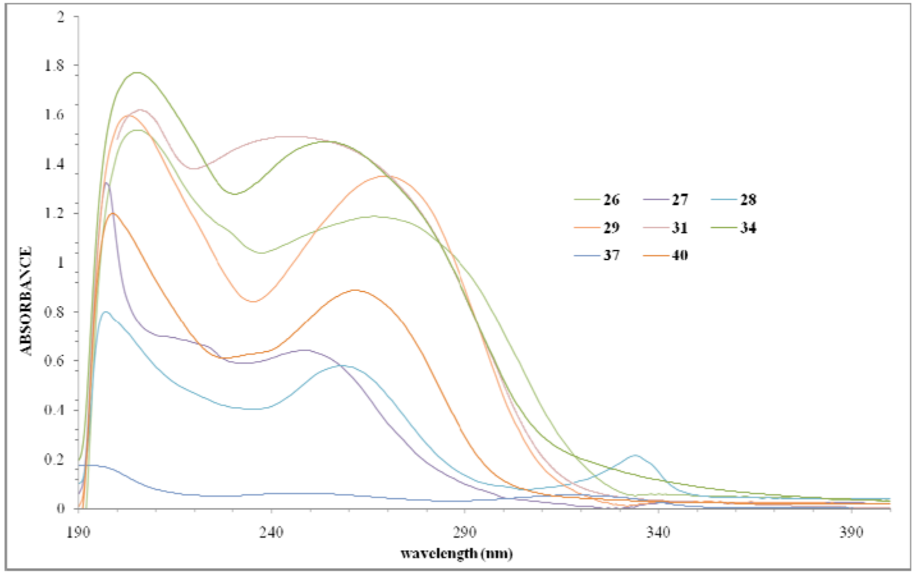
ABSORBANCE
wavelength (nm)
26
27
28
29
31
34
37
40

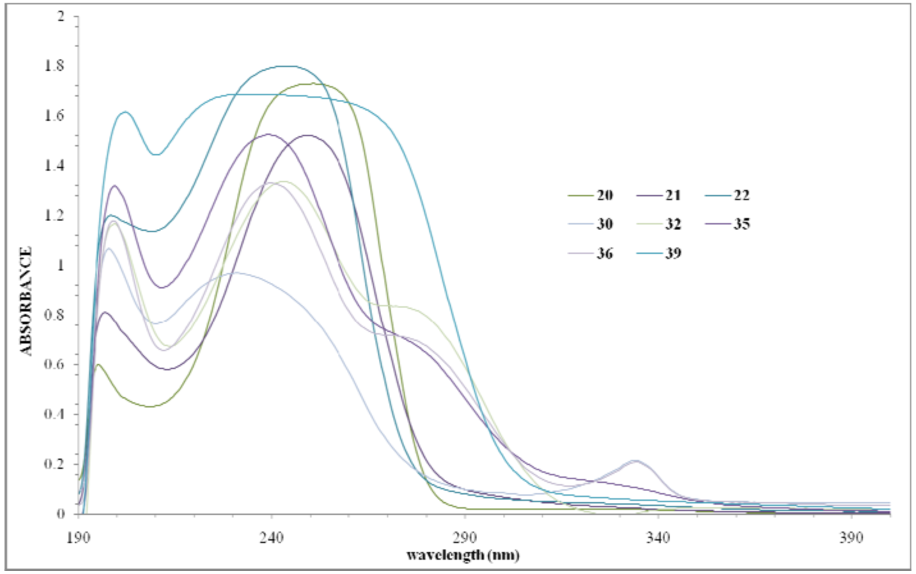
ABSORBANCE
wavelength (nm)
20
21
22
30
32
35
36
39

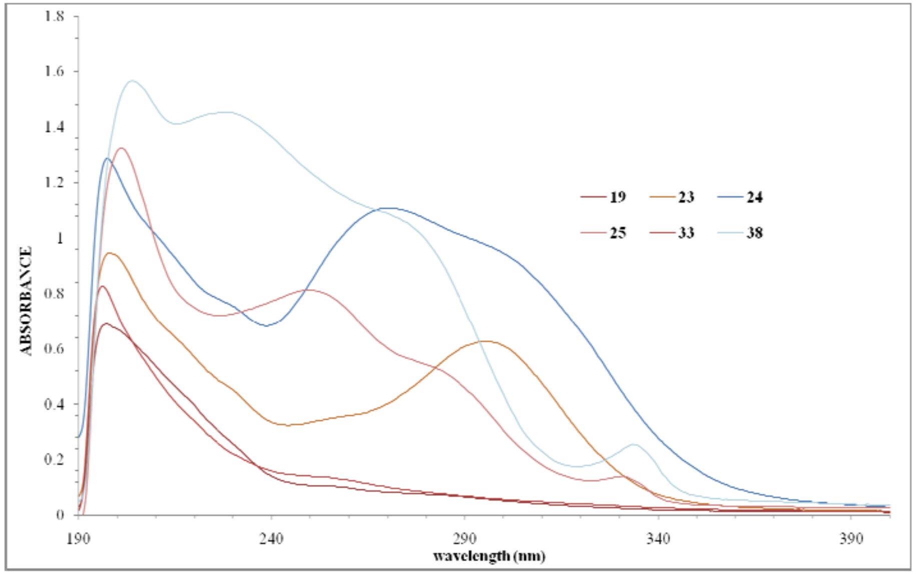
ABSORBANCE
wavelength (nm)
19
23
24
25
33
38

Figure S79. Comparative CD spectra for some aristolane- and nardosinane- types of sesquiterpenoids from NCB

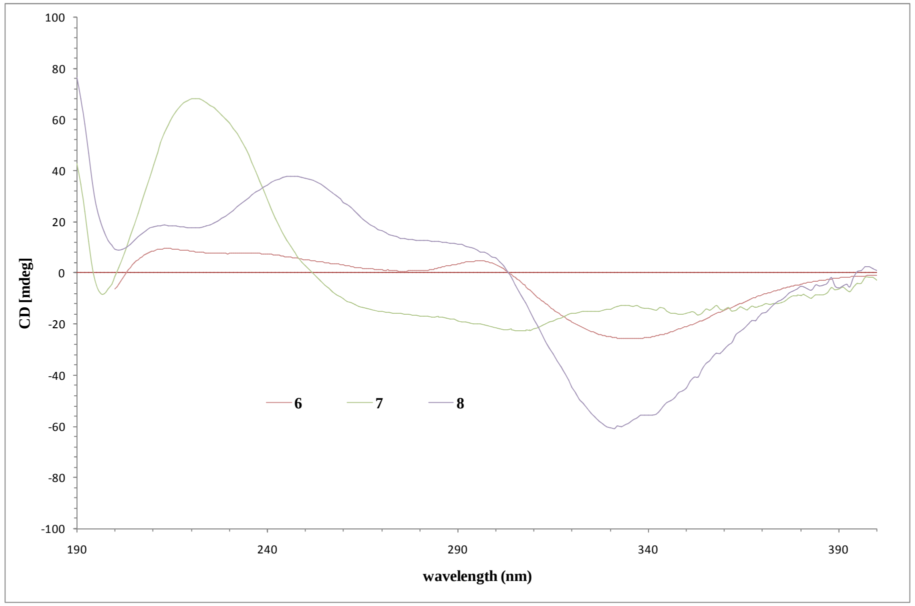

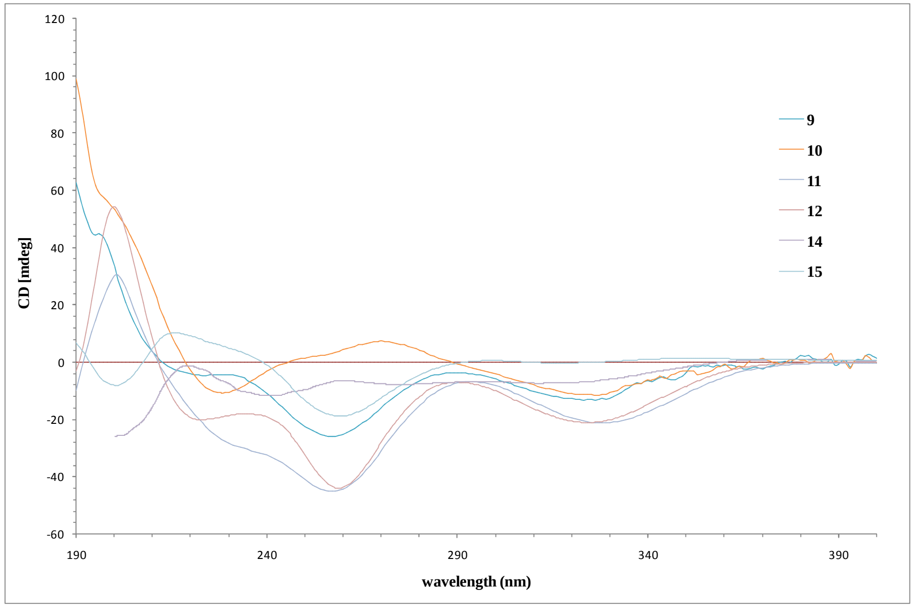

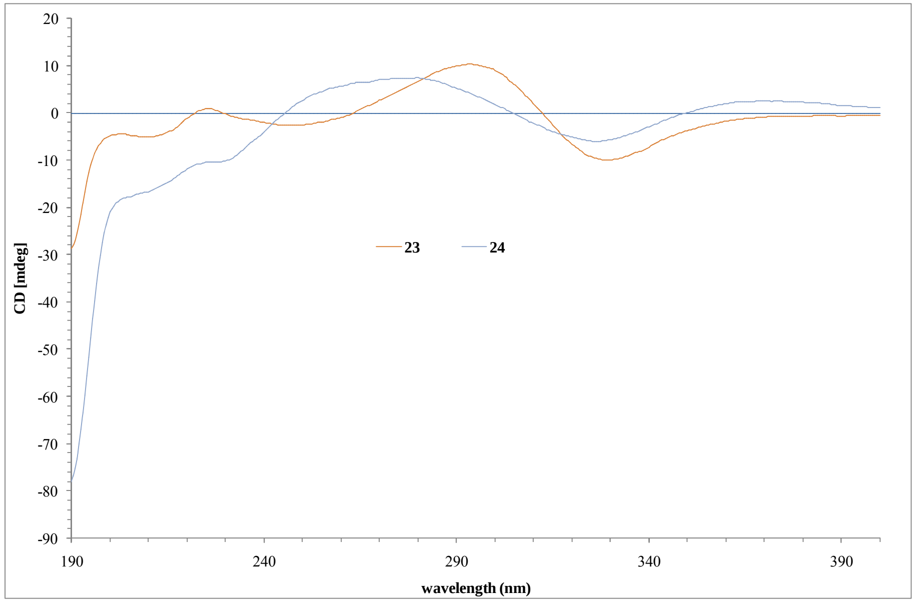

CD [mdeg]
wavelength (nm)
20
10
0
-10
-20
-30
-40
-50
-60
-70
-80
-90
190
240
290
340
390
23
24

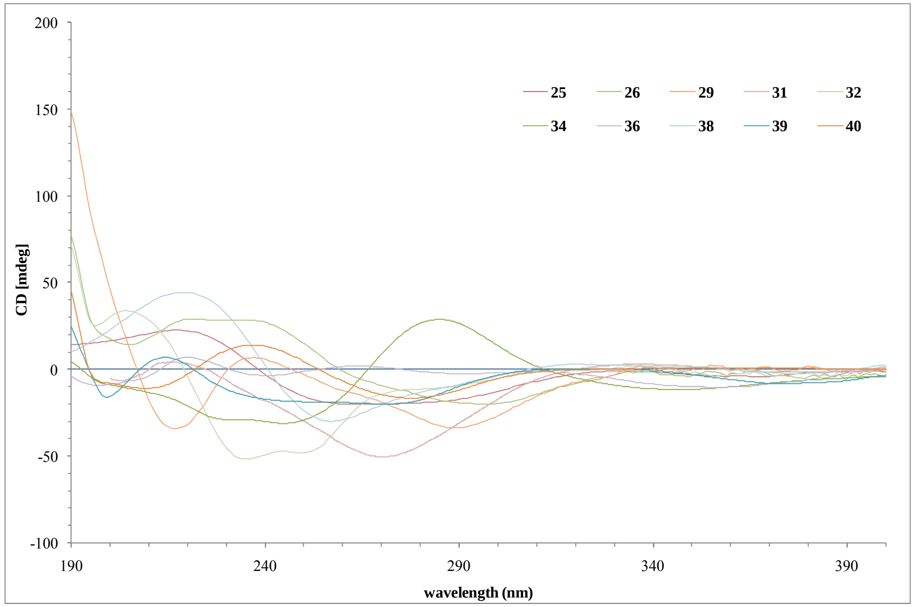

CD [mdeg]
wavelength (nm)
200
150
100
50
0
-50
-100
190
240
290
340
390
25
26
29
31
32
34
36
38
39
40

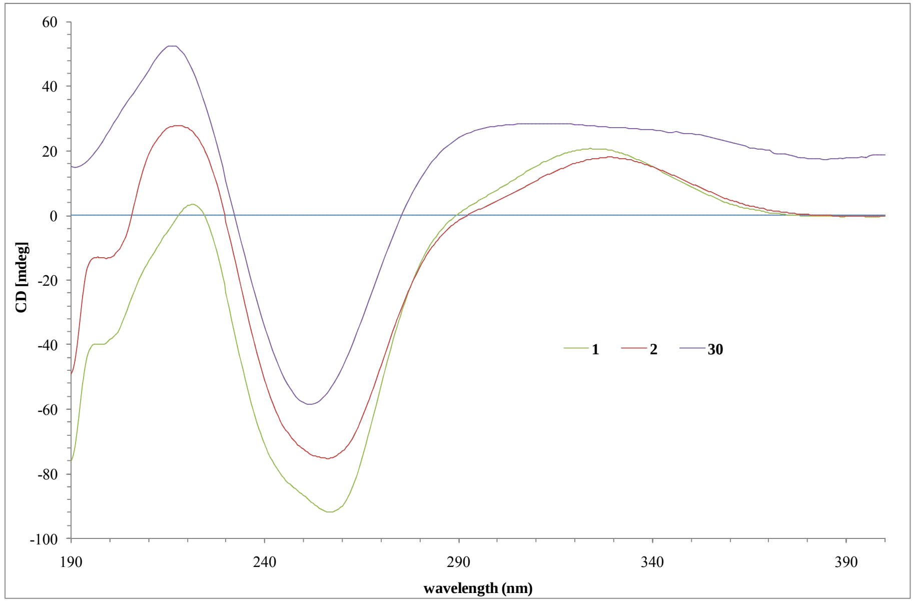

CD [mdeg]
wavelength (nm)
1
2
30

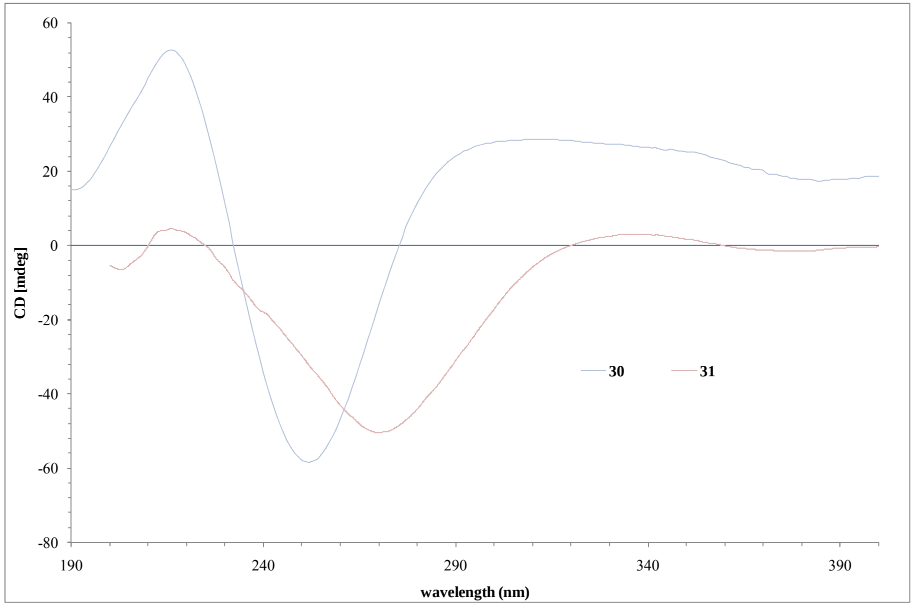

CD [mdeg]
wavelength (nm)
30
31

Figure S80. Calculated and experimental UV and ECD spectra of compound **11**

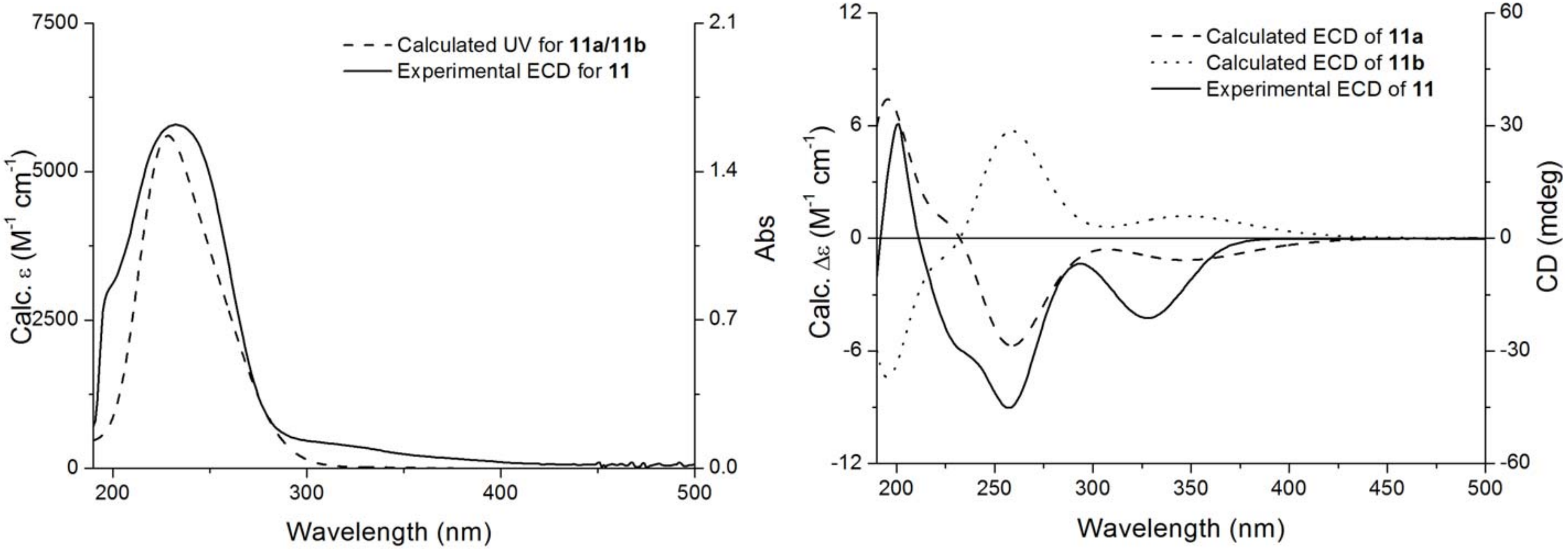

Figure S81. Calculated and experimental UV and ECD spectra of compound **18**

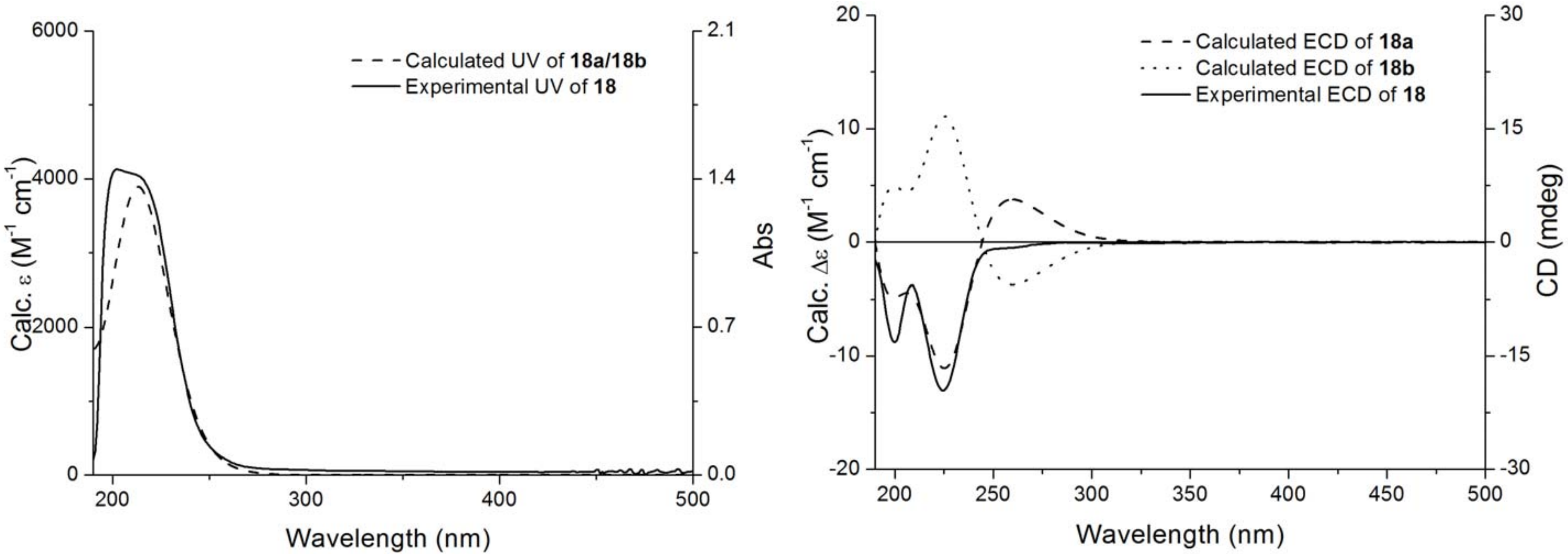

Figure S82. Proposed biosynthetic pathway for aristolane-type sesquiterpenoids from NCB

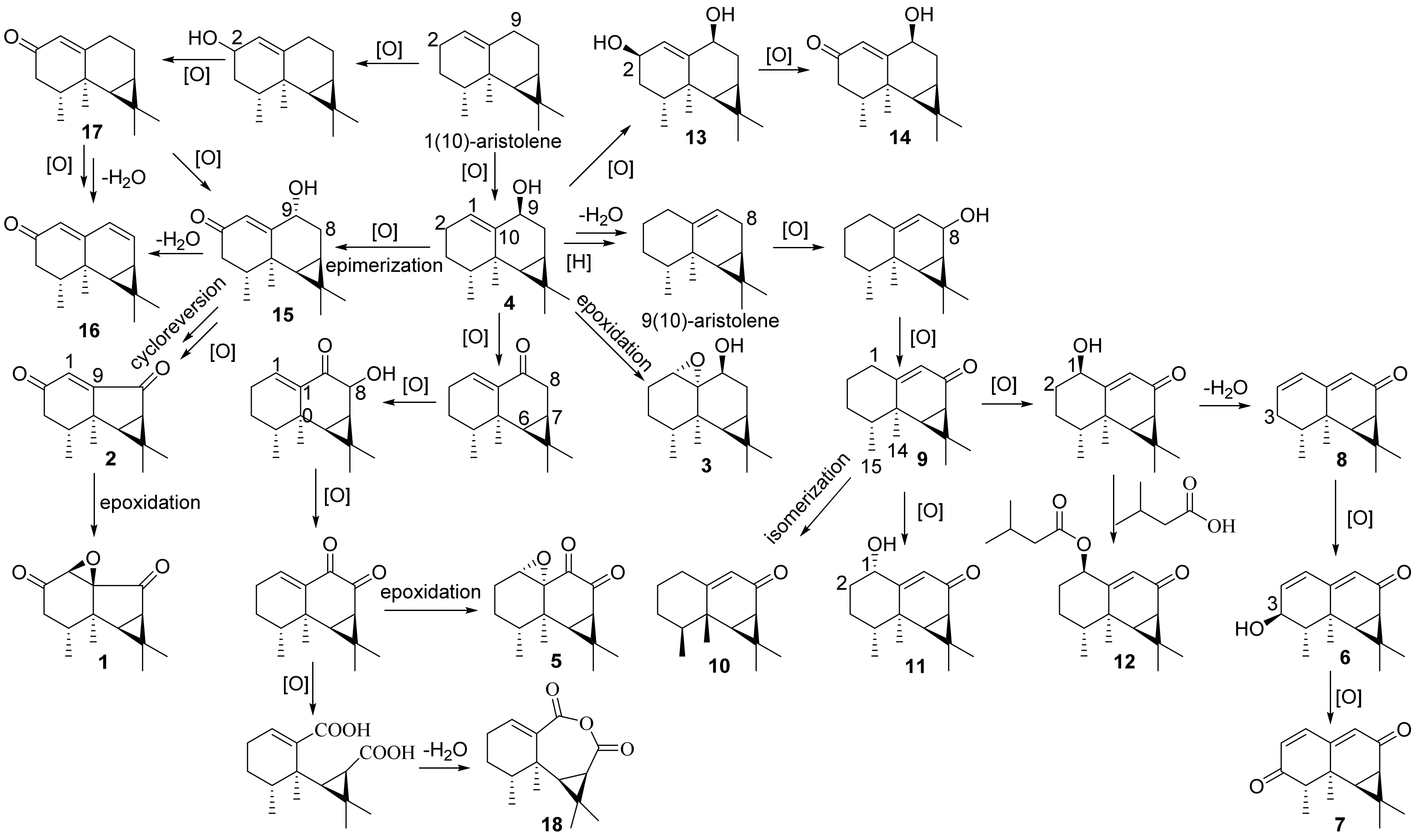

Figure S83. Proposed biosynthetic pathway for nardosinane-type sesquiterpenoids from NCB

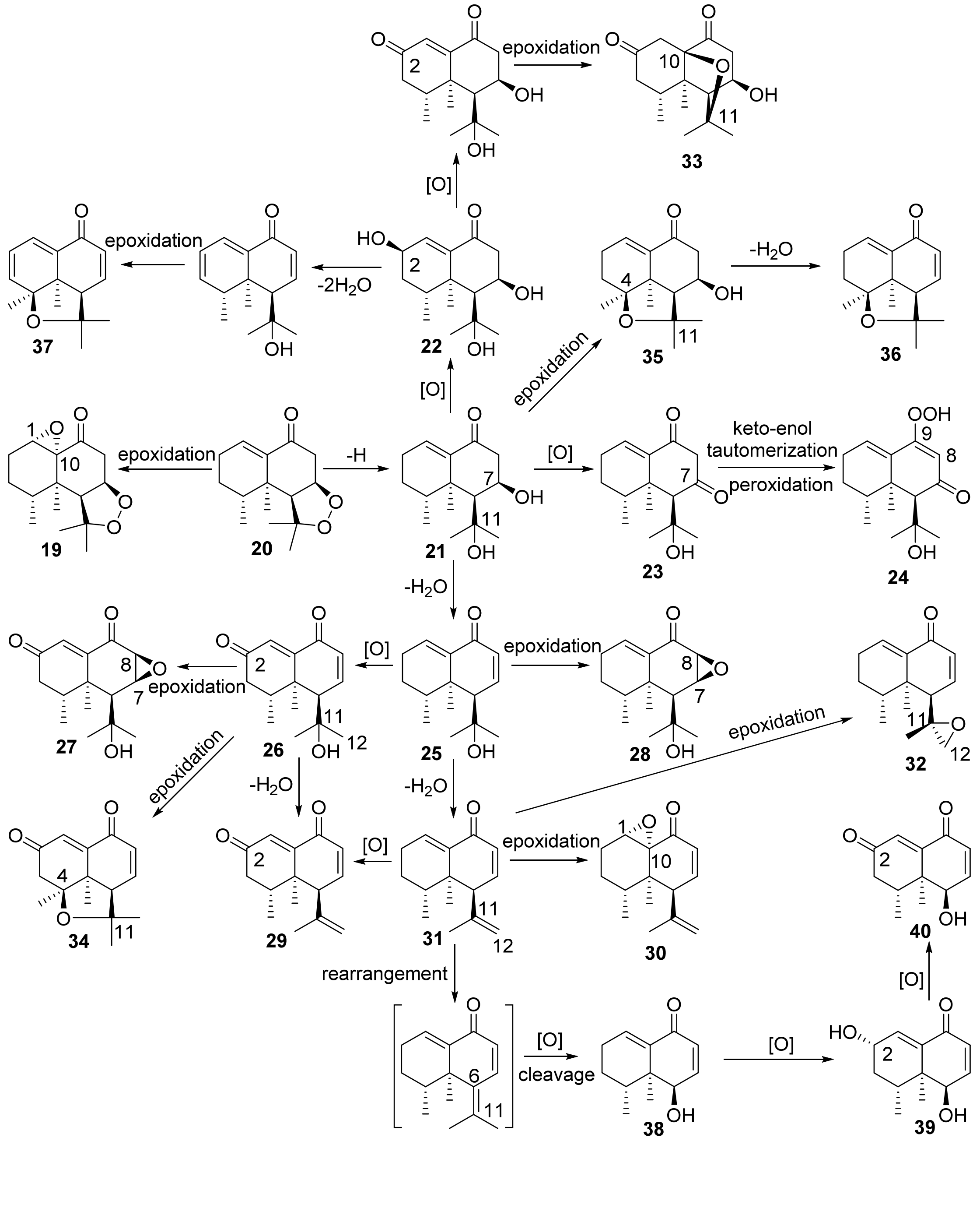

Table S1. The optimized conformer of compound **11**

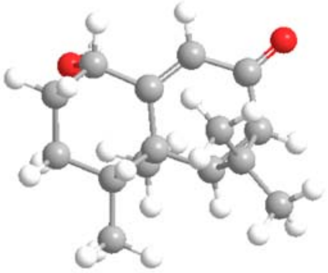

Table S2. The optimized conformers of compound **18**

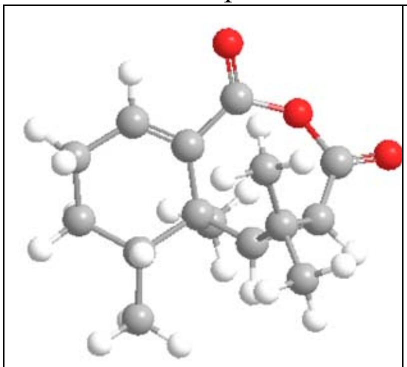	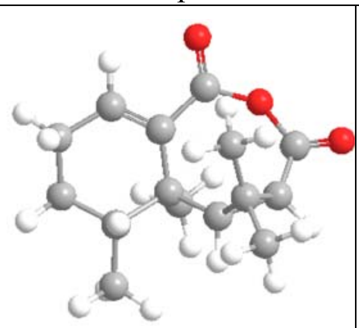	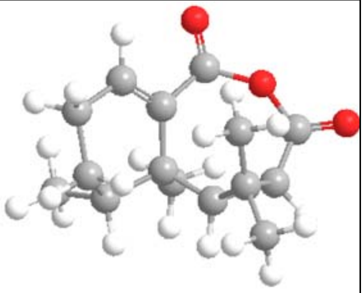
18aC1 (58.36%)	18aC2 (38.37%)	18aC1 (3.27%)

Table S3. ^{1}H NMR (400.13 MHz, $CDCl_3$) data for aristolane-type sesquiterpenoids (1)

	3	6
No.	δ_H (*J* in Hz)	δ_H (*J* in Hz)
1	3.47, d (2.4)	6.16, br d (10.4)
2	2.02, m	6.12, br d (10.4)
	1.63, m	
3	1.37, m	4.04, d (9.6)
	1.08, m	
4	1.53, m	1.76, m
6	0.77, d (9.2)	1.43, d (8.0)
7	0.87, dd (4.0, 9.6)	1.81, d (8.0)
8	2.31, m	—
	1.40, m	
9	3.97, dd (6.8,12.8)	5.79, s
11	—	—
12	1.03, s	1.24, s
13	1.07, s	1.17, s
14	1.10, s	1.14, s
15	0.90, d (6.8)	1.27, d (6.8)

Table S4. ^{1}H NMR (400.13 MHz, $CDCl_3$) data for aristolane-type sesquiterpenoids (2)

	7	14	18[a]
No.	δ_H (*J* in Hz)	δ_H (*J* in Hz)	δ_H (*J* in Hz)
1	6.98, d (10.0)	6.12 (1H, s)	6.65 (1H, t, 3.9)
2	6.18, d (10.0)	—	2.15 (2H, m)
3	—	2.25 (2H, m)	1.82 (1H, td, 6.7, 3.1)<br>1.45 (1H, m)
4	2.83, q (6.8)	2.33 (1H, m)	2.10 (1H, m)
6	1.51, d (8.0)	0.67 (1H, d, 9.1)	1.37 (1H, d, 9.9)
7	1.91, dd (1.2, 8.0)	1.42 (1H, m)	1.42 (1H, d, 9.9)
8	—	2.49 (1H, m)<br>1.47 (1H, m)	—
9	6.08, s	4.47 (1H, ddd, 11.9, 7.6, 1.8)	—
12	1.28, s	0.96 s	—
13	1.26, s	1.02 s	1.44 (3H, s)
14	1.24, s	1.26 s	1.18 (3H, s)
15	1.30, d (6.8)	1.06 (d, 6.4)	1.33 (3H, s)
16	—	—	0.89 (3H, d, 6.9)

[a] Measured in CD_3OD

Table S5. ^{1}H NMR (400.13 MHz, $CDCl_3$) data for nardosinone-type sesquiterpenoids (1)

	**19**	**22**[a]	**23**	**24**[a]
No.	δ_H (J in Hz)	δ_H (J in Hz)	δ_H (J in Hz)	δ_H (J in Hz)
1	3.92, br s	6.46, d (4.8)	7.10, dd (3.6, 4.2)	6.52, br s
2	2.16, m	4.08, m	2.33, m	2.22, m
	1.79, m			
3	1.44, m	1.61, ddd (4.4, 12.8, 14.0)	1.65, m	1.43, m
	1.19, m	1.48, d (12.8)	1.57, m	
4	1.69, m	2.48, m	2.41, m	2.68, m
6	3.03, d (8.4)	2.24, d (3.6)	2.78, d (1.2)	2.30, s
7	4.92, dt (2.0, 8.0)	4.42, m	—	—
8	3.05, dd (7.6, 18.0)	2.72, dd (18.0, 12.0)	3.46, d (21.0)	5.16, s
	2.76, dd (2.0, 18.0)	2.64, dd (18.0, 6.4)	3.33, dd (1.8, 21.0)	
12	1.40, s	1.27, s	1.33, s	0.97, s
13	1.29, s	1.35, s	1.36, s	1.10, s
14	1.20, s	0.88, s	0.90, s	0.89, s
15	0.91, d (6.8)	0.90, d (5.6)	0.98, d (6.8)	0.89, d (6.0)
-OH	—	6.03, d (7.2)	—	10.63, br s
-OH	—	5.62, s	—	4.32, br s
-OH	—	4.90, d (3.6)	—	—

[a] Measured in DMSO-d_6

Table S6. ^{1}H NMR (400.13 MHz, $CDCl_3$) data for nardosinone-type sesquiterpenoids (2)

	**26**	**30**
No.	δ_H (J in Hz)	δ_H (J in Hz)
1	6.78, s	3.70, br d (2.0)
2	—	2.13, m
		1.75, m
3	2.38, d (7.2)	1.45, dd (3.6, 12.0)
	2.37, d (11.6)	1.12, m
4	3.27, m	1.50, m
6	2.77, d (6.8)	3.33, d (6.8)
7	7.13, dd (6.8, 10.0)	6.86, dd (6.8, 10.0)
8	6.35, d (10.0)	6.27, d (10.0)
12	1.12, s	5.01, s
		4.96, s
13	1.30, s	1.59, s
14	1.25, s	1.13, s
15	1.13, d (6.8)	0.81, d (6.4)

Table S7. Effects of the compounds identified from NCB on SERT activity

Compound	Concentrations (μM)[a]				
	—	0.1	1.0	2.0	10.0
**1**	—	1.04±0.02	1.06±0.03	—	1.03±0.02
**2**	—	1.13±0.05**	1.26±0.05***	—	1.41±0.03***
**4**	—	1.23±0.03***	1.26±0.02***	—	1.21±0.03***
**5**	—	0.93±0.04*	0.87±0.03***	—	0.36±0.02***
**6**	—	1.02±0.01	1.06±0.02*	—	1.08±0.03**
**7**	—	0.97±0.01	1.06±0.02**	—	1.09±0.02***
**8**	—	1.06±0.02	1.09±0.02**	—	1.13±0.03***
**9**	—	1.01±0.02	1.04±0.01	—	1.07±0.02
**11**	—	1.06±0.01*	1.03±0.02	—	1.04±0.02
**12**	—	0.97±0.01	1.03±0.01	—	0.95±0.01**
**13**	—	0.94±0.02**	0.96±0.01*	—	0.93±0.02***
**15**	—	1.01±0.02	1.04±0.02	—	1.04±0.01
**16**	—	1.01±0.03	0.99±0.02	—	1.14±0.01***
**17**	—	0.96±0.01**	0.96±0.01*	—	0.93±0.01***
**18**	—	0.99±0.02	0.98±0.01	—	0.97±0.02
**19**	—	1.01±0.01	0.99±0.02	—	1.09±0.02***
**20**	—	0.61±0.04***	0.70±0.03***	—	0.89±0.02***
**21**	—	0.56±0.05***	0.65±0.03***	—	0.78±0.03***
**22**	—	1.06±0.02	1.03±0.02	—	1.04±0.02
**23**	—	0.96±0.01*	1.05±0.01**	—	1.08±0.02***
**24**	—	1.06±0.02	1.11±0.02***	—	1.12±0.02***
**25**	—	0.97±0.01	0.97±0.02	—	0.98±0.01
**26**	—	0.97±0.01	1.00±0.02	—	1.01±0.01
**27**	—	1.09±0.02*	1.19±0.02***	—	1.22±0.04***
**28**	—	1.08±0.02**	1.10±0.01***	—	1.09±0.02***
**29**	—	1.06±0.04	1.11±0.06**	—	1.16±0.04***
**30**	—	0.97±0.01	0.92±0.01***	—	0.93±0.02***
**31**	—	1.00±0.02	1.02±0.01	—	1.03±0.02
**32**	—	1.05±0.01*	1.08±0.02***	—	1.07±0.02***
**33**	—	1.07±0.01**	1.05±0.01	—	1.08±0.02**
**34**	—	1.01±0.02	1.02±0.01	—	1.00±0.03
**35**	—	0.97±0.01	0.96±0.02*	—	1.01±0.01
**36**	—	1.26±0.02***	1.31±0.02***	—	1.53±0.05***
**37**	—	0.97±0.01*	0.96±0.01**	—	0.97±0.01*
**38**	—	1.25±0.03***	1.27±0.05***	—	1.35±0.02***
**40**	—	1.01±0.01	1.05±0.01**	—	1.00±0.01
Fluoxetine	—	—	—	0.26±0.01***	—
Tianeptine	—	—	1.18±0.02***	—	—
Control	1.00±0.01	—	—	—	—

[a] The values represent the mean ± S.E.M. of relative fluorescent intensity (RFI) from triplicate assays (n≥9). RFI = (Intracellular APP^+ fluorescent $intensity_{treatment}$ /Intracellular APP^+ fluorescent $intensity_{control}$), *, p <0.05; **, p <0.01; ***, p <0.001.
